# Universal rhythmic architecture uncovers two modes of neural dynamics

**DOI:** 10.1038/s41467-026-73553-8

**Published:** 2026-05-30

**Authors:** Golan Karvat, Maité Crespo-García, Gal Vishne, Michael C. Anderson, Ayelet N. Landau

**Affiliations:** 1 MRC Cognition and Brain Sciences Unit, University of Cambridge, Cambridge, CB2 7EF UK; 2 Edmond and Lily Safra Center for brain sciences, The Hebrew University of Jerusalem, Jerusalem, 9190401 Israel; 3 Department of Psychology, The Hebrew University of Jerusalem, Jerusalem, 9190401 Israel; 4 Department of Cognitive and Brain Sciences, The Hebrew University of Jerusalem, Jerusalem, 9190401 Israel; 5 Department of Experimental Psychology, University College London (UCL), London, WC1H 0AP UK; 6 Present Address: Data Science Institute, Columbia University, New York, 10027 NY USA

**Keywords:** Psychology, Neuroscience, Cognitive neuroscience, Neuronal physiology

## Abstract

Understanding the organizing principles of brain activity can advance neurotechnology and medical diagnosis. Traditionally, neural activity is viewed as consisting oscillations in distinct frequency bands. However, emerging evidence suggests these oscillations often manifest as transient bursts rather than sustained rhythms. We examine the hypothesis that rhythmicity (sustained vs bursty) adds a further dimension to brain organization. Using a rhythmicity measure, we segment neurophysiological spectra from 859 participants across datasets, species, recording techniques, ages 18–88, sexes, brain regions, and cognitive states in health and disease. Our results reveal a universal rhythmicity-resolved spectral architecture with two categories: high-rhythmicity bands exhibiting sustained oscillations and new low-rhythmicity bands dominated by brief bursts. This architecture reflects two modes of operation: sustained bands suitable for maintaining ongoing activity, and transient bands which can signal responses to change. The rhythmicity-resolved architecture provides a unifying framework that bridges human and non-human findings, enables individualized spectral definitions, and offers a principled basis for understanding brain activity.

## Abstract (web summary)

Brain oscillations are traditionally regarded as sustained rhythms. Here, the authors show that there are both high and low-rhythmicity bands, characterised by brief bursts, which can signal responses to change.

## Introduction

Understanding the organizing principles of the brain’s electrical activity using non-invasive techniques is a major goal of neuroscience, with implications for brain-computer interface (BCI) design, pathology diagnosis, and treatment. A dominant hallmark of the electrophysiological signal is its tendency to oscillate. Brain oscillations are thought to signify synchronized fluctuations in excitability, present in neuronal systems from rodents to humans, and can be detected invasively or non-invasively^1,2^. According to a widely held viewpoint, the entire electrophysiological spectrum is composed of oscillatory bands, covering all frequencies from 0.1 to more than 100 Hz^3^ [the canonical bands delta (0.1–4 Hz), theta (4–8 Hz), alpha (8–13 Hz), beta (13–30 Hz), gamma (30–80 Hz), and high-frequency oscillations (80–250 Hz)]. A century of research and thousands of studies suggest that these seamlessly progressing bands form the spectral architecture of the brain, with oscillatory activity in different bands supporting distinct cognitive and physiological states^4,5^. This spectral architecture is now considered as one of the fundamental principles of neuronal activity^6^. However, several practical, functional, and physiological considerations challenge this viewpoint, calling for a revision of this architecture.

On the practical level, band definitions vary widely between research-groups^7^, which limits our ability to compare findings between studies and species. In addition, bands are currently defined and identified by sustained oscillatory activity and, as such, are assumed to progress seamlessly at the group level. However, at the single-subject level, which is vital for individualized neurotechnology, diagnosis, and treatment, these relatively wide bands consist of a combination of aperiodic activity and some narrow band oscillations^8^. With no objective framework for relating non oscillatory activity to canonical frequency bands, the role of activity expressed in the gaps between narrow band spectral peaks, if any, remains unknown. On the functional level, at least two of the canonical bands (beta and gamma) are thought to encompass within their range sub-bands with distinct—and even opposing—roles^9–11^, indicating the need for a more nuanced characterization of bands. On the physiological level, increasing evidence indicates that some oscillatory responses are not sustained. Rather, they arise as strong, short-lived bursts^12–14^.

The existence of bursts raises the possibility that the electrophysiological spectrum segregates into specialised frequency bands according to their persistence, i.e., sustained rhythms versus bursty spectral phenomena in different frequency bands. We therefore hypothesized that neural activity is organized within a rhythmicity-resolved spectral architecture. This architecture is defined not only by frequency but also by a rhythmicity axis that distinguishes between sustained (high-rhythmicity) and bursty (low-rhythmicity) modes of activity. This spectral segregation carries functional significance: it allocates specialized frequency bands to support two distinct processes—the maintenance of current state, which we hypothesize to be reflected by sustained oscillations in high-rhythmicity bands^5,15^, and transient responses to inputs, manifested as oscillatory bursts in low-rhythmicity bands^16,17^.

This rhythmicity-resolved spectral architecture hypothesis yields four testable predictions: (i) There should be defined frequency ‘sustained bands’ that clearly express sustained oscillations. (ii) Additionally, there should be a separate category of bands specializing in transient activity. In these ‘transient bands’, spectral phenomena should manifest as short-lived bursts, and critically, transient inputs should modulate these bands selectively. (iii) The architecture is a universal organizing principle of the brain’s operating system; hence it should arise across datasets, recording techniques, brain areas, and species. (iv) If the architecture mediates cognition, its distinct modes of operation should differentially respond to varying cognitive states and input processing.

To test this hypothesis and examine its predictions, we develop an objective standard to divide an individual’s electrophysiological spectrum into high-rhythmic and low-rhythmic bands. We then show that the resulting rhythmicity-resolved spectral architecture is universal and bears functional significance: rhythmic bands signify maintenance of ongoing activity, whereas non-rhythmic bands signify transient events.

## Results

### Rhythmicity-resolved spectral architecture

**Fig. 1:**
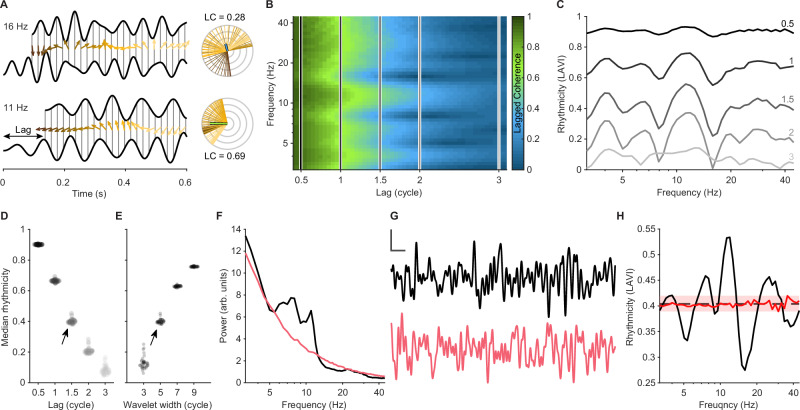
LAVI is a measure of rhythmicity. **A** To compute the Lagged Coherence (LC) at each frequency, the phase at each time point is subtracted from the phase of a lagged copy of the signal (arrows, color-coded by time). LC is the vector mean of phase differences across all time points (right, circular histograms). Rhythmic signals yield consistent phase differences and thus high LC values; non-rhythmic signals yield lower LC values. **B** LC (colour) as a function of lag (abscissa) and frequency (ordinate) of one participant. Vertical lines correspond to specific lags plotted in **C**. **C** LC dynamics across lags. Peaks at specific frequencies are found in different lags, with ceiling/floor effects for very short or long lags. To avoid confusion between LC (computed over different lags) and rhythmicity in different frequencies at a specific lag, we term this value Lagged Angle Vector Index (LAVI). **D** The LAVI median is consistent across participants. Shown are the medians (across frequencies) of each of the *N* = 37 participants from Dataset I (dots; shades of grey as in C), plotted as a function of lag. For all lags, a wavelet width of 5 cycles was used. Note the relatively low variability across participants at each lag. **E** As in **D**, but with varying wavelet widths. Lag was fixed at 1.5 cycles for all participants. Arrows in D and E indicate the parameter values used throughout the manuscript (1.5 cycles lag and 5 cycles wavelet, correspondingly). **F** Power spectral density of one subject (black) and the aperiodic component estimated using a power-law fit (pink). This fit was used to generate surrogate data with a similar power spectrum but randomized phase structure. **G** Raw traces from the original (black) and surrogate (pink) data. Vertical scale bar: 10 μV; horizontal scale bar: 0.1 s. **H** LAVI of real (black) and surrogate (pink) data. Rhythmic peaks and troughs are evident in the real data but absent in the surrogate. Dashed line: median of data. Pink shade: noise ribbon, defined as the range of the surrogate. Source data are provided as a Source Data file.

To test the predictions of the rhythmic architecture hypothesis, we developed a phase-based tool to measure rhythmicity over the frequency spectrum (Figs. 1 and  S1, and online methods). Three features characterize brain oscillations: frequency (cycles per time-unit), power (or magnitude), and phase (or angle). The canonical bands are typically identified by measuring spectral power at different frequencies (Fig. S2A-C). However, estimated power is influenced by both oscillatory activity and non-oscillatory transients. For example, a spectral power peak in a wide range of frequencies could result from transient impulses which are not rhythmic. An alternative approach to power is to estimate how sustained the oscillation is by measuring how well, at any moment, the signal’s phase predicts its future phase. This is achieved by computing the coherence, or phase similarity, between the signal and a time-lagged copy of itself (Fig. 1A). Previous authors defined rhythmicity as “the consistency of the phase relations between timepoints that are separated by some interval (lag)^18^.” Then, they measured the rhythmicity spectrum as a function of lag duration, assuming that genuine oscillations should remain sustained for many cycles^18–20^ (Fig. 1B). Here we adopt this definition of rhythmicity, and note that the term is not meant to imply the existence of an underlying rhythm-generating circuit, but rather denotes the degree of phase consistency. Based on large datasets and comprehensive simulations, we found that the choice of lag does not influence the relative rhythmicity values obtained across the different frequencies for each individual subject (Fig. 1C). Hence the rhythmicity spectrum can be derived from a single lag.

We term the algorithm used to resolve rhythmicity across different frequencies at a specific lag the *lagged-angle vector index* (LAVI). LAVI is defined as the vector mean, computed across all time points, of the phase difference between the signal and a lagged copy of itself. As a measure of phase-consistency, it is influenced by the rate of phase-shift occurrence (Fig. S1). Importantly, LAVI values show a clear baseline: the median across frequencies is stable across participants, channels, and datasets, and depends only on experimenter-controlled parameters such as lag duration (Fig. 1D) and the window size used for spectral estimation (Fig. 1E). To test whether the LAVI spectrum reflects background noise, we generated surrogate datasets. These were constructed by randomizing phases while matching each dataset’s 1/*f* power profile—the inverse relationship between power and frequency characteristic of neurophysiological signals (see Methods; Fig. 1F–H). Recorded and surrogate data had similar median rhythmicity values, but the surrogates showed a narrower, flatter range. Leveraging this property, we define the median across frequencies as baseline rhythmicity and the surrogate range as the noise ribbon (see Methods and Supplementary Text; Fig. S6).

**Fig. 2:**
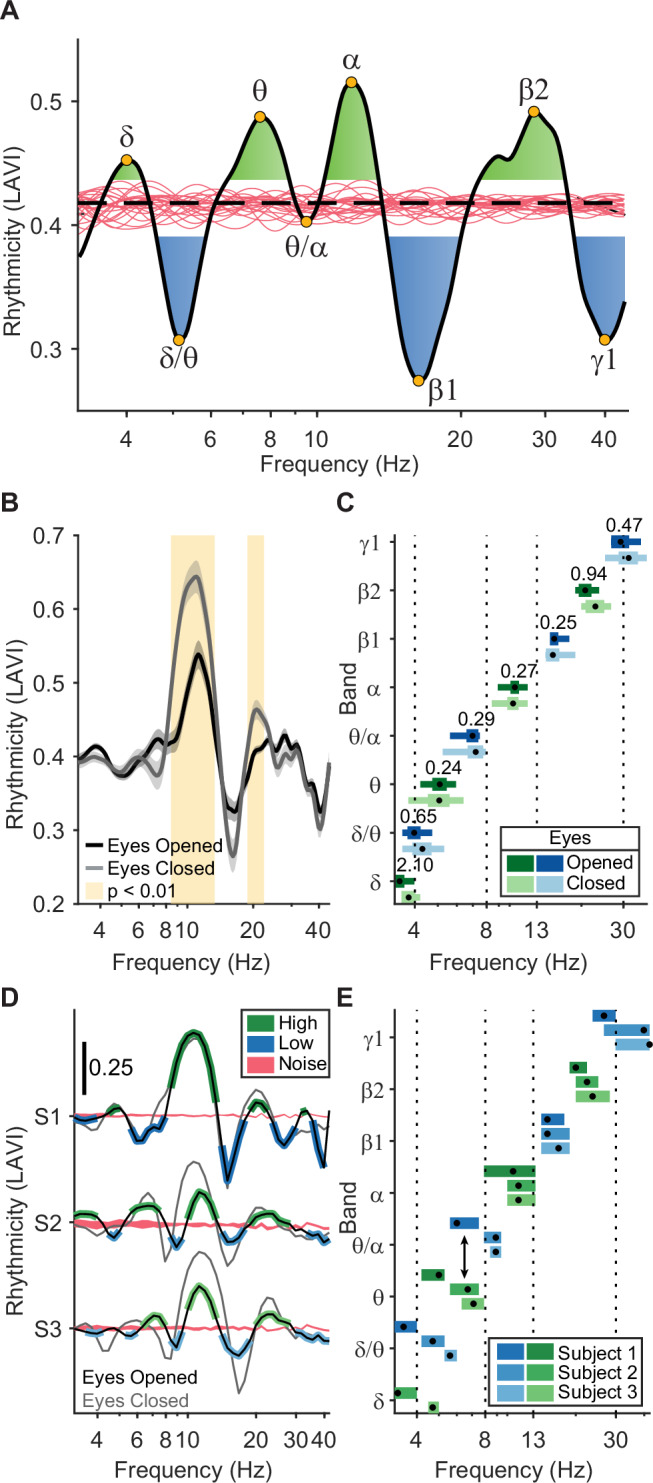
ABBA detects individual and group-level brainwave bands. **A** Rhythmicity profile of a representative participant, with output of the Automated Band Border Algorithm (ABBA). Black line: subject’s rhythmicity; dashed line: median; pink traces: 20 instances of simulated 1/*f* noise. Yellow circles mark peaks and troughs. Green and blue areas indicate frequencies with significantly increased or decreased rhythmicity, respectively. **B** Group-level rhythmicity profiles (*N* = 37 participants) for eyes open (black, dataset I) and closed (grey, dataset II). Shading: SEM; yellow highlights significant clusters (*p* < 0.01, cluster-based permutation analysis of two-sided *t*-tests). **C** ABBA-derived group-level band annotations (*N* = 37, same as **B**). Dots: mean peak frequency; thin horizontal lines: interquartile range of high- (green) and low-rhythmicity (blue) bands; thick horizontal lines: 95% confidence intervals. Bayes factors (black numbers above each band) quantify group differences between eyes open and closed (BF < 1: no difference). Dashed lines indicate canonical (power-based) bands. Note: While the brain states affect rhythmicity magnitude, the automatically detected band frequencies remain stable. **D** Rhythmicity profiles from three participants during eyes-open (black) and eyes-closed (grey) conditions. Pink: rhythmicity profiles from 1/*f*-matched random-phase simulations. Subject-specific ABBA-identified bands with high (green) or low (blue) rhythmicity during eyes-open session are overlaid. **E** Automatically detected bands for the same three participants as in **D**. Dots show peak frequencies; horizontal lines span detected band ranges (green: high rhythmicity; blue: low). Note individual variability, including cases where the same frequency is classified differently across subjects (arrow). Source data are provided as a Source Data file.

The use of a single lag in LAVI not only accelerates computation by an order of magnitude compared to previous lagged-coherence methods, but also establishes a universal baseline of rhythmicity. This baseline enables the identification of frequency bands exhibiting both increased and decreased rhythmicity, within and across individuals. LAVI is therefore uniquely suited to test our rhythmic-architecture hypothesis by identifying frequency ranges that lie between two crossings of the baseline and above the noise ribbon as high-rhythmicity bands. Conversely, frequencies between two baseline crossings that fall below the noise ribbon are classified as low-rhythmicity bands (Fig. 2A). Integrating LAVI with simulations of surrogate distributions (Fig. 1F-H), we developed an *automated band-border detection algorithm* (ABBA), which delineates the boundaries of significantly high- and low-rhythmicity bands at the individual subject level. Applying ABBA revealed a considerable measure of consistency across subjects, suggesting a universal rhythmic architecture, while maintaining sensitivity to inter-individual differences within this universality.

Supporting universality, peak frequencies of high-rhythmicity bands defined by ABBA across participants were consistently confined to the canonical, power-based frequency bands (Fig. 2B–C)^3^. In particular, we consistently observed a dominant peak within the 6–14 Hz range, aligned with the traditional alpha band, allowing for automatic annotation of band labels. We label this peak as ‘alpha’ (α); the subsequent trough is labelled ‘beta1’ (β1), followed by the next peak as ‘beta2’ (β2), and the following trough as ‘gamma1’ (γ1). Similarly, the trough preceding alpha is labelled as ‘theta/alpha’ (θ/α), the adjacent lower-frequency peak as ‘theta’ (θ), the next lower trough as ‘delta/theta’ (δ/θ) and the lowest peak considered in this manuscript is labelled ‘delta’ (δ) (see Fig. 2A). Importantly, within the universal framework, individual subjects showed considerable variability, with some frequencies classified as high- or low-rhythmicity depending on the subject (Figs. 2D–E and  S2G). This variability underscores the need for an objective, single-subject band-detection framework. Our approach thus enables comparison with canonical band names while capturing the individualized spectral structure that underlies them.

This alignment also enabled us to explore the functional relevance of the spectral architecture and to test our prediction that rhythmicity patterns are modulated by cognitive states. To this end, we examined whether commonly observed changes in oscillatory power across brain states are also reflected in rhythmicity. Specifically, we compared the rhythmicity profiles of the same participants while they were engaged in a visual task and during rest with eyes closed (Fig. 2D–E). Alpha-band power is known to increase during wakeful rest with eyes closed^21^. Consistent with this, we found that rhythmicity in the alpha-band (8.4–13.3 Hz) differed significantly between wakeful rest and the visual task (*p* < 0.01, cluster-permutation test, Fig. 2D). Moreover, the change in rhythmicity (measured via LAVI) was strongly correlated with the change in alpha power (measured in dB, ρ = 0.781, *p* < 10^−7^). A significant increase in rhythmicity during rest with eyes-closed was also observed in the beta2 band (18.8–22.4 Hz), but not in beta1 (14.7-17.8 Hz), suggesting distinct functional roles for these sub-bands. Notably, the frequency ranges of bands identified by ABBA, as reflecting high or low rhythmicity, remained stable across states (*t*_295_ = 1.4, *p* = 0.16, BF_10_ = 0.17), underscoring the robustness of the spectral architecture.

### Low- and high-rhythmicity bands show distinct burst dynamics

Our findings point to a stable spectral architecture defined by frequency and rhythmicity, with distinct functional roles for high-rhythmicity bands. Traditional power-based band-detection approaches often overlook low-rhythmicity bands—but do these bands represent physiologically meaningful processes? Or are they merely trivial by-products of rhythmicity peaks? In order to shed light on this question, we turned to sub-second manifestations of the spectral architecture. Specifically, we investigated a putative relationship between rhythmicity and burst dynamics in EEG data. Because rhythmicity was estimated at the session level, this analysis allowed us to test whether large-scale rhythmic organization is reflected locally within transient bursts of activity.

**Fig. 3:**
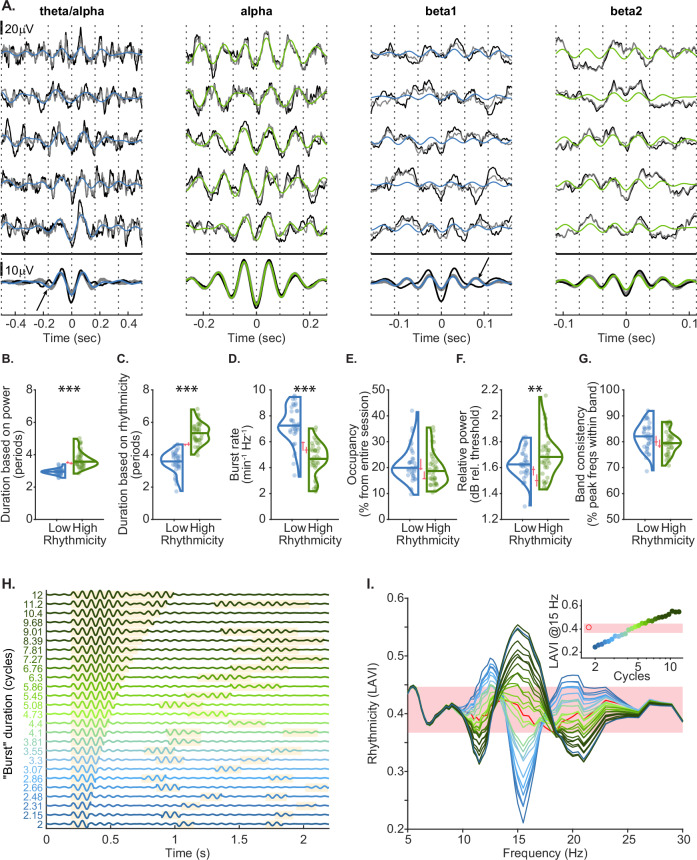
Bursts affect rhythmicity. **A** Raw bursts in two low-rhythmicity bands (θ/α, β1) and two high-rhythmicity bands (α, β2), aligned to the trough nearest max power. Black: raw signal (one representative subject). Blue/green: band-pass–filtered. Grey: band-stop filtered (neighbouring bands removed). Bottom row: average across bursts (“burst-related potential”, BRP). Grey traces are plotted thicker for visibility. Dotted lines mark troughs; arrows indicate phase shifts, likely due to neighbouring high-rhythmicity bands (see Fig. S5 for control analyses). **B–G** Burst statistics across low- and high-rhythmicity bands (*N* = 37). Dots: participant means; violins: full distributions; horizontal lines: medians. Pink elements: medians and CIs from 1/*f*-matched random-phase simulations. **B** Duration based on power > 75th percentile. Two-sided *t-*test (low- vs. high-rhythmicity): *t*_72_ = 8.35, CI = [0.61, 0.99], Cohen’s *d* (effect size)=1.94, *p* = 3.4 × 10^−12^ (3.4 × 10^−12^ after FDR correction for multiple comparisons in **B**–**G**). **C** Duration based on rhythmicity (Within-Trial Phase Lock; WTPL > 75th percentile). Two-sided *t*_72_ = 11.32, CI = [1.53, 2.18], *d* = 2.63, *p* = 1.23 × 10^−17^ (7.37 × 10^−17^). **D** Bursts per minute. Two-sided *t*_72_ = 8.62, CI = [2.15, 3.43], *d* = 2.00, *p* = 1.07 × 10^−12^ (3.22 × 10^−12^). **E** Occupancy (proportion of time in bursts). Two-sided *t*_72_ = 1.23, CI = [–1.11, 5.12], *d* = 0.30, *p* = 0.20 (0.20). **F** Peak-power relative to the 90th percentile threshold. Two-sided *t*_72_ = 2.13, CI = [0.002, 0.048], *d* = 0.50, *p* = 0.036 (0.043). **G** Frequency overlap (proportion of burst time with peak frequency in-band). Two-sided *t*_72_ = 2.44, CI = [0.48, 4.72], *d* = 0.57, *p* = 0.0171 (0.0256). **p < 0.01; ***p < 0.001. See Supplementary Table 1 for ANOVA including pink-noise simulations. **H** Simulated bursts: pink noise was generated from 1/*f* filtered white-noise. At 15 Hz, filter weight doubled at time-points chosen as bursts (variable length, yellow shading), halved otherwise. **I** Rhythmicity profiles of simulated data. Trace colour: burst duration (corresponding to **H** and the inset). Pink shading: LAVI range for unmodulated pink noise; red: unadjusted 1/*f* noise. Inset: LAVI at 15 Hz. Source data are provided as a Source Data file.

We defined bursts as power peaks exceeding the 90th percentile (see Methods). Visual inspection at the single-subject level revealed that bursts occurring in high-rhythmicity bands (alpha, beta2) typically showed oscillations sustaining several cycles, with close correspondence between raw and filtered signals. In contrast, bursts within low-rhythmicity bands (theta/alpha, beta1) dampened quickly and displayed phase deviations between raw and filtered traces (Fig. 3A), indicating that bands characterized by lower rhythmicity predominantly contain short, weakly phase-consistent bursts.

At the population level (*N* = 37 subjects; see also Fig. S3 for analysis across six EEG datasets (total of 178 subjects), bursts in high-rhythmicity bands lasted significantly longer (3.5 vs. 2.9 cycles), a pattern amplified when using Within-Trial Phase-Lock (WTPL^22^), a time-resolved measure of phase-consistency (5.3 vs. 3.6 cycles; Fig. 3B-C. See Supplementary Table S1 for full statistical details). Conversely, low-rhythmicity bursts occurred more frequently (Fig. 3D), resulting in comparable total durations during which signals exceeded burst thresholds across bands (Fig. 3E), with high-rhythmicity bursts showing slightly higher normalized amplitudes (Fig. 3F). Moreover, although instantaneous frequencies can drift within bursts^17,23^ (Fig. S4), in both modes over 80% of peak frequencies during bursts were confined within the LAVI-defined frequency band (Fig. 3G).

To rule out the possibility that these effects arose from 1/*f* (“pink”) noise, we compared the EEG data to phase-randomized surrogate signals with matched 1/*f* power profiles. The simulated signals showed no differences in burst rate or duration across frequencies (Fig. 3B–D, pink lines), and critically, the recorded bursts differed significantly from these noise simulations. In a separate set of simulations we generated bursts with varying length and quantified the resulting rhythmicity of the signal. Bursts shorter than 3.81 cycles yielded rhythmicity below chance levels, while bursts longer than 5.45 cycles exceeded it (Figs. 3H–I and  S4), resembling WTPL-based burst durations and supporting a distinction between transient and sustained rhythmicity. Together, these findings suggest that bursts are epochs of band-confined activity: low-rhythmicity bands reflect brief, transient bursts, whereas high-rhythmicity bands exhibit fewer but longer-lasting oscillations. This raises the question: does this rhythmic architecture hold universally across contexts and recording modalities?

### The rhythmic architecture is universal

Establishing the rhythmicity-resolved spectral architecture as a universal phenomenon requires a robust, diverse, and large sample. Therefore, we computed the rhythmicity spectra of data from 12 datasets acquired from 2450 recording sites of 11 rats and 848 humans (with both invasive and non-invasive methods; Table 1). Using LAVI and ABBA, we found the rhythmicity architecture consistently in all datasets, and across ages, sexes, species, brain regions, and recording techniques, in health and disease.

**Table 1:** Details of datasets used in this study

Dataset	Task/Population	Recording technique	*N* subjects	Recording sites	Age mean (range)	Ref.
I	Visual duration judgement	EEG	37 volunteers	Cz	22.9 (18−29)	^22^
II	Eyes closed	EEG	37 volunteers	Cz	22.9 (18−29)	^22^
III	Tactile duration judgement	EEG	29 volunteers	Cz	24 (19−31)	^66^
IV	Audio–visual illusion	EEG	24 volunteers	Cz	(18−42)	^67^
V	Memory-control	EEG	27 volunteers	Cz	25 (20−34)	^68^
VI	Memory-control	HD-EEG	24 volunteers	Cz	(18−35)	^69^
VII	TMS	EEG	6 volunteers	F4	27 (19−37)	New
VIII	Rest	MEG	625 volunteers	4 magnetometers surrounding Cz	(18−88)	^34^
IX	Epilepsy patients	ECoG	10 patients	867 electrodes (sites: Fig. 1I)	41 (19−65)	^70,71^
X	Epilepsy patients	EEG + SEEG	12 patients	733 electrodes (sites: Fig. 1J)	27.5 (17−39)	^72^
XI	PD patients	LFP	17 patients	STNs from 30 hemispheres	56.4 (38−65)	^73^
XII	Rats navigation	LFP	11 rats	Hippocampus		^30^

*EEG* electroencephalogram, *HD-EEG* high-definition (high-channel-count) EEG, *TMS* transcranial magnetic stimulation, *MEG* magnetoencephalogram, *ECoG* electrocorticography, *SEEG* stereoelectroencephalogram (depth electrodes), *LFP* local field potentials (depth electrodes), *Cz* midline central electrode, *F4* right frontal electrode, *Ref.* reference, *PD* Parkinson’s disease.

**Fig. 4:**
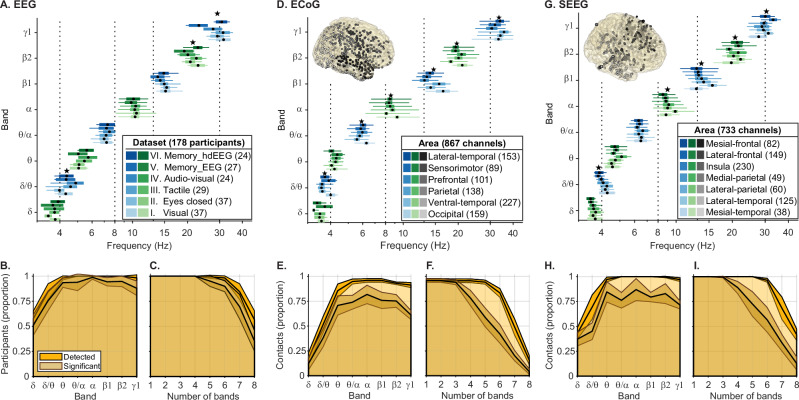
The rhythmicity architecture is universal. **A–C** Peaks and borders of rhythmicity bands across six EEG datasets (*N* = 178 participants). **A** Mean peak frequencies (dots) of high- (green) and low-rhythmicity (blue) bands; thick horizontal bars: 95% CI of the peaks; thin horizontal bars span from the mean of the lowest to the mean of the highest frequency in each band; stars: significant differences across datasets (*p* < 0.05, ANOVA with Fisher’s least significant difference post-hoc procedure). The same plotting conventions apply to panels **D** and **G**. See Supplementary Table 1 for full statistical details. **B** Proportion of participants with each band detected (brown) and statistically significant (yellow); lines: mean; shading: ±SD across studies. **C** Cumulative proportion of participants with ≥x detected bands; colour as in **B**. **D–F** Same metrics for 867 ECoG contacts from 10 patients across six brain regions (dataset IX; legend in **D** shows region names and electrode counts; SD in **E**–**F** over regions). **G–I** Same for 733 SEEG contacts from 12 patients across seven regions (dataset X). Source data are provided as a Source Data file.

To determine whether the rhythmic architecture generalizes across recording modalities and brain regions, we analysed spectral profiles from a large dataset comprising both invasive (ECoG, SEEG) and non-invasive (EEG) recordings. We compared the distributions of peak frequencies across datasets (EEG, Fig. 4A–C) and neuroanatomical locations (ECoG, Fig. 4D–F; SEEG, Fig. 4G–I). Using ABBA, we consistently detected the canonical sustained bands—θ, α, and β2—as well as the transient bands—θ/α, β1, and γ1—in nearly all participants (Fig. 4B, 4E, 4H). Detection rates were lower for δ and δ/θ, likely due to our choice of 3 Hz as the lowest frequency analysed, which might not capture δ and δ/θ rhythmicity, resulting in a noisy estimate at these frequencies (i.e., the actual peaks may be in lower frequencies than measured). Importantly, for most participants and electrodes, at least 6 out of 8 bands were detected (Fig. 4C, 4F, 4I). To assess the statistical significance of these bands efficiently, and based on characterization of the parameters affecting the noise ribbon (Fig. S6), we first generated 200 random-phase surrogate datasets to construct a reference table of null distributions, matched to a range of 1/*f* power profiles and acquisition parameters. For each participant and electrode, we then selected the appropriate thresholds from this table based on their individual power spectra, session duration and sampling-frequency. These thresholds were applied within a Monte Carlo framework to determine which observed bands exceeded chance levels. Across the 3–45 Hz range and at α = 0.05, the vast majority of detected bands were found to be statistically significant. Notably, while differences in peak frequencies between datasets and brain regions were observed within bands, the boundaries between bands remained stable—indicating that the rhythmic architecture is robust.

**Fig. 5:**
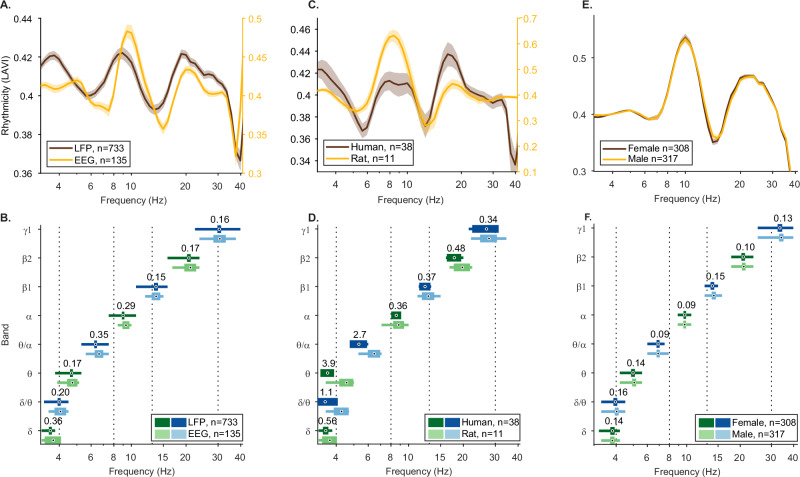
Rhythmicity reduces uncertainties in existing data. **A, B** Lagged Angle Vector Index (LAVI, **A**) and Automated Band Border Algorithm (ABBA, **B**) analyses of concurrently recorded LFP (brown) and EEG (yellow) in patients (*N* = 135 EEG and 733 LFP electrodes from 12 patients, dataset X) reveal that population averaged EEG band peaks tend to be faster (rightward shift of yellow vs. brown line). However, at the individual level, Bayes factor values below 1 indicate similarity between recording methods. **C, D** Comparison between human hippocampal SEEG (*N* = 38 electrodes from 12 patients, dataset X) and rat hippocampal LFP (*N* = 11 rats, dataset XII). Bayes factor analysis provides evidence for no difference between species in Alpha, Beta1, Beta2, and Gamma1 bands. **E, F** The rhythmicity architecture of female and male participants from dataset VIII (*N* = 308 female and 317 male participants) is similar. In **A**, **C**, and **E** lines indicate means and shaded areas represent SEM; numbers in parentheses denote electrode counts. In (**B**, **D**, **F**), dots mark mean peak frequencies per band, with thick horizontal bars showing 95% confidence intervals and thin bars the interquartile range. Black numbers above each band show between-group Bayes factors from *t*-tests. Source data are provided as a Source Data file.

These consistent patterns across modalities and regions suggest that the rhythmic architecture may help reduce uncertainties arising from methodological and species-related differences. For instance, whether frequency bands identified in invasive recordings such as ECoG or SEEG correspond directly to those observed in non-invasive EEG remains an open question, due to fundamental differences in spatial resolution, depth sensitivity, and susceptibility to signal distortions^1,24^. To address this, we leveraged a unique recording setting including both scalp EEG and invasive electrodes in dataset X, allowing us to directly compare rhythmicity profiles and individually defined bands between measurement modalities by comparing invasive signals to those from the nearest concurrently recorded scalp EEG electrode. We found that while the average frequency profile of scalp electrodes tended to be higher than the invasive counterpart (i.e., slightly faster; Fig. 5A) relative to invasive electrodes, the individual band peak frequencies identified using ABBA were remarkably similar across measurement modalities (BF_10_ = 0.042; BF_01_ = 23.8), providing strong evidence in favor of the null hypothesis of no difference between modalities (Fig. 5B).

Furthermore, the invasive recordings from patients’ hippocampi enabled a cross-species comparison of rhythmic architecture with rats. A longstanding debate concerns whether human alpha oscillations (8–12 Hz^3^) are functionally analogous to rodent theta (4–12 Hz^25^). While some argue these rhythms reflect similar mechanisms despite frequency differences^26,27^, others question whether rodents exhibit alpha at all, noting their hippocampal activity is dominated by theta^28,29^. To address this and test whether the rhythmicity architecture generalizes across species, we analyzed hippocampal recordings from 11 rats (Dataset XII). As in humans, both sustained and transient bands from θ to γ1 were detected in nearly all rats (100% for θ–γ1; significant in 100% for θ/α, α, and β1; 90% for β2; 82% for θ and γ1). Although rhythmicity magnitudes were higher in rats (note scale in Fig. 5C), band frequency distributions were strongly conserved across species (BF_01_ = 10.99, Fig. 5D). Notably, the dominant peak in rats was around 8 Hz—labelled ‘theta’ in the original publication of dataset XII^30^, but identified as ‘alpha’ by ABBA—suggesting that rodent theta reported in the literature may correspond to human alpha, as the most prominent rhythmic band below ~15 Hz. To further assess the generalizability of rhythmicity architecture, we examined potential sex differences in Dataset VIII. LAVI and ABBA analyses (Fig. 5E–F) showed comparable rhythmicity profiles and band peak frequencies between female and male participants, with strong evidence for no difference (BF_01_ = 43.25).

Taken together, these findings reveal a dual-mode rhythmicity architecture: sustained bands characterized by long-lasting oscillations, and transient bands dominated by brief bursts. This architecture generalizes across datasets, brain regions, recording modalities, sexes, and species, supporting the idea of a universal organizing principle. We now turn to our final prediction: that these distinct rhythmic modes should differentially reflect and respond to varying brain states and input processing demands.

### The rhythmic architecture reveals functional consequences of structural change

**Fig. 6:**
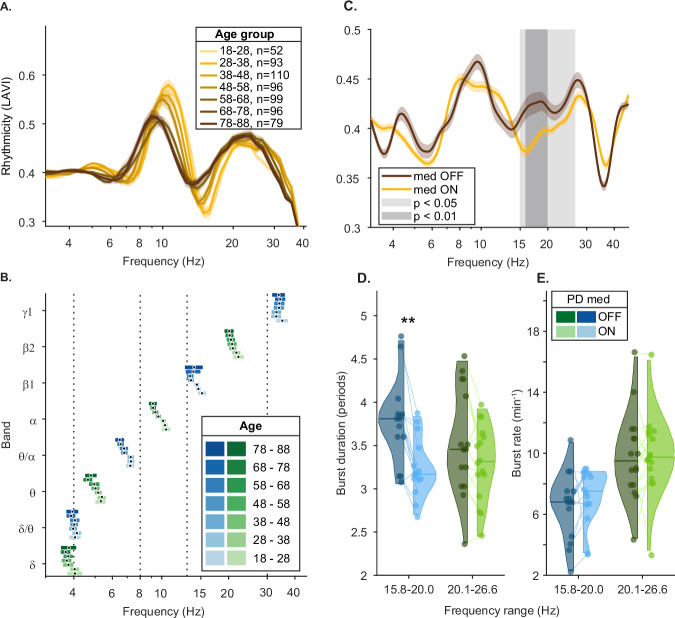
Rhythmicity changes in healthy-aging and disease. **A, B** Lagged Angle Vector Index (LAVI, **A**) and Automated Band Border Algorithm (ABBA, **B**) analyses over the adult lifespan (Dataset VIII). In **A**, lines indicate group means and shaded areas represent SEM; numbers denote participant count per age group (in years) and n number of participants. In **B**, dots represent mean peak frequencies, horizontal bars: 95% confidence intervals. Number of participants per age group is presented in the legend of panel **A**. **C** Population-averaged rhythmicity profiles (mean ± SEM) from Sub-thalamic Nuclei (STN) of PD patients (*N* = 17; 30 hemispheres, Dataset XI), comparing ON vs. OFF L-Dopa states. Grey shading: significant clusters from permutation analysis of two-sided *t*-tests. Burst duration (**D**), defined as power > 75th percentile, and number of bursts per minute (**E**) from hemispheres showing increased beta1 rhythmicity OFF vs. ON medication (15.8–20.0 Hz, median split). Dots represent individual STN means; violins show full distributions; horizontal bars denote medians. **p = 0.0026, two-way ANOVA with Fisher’s least significant difference (LSD) post-hoc. Source data are provided as a Source Data file.

To explore how rhythmic modes reflect physiological conditions of the brain, we first investigated age-related changes in rhythmicity magnitude and peak frequency. Aging is associated with widespread structural and functional alterations, many of which are linked to cognitive decline^31^. Neural oscillations, particularly in the alpha band (∼8–12 Hz), are known to decelerate and reduce power with age, correlating with reduced processing speed and diminished attentional control^32^. However, more recent studies suggest that such effects may partly stem from changes in the aperiodic background signal^33^. Aging thus provides a valuable testbed for examining whether the rhythmicity architecture captures these physiological changes. Because LAVI and ABBA are less influenced by variations in the 1/*f* slope—owing to their use of a robust baseline—they enable assessment of age-related changes without confounds that have limited previous analyses^33^. We analysed rhythmicity profiles from magnetoencephalography (MEG) recordings of a large cohort of healthy individuals spanning the adult lifespan (*N* = 625, ages 18–88; Dataset VIII^34^). We observed age-related reductions in rhythmicity magnitude and frequency not only in alpha but also in other sustained (delta, theta, and beta2) and transient bands (theta/alpha and beta1; Pearson correlation per participant and ANOVA per age decade group, Fig. 6A-B and Table S1). These findings indicate that both sustained and transient rhythmic modes are sensitive to age-related changes, suggesting that the rhythmicity-resolved architecture captures the brain’s evolving functional organization across the lifespan. This large-scale group characterization provides a normative reference for neuro-typical aging.

We next asked whether the rhythmic architecture might vary in neuropathological conditions, and help to identify the consequences of pathology. To address this, we compared rhythmicity profiles of LFP recordings from sub-thalamic nuclei (STN) of Parkinson’s disease (PD) patients, with (i.e., “ON”) and without (i.e., “OFF”) medication (*N* = 17 patients, 30 hemispheres; Dataset XI). In PD, beta power ( ~ 13–30 Hz) is known to increase^35^. Activity in the beta band is believed to be controlled by the level of dopaminergic activity in response to internal and external cues, and following dopaminergic cells’ loss, beta levels are elevated in PD^36^. We found a significant increase in rhythmicity OFF medication compared to ON in the entire beta-band (15.0–26.6 Hz, *p* < 0.05, cluster permutation test; Fig. 6C). Notably, using a permutation test with stricter threshold of *p* < 0.01 the medication effect was localized to the transient band beta1 (15.8–20.0 Hz, *p* < 0.01). This suggests that while PD pathology elevates rhythmicity across the beta range, the effect is particularly pronounced in the transient band beta1, implicating burst dynamics in the pathophysiology. Indeed, prior studies have linked increased STN beta power to prolonged beta bursts, which are shortened in response to medication^37,38^. To further explore whether the pathological burst dynamics are related to rhythmicity modes, we compared burst duration and rate in beta1 (15.8–20.0 Hz) and beta2 (20.1–26.6 Hz) from STNs exhibiting medication-related beta1 power modulation (median split). We found significantly longer burst durations OFF medication specifically in beta1 (*p* < 0.01; Fig. 6D). No significant differences were observed in burst durations for beta2 or in burst rates for either beta band (Fig. 6E; all *p* > 0.15). These findings from healthy aging and Parkinson’s disease illustrate the potential of our rhythmicity-resolved spectral architecture to illuminate critical functional consequences of changes in brain structure.

### Dual rhythmic modes and input processing

**Fig. 7:**
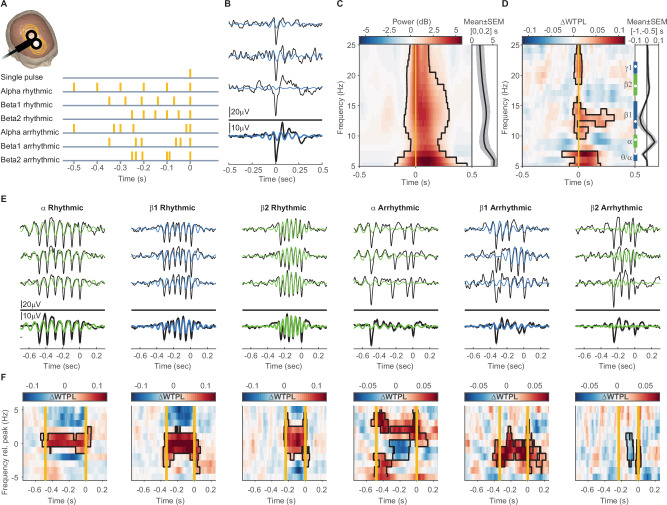
Band-specific responses to TMS stimulation. **A** Stimulation protocol. TMS over the right dorsolateral prefrontal cortex (rDLPFC) alternated randomly between single pulses and trains of six rhythmic or arrhythmic pulses (yellow vertical lines). Semi-transparent head surface mesh were created using the Fieldtrip^65^ function ft_plot_mesh, and data from Wang et al. ^99^ available at https://www.fieldtriptoolbox.org/tutorial/source/headmodel_eeg_bem/, licensed under CC BY-SA 4.0 (https://creativecommons.org/licenses/by-sa/4.0/). Modifications: rendered with custom transparency and colour settings. **B** Single-trial responses (top three traces) and the averaged response (bottom trace) to single-pulse stimulation from a representative participant. Black: raw signal; blue: signal filtered in the beta1 band (11.2–15.8 Hz). Shaded area represents ±SEM (barely visible due to low variation levels). Population averaged (*N* = 6) power (**C**) and Within-Trial Phase-Locking value (WTPL; **D**) at each frequency, normalized to a 0.5–1 s pre stimulation baseline. Trace plotted to the right of the panel depicts the mean ± SEM of power during the first 200 ms after TMS (**C**) or baseline rhythmicity during the [−1 −0.5] s baseline window (**D**). Blue and green vertical elements indicate the mean frequencies of transient and sustained bands across participants, respectively. White dots mark the mean peak frequency per band. Black contour denotes clusters with significant increased power or rhythmicity (*p* < 0.05, cluster-based permutation test over two-sided *t*-test values). Yellow line at time = 0: TMS single-pulse. Notably, following the TMS pulse, power increases in a wide range of frequencies, while WTPL values increase specifically at transient bands. **E, F** Repetitive TMS. **E** Single-trial responses (top three traces) and the averaged response (bottom trace). Black traces: raw signal; coloured time-series (green and blue) indicate signals filtered at the band used for rTMS. **F** WTPL. Yellow vertical lines mark the first and last TMS pulses. Frequencies (ordinate) are shown relative to the individual peak frequency of each participant in each band. Black contour: significant (*p* < 0.05) cluster, permutation analysis of two-sided *t*-tests. Source data are provided as a Source Data file.

To investigate how this architecture supports cognition, we next examined how its distinct modes operate on shorter timescales during input processing. Specifically, we asked whether sustained rhythms, being stable over many cycles, support ongoing maintenance, whereas transient rhythms, being brief and event-linked, reflect responsiveness to change. To test this, we used transcranial magnetic stimulation (TMS) to deliver transient (single-pulse) and repetitive (both rhythmic and arrhythmic) inputs while recording EEG (Fig. 7A).

Single TMS pulses evoked characteristic EEG responses consisting of a sequence of peaks and troughs (ripples; Fig. 7B). In the frequency domain, filtering transient responses (delta functions) can produce apparent broadband power increases. Indeed, consistent with previous studies^39^, this response was reflected in increases in the power across a wide range of frequencies (Fig. 7C). However, according to the rhythmic architecture framework, the rhythmic response to brief events should be confined to the transient bands. To test this, we measured time-resolved rhythmicity (WTPL), and found that ripples were associated with significantly increased rhythmicity specifically in the transient bands θ/α, β1, and γ1 (*p* < 0.05, cluster permutation test; Fig. 7D), lasting up to 300 ms post-stimulation.

For repetitive inputs, we predicted that rhythmic stimulation, delivered at a fixed inter-pulse interval (IPI), would enhance EEG-measured rhythmicity. In contrast, arrhythmic stimulation, with temporally irregular IPIs, was expected to violate phase consistency and thereby disrupt sustained oscillatory activity. To test this, we first determined each participant’s individual peak frequencies in the alpha, beta1, and beta2 bands. For each band, we delivered 60 rhythmic trains of six pulses, with IPIs matched to the cycle length of the band’s peak frequency. The arrhythmic condition consisted of temporally irregular pulse trains matched in overall duration. As with single pulses, each TMS pulse evoked a pronounced transient response (“ripple”; Fig. 7E). During rhythmic stimulation, these responses were aligned with the phase-locked stimulation train, resulting in a significant increase in within-trial phase locking (WTPL) across all bands (Fig. 7F).

In contrast, arrhythmic stimulation produced a clear dissociation between rhythmicity modes. In sustained bands (alpha and beta2), arrhythmic stimulation reduced rhythmicity relative to baseline (blue clusters around the peak frequency (0); Fig. 7F), consistent with interference with ongoing oscillatory activity by irregular inputs. Conversely, in the transient band beta1, arrhythmic stimulation increased rhythmicity for up to 200 ms after the final pulse, likely reflecting the accumulation of transient responses, each causing a ripple containing a prominent beta1 component (see Fig. 7D). This double dissociation provides support for our hypothesis: that low-rhythmicity bands comprise transient yet spectrally defined input-driven activity, whereas high-rhythmicity bands support the maintenance of ongoing oscillations. Thus, the rhythmicity-resolved spectral architecture captures an important distinction in how the brain maintains versus responds to activity.

## Discussion

**Fig. 8:**
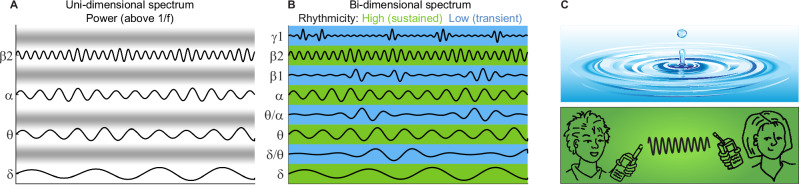
Rhythmicity reveals a bi-dimensional architecture. Brain rhythms can be classified along a new dimension of rhythmicity, revealing a universal spectral architecture: high-rhythmicity (sustained) bands maintain ongoing activity, while low-rhythmicity (bursty) bands denote the response to change. This framework generalises across species, sexes, recording techniques, and cognitive states. **A** Traditional view—the spectrum is divided to logarithmically progressing oscillatory bands. Role of activity expressed in the gaps between band (blurred grey borders) is unclear. **B** Proposed view—rhythmicity dimension distinguishes sustained (green) vs. bursty (blue) bands. **C** Functional division between the two rhythmicity modes. Bursty bands are associated with the processing of transient incoming inputs (metaphorized as ripples resulting from a drop of water). Sustained bands enable the maintenance of brain states and the putative orchestration of engaged brain areas (metaphorized as a telecommunication through sustained radio frequency, green). Artwork credit: “Water Drop Vector” © Allvectors.com. Available at https://www.freevector.com/water-ripples-vector, licensed under CC BY 4.0 (https://creativecommons.org/licenses/by/4.0/). Modifications: cropped and colour-adjusted for figure composition. Telecommunication contributed to the manuscript by Mirjam Karvat.

In this study we uncover a rhythmicity-resolved spectral architecture composed of two types of bands—sustained and transient—which alternate intermittently along a logarithmically scaled frequency axis. Rhythmicity (i.e., sustained and bursty) is revealed as a fundamental and previously overlooked dimension of brain dynamics (Fig. 8). This functional segmentation of spectral activity provides a principled basis for defining frequency bands, which is essential for aligning findings across studies (Fig. 5) in both humans and animals. Furthermore, rhythmicity-based spectral segmentation reveals twice as many bands as previously described with two divergent modes of operation: activity in transient bands indicates neuronal inputs, whereas activity in high rhythmicity bands signifies maintenance of neuronal activity via sustained oscillations. This duality can conceivably expand the capacity and efficacy of neuronal computations and, with a spectrum spanning several orders of magnitude^5^, can equip the brain with the flexibility necessary for a wide range of cognitive functions.

Segmenting the spectrum according to rhythmicity also opens up theoretical avenues for understanding how different rhythmic regimes can support distinct neural coding strategies. For example, the bi-dimensional organization of spectral phenomena may bridge two prominent views of the brain’s *modus operandi*, namely phase-coding^40^ and rate-coding^41^. These views differ regarding whether the precise timing of neuronal outputs encodes information about neuronal inputs. Phase-coding posits that oscillations temporally bias neuronal responses to inputs: synaptic inputs that arrive during the excitable phase of an oscillation are more likely to cause outputs; whereas inputs arriving at the opposite phase get dismissed or curtailed^42,43^. According to this view, oscillations causally influence the formation, synchronization, and sequence of cell assemblies, communication efficacy, and ultimately, an organism’s behaviour^40,44–46^. Within the rhythmic architecture framework, this suggests that sustained oscillations may maintain temporal scaffolds for coordinating neuronal responses. Much like radio signals rely on stable carrier frequencies to transmit information, sustained rhythms may facilitate local and distant communication by aligning excitability windows across neuronal populations and networks^47,48^.

In contrast, rate-coding proposes that the rate at which neurons fire contains all relevant information. According to one interpretation of rate-coding^41^, enhancing neuronal firing-rate suffices for assembly formation, deeming oscillations unnecessary. In the context of rhythmic architecture, previous studies have shown that surges in firing rate tend to align with troughs of field potentials during oscillatory bursts—initially observed in the gamma-^23,49^ and beta-bands^50^ and more recently across the entire 3–120 Hz spectrum^13^. Thus, even if oscillations are not required for information transfer per se, they serve as a valuable indicator of moments of heightened neuronal activity. Much like ripples on a calm water surface mark the impact of a falling object, oscillatory bursts—confined to low-rhythmicity frequency bands—act as spectral markers of cognitively relevant events.

Rhythmicity may thus provide the missing link between phase- and rate-coding schemes. In high-rhythmicity bands, oscillations are sustained and impose stable phase relationships that organize ongoing activity. In contrast, low-rhythmicity bands capture transient firing events driven by discrete inputs. By distinguishing between these two rhythmicity regimes, our framework reveals how the same neural system can flexibly alternate between temporally precise coordination and event-driven responsiveness—thereby integrating phase and rate coding within a single, rhythmically governed architecture.

An important advantage of introducing rhythmicity as an additional dimension is that it allows us to build upon, rather than discard, prior knowledge. We do not suggest that all transient bands, or all sustained bands serve the same function. There remains considerable scope for functional specialisation among specific bands. For example, within sustained rhythms, alpha activity is often associated with the maintenance of a widespread, general rest state^51^, whereas beta2 has been linked to keeping more targeted forms of cognitive control^52^.

Turning to transient bands, these may reflect inputs from distinct sources. For instance, evoked responses—often considered the frequency-domain analogue of time-domain event-related potentials—are typically dominant just below 10 Hz^53^, suggesting that the theta/alpha transient band may reflect broad, stimulus-driven synchronisation. In contrast, beta bursts have been associated with the spontaneous activation of neural networks, where coincident bursts across regions may indicate functional connectivity^54^. In this context, beta1 may be conceptualised as reflecting internally generated inputs, such as packets of action potentials sent from one brain region to its targets^55^. These examples highlight the potential of rhythmicity-based segmentation to support a more nuanced functional characterisation of the electrophysiological spectrum in future studies.

Our consistent observation that broad frequency bands can be subdivided into rhythmically distinct sub-bands may help clarify longstanding ambiguities about their functional roles. For instance, activity within the canonical beta band ( ~ 12–30 Hz) has been associated with both sustained and burst-like dynamics. When conceptualised as sustained oscillations, beta is thought to support the maintenance of ongoing motor or cognitive states—for example, continuous beta activity over sensorimotor cortex has been linked to tonic motor inhibition or postural maintenance^56^, and sustained beta synchronisation in frontoparietal networks has been associated with stabilising cognitive sets and top-down control, suggesting a role in signalling the cognitive status quo^52^. Conversely, when viewed as transient bursts, beta activity has been linked to event-related processes, such as brief motor cortex beta bursts marking movement termination^16^ or successful inhibition in stop-signal tasks^57^. Notably, one study^11^ demonstrated that both transient and sustained beta responses can co-occur within individuals, varying with task stage—transient responses following informative cues, and sustained responses during anticipatory periods. These were also spectrally distinct, occupying different sub-bands within the broader beta range. It is therefore plausible that previously reported, and at times seemingly contradictory, functional roles attributed to beta activity may in fact reflect spectral segregation into low- and high-rhythmicity sub-bands within the band referred to as beta.

A further promising direction for future research concerns the interactions between neighbouring frequency bands. We observed that high-rhythmicity bands can attenuate rhythmicity in adjacent frequency ranges via phase interference at the level of the measured signal (Fig. 3A; see Fig. S5). Whether such interactions reflect cellular-level mechanisms will require future intracellular recordings and is beyond the scope of the present study. At the level of the measured signal, ongoing oscillatory activity in a dominant high-rhythmicity band may curtail the development of nearby rhythms through phase-based interactions (i.e., interference). This interpretation is supported by our finding that removing the high-rhythmicity band using a stop-band filter abolished the observed phase shift (Fig. 3A), consistent with LAVI’s sensitivity to phase differences. This interpretation, together with the dominance of high-rhythmicity events (higher power even when normalized to compensate the 1/*f* slope, Fig. 3F) also carries intriguing implications for the broader operational principles of the brain. It raises the possibility that high-rhythmicity bands may serve to establish dedicated “quiet” spectral zones—frequency ranges held in readiness for incoming input. In such a scenario, an external or internal input would produce a brief, ephemeral response, which is then rapidly curtailed by the surrounding rhythmic context. This mechanism would not only support a top-down role for sustained oscillations but also suggest that such control is not purely inhibitory. Rather, it may enhance contrast and selectivity by dynamically shaping the spectral landscape to prioritise response-relevant signals.

While our findings offer compelling and consistent insights into the functional architecture of brain rhythms, it is important to acknowledge certain limitations that may inform future refinements and interpretations. First, for practical reasons, we have chosen to focus on the 3–45 Hz range. This choice limited our ability to characterise dynamics in both lower (notably δ and δ/θ) and higher (γ and higher frequencies) frequency bands (Fig. 4). Nonetheless, both LAVI and ABBA are applicable to these ranges, provided that specific considerations are taken into account. For low frequencies, LAVI’s noise levels are influenced by session duration (Fig. S6); thus, for frequencies below 3 Hz, we recommend using recordings of at least several minutes. Conversely, high-frequency LAVI is sensitive to the sampling rate, and we advise a minimum sampling frequency of 1 kHz. Additionally, power line noise (50/60 Hz) is highly rhythmic and can substantially affect frequencies above ~40 Hz. Strong notch filters are not recommended, as they distort the rhythmicity of neighbouring frequencies (Fig. 3I, blue traces). Instead, we suggest using approaches such as spectral interpolation^58^, which minimise distortion in the time domain while effectively reducing line noise.

Second, while our TMS findings provide causal support for the proposed spectral architecture, we acknowledge that neurostimulation studies inherently involve greater variability and interpretative complexity than observational analyses. Although the current protocol yielded informative results consistent with previous reports of spectrally confined responses to single-pulse TMS^39^, it was constrained by factors such as sample size and the absence of behavioural correlates. Future studies could strengthen these findings by incorporating larger and more diverse cohorts, as well as concurrent behavioural or task-based measures to directly link rhythmicity modulation with cognitive function. Such refinements would enable a more precise characterisation of how rhythmic and arrhythmic inputs interact with distinct spectral modes and their functional relevance.

Despite these methodological limitations the present study points toward exciting future directions for investigating the basis and function of neural rhythmicity. The origins of neural oscillations—though extensively explored in both experimental and computational domains—can now be explored through the prism of distinct rhythmicity modes. A promising direction for future research lies therefore in exploring how the rhythmic architecture described here might reshape our understanding of the anatomical and computational bases of oscillatory activity. For instance, could certain anatomical structures be more conducive to one rhythmicity mode over another^19^ ? Would the characteristics of the rhythmic architecture (e.g. peak frequencies and band ranges) change systematically with anatomical location^19,59,60^ ? Might distinct mechanisms underlie the generation of oscillations in the two modes—such as PING (pyramidal-interneuron gamma) circuits for bursty activity^61^, versus pacemaker cells for sustained rhythms^62^ ? (For a comprehensive review, see ref. ^63^.) Furthermore, identifying the computational principles that best account for these two modes of operation—and understanding their functional and cognitive consequences—represents an exciting avenue for future investigation.

In conclusion, our findings establish rhythmicity as a fundamental dimension of brain dynamics, revealing a dual sustained–bursty spectral architecture that underpins flexible and efficient neural computation. This rhythmicity-resolved framework generalises across species, sexes, brain regions, recording modalities, and cognitive contexts. Its theoretical and practical implications are considerable. Theoretically, it provides a principled analytic foundation for aligning physiological findings across humans and animals, facilitating the translation of mechanistic neuroscience into human application. Practically, it opens new avenues for precision neuroscience—enabling individualised and statistically robust spectral segmentation to inform brain stimulation protocols, advance non-invasive diagnostics for neuropathology, and guide the design of next-generation brain–computer interfaces.

## Methods

### Participants and data acquisition

We sought to establish the rhythmicity architecture as a universal phenomenon; therefore, we analysed datasets from different laboratories and recording techniques (Table 1). Participant sex was recorded in the original datasets where available. A dedicated analysis of sex differences was performed in Dataset VIII (see Results, Fig. 5E–F), revealing strong evidence for no sex-based differences (BF_01_ = 43.25). Consequently, data were pooled across sexes in subsequent analyses. Sex was determined based on the procedures reported in the original studies. For datasets where sex information was not available, analyses were conducted on the full sample as provided.

#### Datasets I-III

The original datasets comprised 45 participants in Datasets I–II and 31 participants in Dataset III. Data from eight participants in Datasets I–II were excluded due to technical issues (poor or missing EEG signal), and data from two participants in Dataset III were excluded due to technical issues (poor signal quality or missing triggers). The final samples comprised 37 participants in Datasets I–II (27 women, 10 men; age = 22.9 ± 2.6 years, mean ± SD) and 29 participants in Dataset III (16 women, 13 men; age = 24 ± 3.1 years, mean ± SD). Sex was determined based on participant self-report in the original studies. Participants were recruited from a university population. All procedures were approved by the institutional review board of the Hebrew University of Jerusalem, and participants provided informed consent. Participants were compensated either monetarily (10 € per hour) or with course credit.

#### Dataset IV

The sample comprised 29 participants (17 women, 12 men; age range = 18–42 years). Data from two participants were excluded due to excessive EEG noise and replaced to maintain sample size in the original study. Sex was determined based on participant self-report in the original study. All procedures were approved by the ethics committee of Kobe University, Japan, and participants provided informed consent.

#### Dataset V

The original dataset comprised 36 participants. In the original study, data from four participants were excluded, resulting in a sample of 32 participants (20 women, 12 men; mean age = 25 years, range = 20–34 years). In the present study, data from nine participants were excluded due to excessive EEG artefacts or poor data quality, yielding a final sample of 27 participants. Sex was determined based on participant self-report in the original study. All procedures were approved by the regional ethics committee at Lund University, and participants provided written informed consent. Participants were compensated with two cinema tickets.

#### Dataset VI

The sample comprised 24 participants (age range = 18–35 years). Sex information was not reported in the original study and could not be reconstructed from the available data. All procedures were conducted in accordance with institutional guidelines, and participants provided informed consent.

#### Dataset VII

Twelve participants were recruited via internet-based advertisements at the MRC Cognition and Brain Sciences Unit SONA participant pool. Six participants withdrew due to discomfort during frontal rTMS. The final sample comprised six participants (four women, two men; age = 27.1 ± 7.1 years, mean ± SD). Sex was determined based on participant self-report. All procedures were approved by the Cambridge Psychology Research Ethics Committee, and participants provided written informed consent prior to participation. Participants were compensated £12 per hour.

#### Dataset VIII

MEG data were drawn from Stage 2 of the Cambridge Centre for Ageing and Neuroscience (Cam-CAN; www.cam-can.org^64^) study, a population-based adult lifespan cohort (18–88 years). Ethical approval was obtained from the East of England–Cambridge Central Research Ethics Committee, and participants provided written informed consent. Participants were recruited from the general population via primary care registers. The present study included 625 participants (317 women, 308 men) with resting-state MEG data suitable for analysis following motion correction. Sex was recorded in the original dataset based on participant self-report and was approximately balanced across the lifespan sample. Sex differences were explicitly examined in the present study (see Results, Fig. 5E–F), revealing strong evidence for no sex-based differences. Further details on inclusion and exclusion criteria are provided in the original report (see Table 1 in ref. ^64^).

#### Dataset IX

Intracranial electrophysiological (ECoG) data were obtained from 10 patients (4 women, 6 men; age = 41.0 ± 12.2 years, mean ± SD) with drug-resistant epilepsy undergoing clinical monitoring with subdural electrodes. Electrode implantation sites were determined solely based on clinical considerations. Participants were recruited from clinical centres (Stanford School of Medicine, California Pacific Medical Center, and UCSF Medical Center). All participants provided written informed consent, and all procedures were approved by the UC Berkeley Committee on Human Research and the corresponding institutional review boards at the clinical recording sites.

#### Dataset X

Simultaneous scalp EEG and stereoelectroencephalography (SEEG) data were obtained from 12 patients (age = 27.5 ± 6.3 years, mean ± SD) with drug-resistant epilepsy undergoing pre-surgical clinical evaluation. Sex information was not reported in the original study and could not be reconstructed from the available data. Electrode implantation and placement were determined solely based on clinical considerations. Participants were recruited from the Emergency University Hospital Bucharest. All participants (or their legal guardian/next of kin) provided written informed consent, and all procedures were approved by the University of Bucharest Ethics Committee for Research.

#### Dataset XI

Local field potential (LFP) recordings were obtained from 17 patients (4 women, 13 men; age = 56.4 ± 6.7 years, mean ± SD) with Parkinson’s disease undergoing deep brain stimulation of the subthalamic nucleus. Recordings were performed postoperatively while electrode leads were externalised, and electrode placement was determined solely based on clinical considerations. Participants were recruited at St George’s University Hospital and King’s College Hospital (London, UK), and the University Medical Center Mainz (Germany). All participants provided written informed consent, and all procedures were approved by the relevant local ethics committees.

#### Dataset XII

LFP and single-unit recordings were obtained from 11 male Long-Evans rats (250–400 g). Animals were implanted with multisite silicon probes targeting the medial entorhinal cortex and hippocampus and recorded during freely behaving spatial tasks. All experimental procedures were approved by the Institutional Animal Care and Use Committee of Rutgers University.

### EEG acquisition and preprocessing

We performed all offline preprocessing and analysis steps using the Fieldtrip toolbox, release 20230822^65^ and custom code in Matlab 2020b (MathWorks, Natick, MA). In datasets I-III^22,66^, data were sampled at 512 Hz using a g.GAMMAcap (gTec, Schiedlberg, Austria) and a g.HIamp amplifier (gTec). The cap had 62 electrodes distributed over the scalp, with the addition of two active earlobe electrodes. All electrodes were re-referenced offline to the average of the earlobe electrodes. In dataset IV^67^, data were sampled at 2048 Hz using an ActiveTwo EEG system (Biosemi, Amsterdam, Netherlands). The cap had 32 electrodes that were re-referenced offline to the average of two electrodes over the temporal lobe (T7 and T8, as recommended for channel level analysis when earlobe electrodes are unavailable, see https://www.fieldtriptoolbox.org/getting_started/biosemi/). Data in dataset V^68^ was recorded with a Neuroscan (Compumedics, El Paso, TX, USA) NuAmps amplifier, with 30 channels sampled at 1000 Hz and re-referenced to electrodes over he left and the right mastoid. In dataset VI^69^, data were acquired from 128 channels using EGI Hydrocel Geodesic Sensor Net (HGSN-128, Magstim EGI, Eugene, OR), sampled at 1000 Hz, and re-referenced offline to the average of all channels.

All succeeding preprocessing procedures were identical for all EEG datasets. We detrended, demeaned and bandpass filtered the data at 0.5–130 Hz. Unless stated otherwise, all filtering was performed using the FieldTrip function ft_preprocessing with default settings (4th-order Butterworth IIR filter, applied bidirectionally using zero-phase filtering). Then, we inspected the data visually and rejected bad channels, inserting interpolation of neighbouring channels instead of the bad channels. On average, we rejected 8.54% of electrodes from dataset I (SD 1.65%), 0.04% (0.26%) from dataset II, 2.3% (3.2%) from dataset III, 1.17% (2.22%) from dataset IV, 4.5% (1.9%) from dataset V, and 5.9% (5.0%) from dataset VI. Importantly, channel Cz, which was used for rhythmicity and oscillatory bursts analyses, was good for all 178 EEG subjects. Scalp muscle artefacts were detected and marked as epochs in which the amplitude of the signal at 100–120 Hz at all channels exceeded a threshold of 30 standard deviations. Finally, independent spatio-temporal components containing eye movements (blinks and saccades) were removed using independent component analysis (ICA). We preformed all preprocessing steps, as well as rhythmicity (LAVI), power, and oscillatory bursts analyses, on the whole session (that is, without dividing into trials).

For ECoG (dataset IX) we used all 907 channels reported “clean” in the original publication^70,71^. In the per-area analysis (Fig. 4D-F) we excluded electrodes reported as “Medial” due to low count (*n* = 12), or those that were marked as depth electrodes, and therefore lacked a cortical area assignment (*n* = 28). After exclusion, this analysis consisted of 867 electrodes. For SEEG (dataset X^72^) we used 733 electrodes reported as located in mesial-frontal, lateral-frontal, insula, medial-parietal, lateral-parietal, lateral-temporal, or mesial-temporal areas. In addition, for each area, we included the nearest concurrently recorded EEG scalp electrode. For LFP recordings, we used one STN channel from each hemisphere On and Off PD medication in dataset XI^73^ sampled at 2048 Hz using a TMSi Porti (TMS International, Netherlands) or from the hippocampus of each rat in dataset XII^30^.

### MEG acquisition and preprocessing

MEG data were collected using a 306-channel VectorView MEG system (Elekta Neuromag, Helsinki), consisting of 102 magnetometers and 204 orthogonal planar gradiometers, located in a magnetically shielded room. MEG resting state data (sampled at 1 kHz with a high-pass filter of 0.03 Hz) were recorded approximately 8.5 minutes, while participants remained still in a seated position with their eyes closed, but instructed to stay awake. Head position within the MEG helmet was estimated continuously using four Head-Position Indicator (HPI) coils to allow offline correction of head motion.

The MaxFilter 2.2.12 software (Elekta Neuromag Oy, Helsinki, Finland) was used to apply temporal signal space separation^74^ to the continuous MEG data to remove noise from external sources (correlation threshold 0.98, 10-sec sliding window), to continuously correct for head-motion (in 200-ms time windows), to remove mains-frequency noise (50-Hz notch filter), and to detect and reconstruct noisy channels. Following these de-noising steps, data were imported into Matlab using SPM12 (http://www.fil.ion.ucl.ac.uk/spm). These data are available on request from https://cam-can.mrc-cbu.cam.ac.uk/dataset/.

The first 20 seconds of data were ignored to allow participants to settle, and any samples after 542 seconds were ignored, in order to match data length across participants (to the minimum duration across participants). These data were then extracted from 4 magnetometers around Cz.

### Lagged angle vector index (LAVI)

We measured rhythmicity as the consistency of the relations of phase (angle) between data points separated by a fixed number of cycles (“lag”, with the corresponding duration in ms determined by each frequency, and see Fig. 1 for considerations for choosing lag duration). The rational for defining rhythmicity in this manner is that the phase of a sustained oscillation at any time-point should predict its future phase^18^.

We computed the time-frequency representation $\hat{x}$ for each time-point *t* and frequency of interest *f* using complex Morlet wavelets, of the form

1 $$w\left(t,f\right)={Ae}^{\left(\frac{-2{\left(\pi {ft}\right)}^{2}}{{m}^{2}}\right)}{e}^{\left(2i\pi {ft}\right)\,}$$

Where *i* is the imaginary unit and *m* is the wavelet width, measured in cycles. The normalization factor was$A=\sqrt{2f\sqrt{\pi }/m}$. The value of *m* was set to 5, based on the exploration presented in Fig. 1E and Fig. S2D. Care was taken to produce angles with the following convention: cosine must always be 1 and sine must always be cantered in upgoing flank, so the centre of the wavelet has the angle of 0 rad^65^. The time-frequency representation was computed by convolving the signal with the Morlet wavelets at each frequency. For computational efficiency, this was implemented in the frequency domain by multiplying the Fourier transform of the signal with the Fourier-transformed wavelets, and applying an inverse Fourier transform to obtain the time-varying complex spectra at each frequency. Then, the rhythmicity λ was measured for each frequency as the magnitude of the vector mean of all angle differences (“coherence”) between the original time-frequency representation ${\hat{x}}_{(t,f)}$ and a copy of itself with a constant lag of τ cycles ${\hat{x}}_{(t+{L}_{(f)},f)}$:

2 $$\lambda \left(f\right)=\left|\frac{{\sum }_{t=1}^{T-{L}_{(f)}}{\hat{x}}_{(t,f)}{\hat{x}}_{(t+{L}_{(f)},f)}^{{\prime} }}{\sqrt{\!\!\left({\sum }_{t=1}^{T-{L}_{(f)}}{|{\hat{x}}_{(t,f)}|}^{2}\right)\left({\sum }_{t=1}^{T-{L}_{(f)}}{|{\hat{x}}_{(t+{L}_{(f)},f)}|}^{2}\right)}}\right|$$

Where *T* is total number of time points in the session, *L*_*(f)*_ is the number of time points in τ cycles and the superscript ${\prime}$ denotes the complex conjugate transpose. By definition, $\lambda$ assumes values between 0 and 1. The median of $\lambda$ (over *f*) is stable across subjects and depends on *m* and τ. Based on the exploration presented in Figs. 1D and  S2D, we set τ to 1.5 cycles, which together with *m* = 5 cycles yields a median λ of ~0.4, providing a good dynamic range (for further discussion, see Supplementary Text, Parametrical effects on rhythmicity measures).

Automated Band Border Algorithm (ABBA)

Using the LAVI median as a threshold, one can define areas above the median as “sustained” bands, and areas below as “transient” bands. However, fluctuations above and below the median are present also in the rhythmicity profiles of purely random signals (noise). The median of the rhythmicity profiles of noise also depends of *m* and τ, and their dispersion range depends on the sampling frequency, session duration, and the aperiodic slope (for further discussion, see Supplementary Text, parametrical effects on rhythmicity, and Fig. S6). This relationship can be leveraged to determine spectral bands with statistical confidence using a bootstrap approach: frequencies whose rhythmicity levels exceed (or fall below) the top or bottom k observations from n bootstrap samples of noise can be considered sustained (or transient), corresponding to a two-tailed Type I error rate of α = k/n. In our analysis, we generated *n* = 200 surrogate noise samples and identified frequencies as sustained or transient if their rhythmicity levels fell within the top or bottom 2.5% (k = 5) of the bootstrap distribution (α = 0.05).

To generate the distribution of rhythmicity profiles we generated surrogates that matched the aperiodic component (1/*f*) of each subject. We calculated the aperiodic component of the EEG by fitting an exponential function to the EEG power spectrum, and used the fitted values as the input for an Iterative Amplitude Adjusted Fourier Transform algorithm (IAAFT^75–77^). IAAFT generates surrogate time series with a desired power spectrum (in the frequency domain) and data values (in the time domain) by keeping the original magnitude of the Fourier coefficients and assigning random phases. In each iteration of the algorithm there are five steps: a. initializing with a randomly shuffled version of the original time series to destroy temporal correlations while preserving the amplitude distribution; b. computing the Fourier transform of the shuffled time-series; c. replacing the magnitude of the Fourier coefficients with the desired magnitudes (i.e., those of the original time series) while keeping the current (random) phase; d. computing the inverse Fourier transform to obtain a new time series with the desired spectral properties, and e. matching the amplitude distribution: this is done pointwise by ranking the new time series and replacing its values with those of the original series sorted by rank (e.g., the highest value in the new series receives the highest value of the original series, the second-highest receives the second-highest, etc.). Since step (e) alters the spectral properties, this algorithm is repeated iteratively until the error between the desired and current spectra falls below a defined threshold (here, 2 × 10⁻⁴ of the original standard deviation).

The IAAFT implementation for Matlab^78^ can take the original EEG values as input, but since noise levels can be predicted from experimental parameters (Supplementary Text—Parametrical Effects on Rhythmicity, and Fig. S6) and in order to save computational time and facilitate the computation of hundreds of channels, we generated surrogate distributions of 200 repetitions with different sampling frequencies, durations, and aperiodic exponents. This resulted in a look-up table that was used to define significance limits for each frequency and channel with α = 0.05. Then, ABBA defines each frequency with a LAVI value above (below) the significance limit as significantly sustained (transient). Frequencies between points of crossing the median are then grouped into bands. The local maximum is taken as the peak frequency of sustained bands, and the local minimum is the peak frequency of transient bands. Since most subjects have a clear peak in the rhythmicity profile at the alpha band, the peak between 6 to 14 Hz can be used as an anchor to automatically allocate bands identity: the frequency with highest rhythmicity between 6 and 14 Hz is taken as alpha, the trough following alpha, as the frequency increases (i.e., to the right of alpha) is beta1, the next peak is beta2, and so forth, and also for frequencies lower than alpha. A Matlab implementation for LAVI and ABBA (including pink surrogate and look-up table generation) is available at https://github.com/laaanchic/LAVI^79^. This code is using functions provided by Fieldtrip^65^ and Venema V^78^, and is shared under the GNU General Public License (GPL) and Berkeley Software Distribution (BSD) license, respectively.

In Fig. S2 we compare band-detection with LAVI and ABBA to a commonly used power-based method. To this end, we computed the power spectrum of the EEG of 90 participants (using the Matlab function ‘pwelch’), and used Spectral parameterization (specparam, formerly fooof^8^) with recommended default configurations to detect the range and peak frequency of bands in the frequency range 2–40 Hz.

### Simulations

For all simulations presented in Figs. 3, S1, S4, and S5, we generated 180 s “pink” surrogate data using IAAFT, with aperiodic exponent of -1, range of ~100 μV, mean 0 μV and SD ~ 12.5 μV. To manipulate the activity in a specific frequency band, we first decomposed the original signal using an array of third-order Butterworth bandpass filters (centred at 3–100 Hz in 1-Hz steps, each with a 1-Hz bandwidth). This decomposition was performed once and served as a common basis for all subsequent manipulations. In each manipulation iteration, we selectively modified the signal in the 15-Hz band and then reconstructed the full signal by summing all band-limited components. Finally, we computed and stored the rhythmicity profile (LAVI) of the reconstructed signal using the same parameters applied throughout the manuscript (wavelet width of 5 cycles and a lag of 1.5 cycles).

To simulate bursts of varying durations in an otherwise quiet band (Fig. 3H-I), we first attenuated the signal filtered at 14–16 Hz by multiplying it by 0.5. We then introduced bursts by multiplying segments of the signal by 2, with a smooth rise and fall. Burst duration was the independent variable, ranging logarithmically from 2 to 12 cycles, while the inter-burst interval was jittered between 5 and 15 cycles.

To simulate bursts with varying degrees of frequency drift (Fig. S4A–B), we defined each burst as a sinusoid of the form y = sin(2π *f t*), where the instantaneous frequency *f* linearly drifted from 15+drift/2 to 15-drift/2 Hz. The slope of this drift was the dependent variable and ranged logarithmically from 0.1 to 10 Hz.

In the transient input simulations (Fig. S4C-F), the initial conditions were identical to the oscillatory burst duration simulation. Each transient input was simulated as an impulse (one sample long) with amplitude equal to the EEG range (100 μV). The number of impulses in a “burst” was the independent variable and ranged linearly from 1 to 13. In the rhythmic condition (Fig. S4C-D), the distance between each impulse in a “burst” was set to 66.6 ms (one cycle of a 15 Hz oscillation). In the arrhythmic condition (Fig. S4E-F), the last impulse in a “burst” was set to 66.6*(*n*-1) ms after the first pulse, with *n* the number of impulses in a “burst”. Then, the remaining *n*−2 impulses were randomly distributed between the first impulse and last impulse. Like in the burst duration simulation, the inter “burst” interval jittered between 5 and 15 cycles.

To implement phase shifts (Fig. S1), we flipped the sign of the signal by multiplying it by −1 every 2 to 20 cycles (in logarithmic steps). To investigate the effect of power on rhythmicity (Fig. S5A-B), we multiplied the data filtered at 15 Hz by values ranging between 0.1 and 2, in steps of 0.1. In all simulations, we defined the significance limits as the maximum and minimum rhythmicity values of the original surrogate data, and the main dependent variables were the rhythmicity values at the manipulated frequency (15 Hz). All rhythmicity calculations were made with Wavelet width of 5 cycles and lag of 1.5 cycles.

As can be observed in Figs. 3I,  S1B, and  S5B, increasing (decreasing) the rhythmicity in one band decreases (increases) rhythmicity in neighbouring bands. To investigate if the troughs in the rhythmicity profiles we observed in the data of 90 subjects can be explained as an (artefactual) effect of the peak in alpha, we generated 90 instantiations of 120 s 1/*f* surrogates. We decomposed the surrogates into discrete frequencies, and multiplied the amplitude of the signal in 11 Hz (alpha) by a factor randomly chosen between 1.05 and 2.25. We then calculated the rhythmicity profiles (LAVI) and detected bands (ABBA). Then, we calculated the fit between the LAVI values at alpha peak and beta1 trough of the surrogate population with a linear regression. We used the coefficients of the regression to calculate the expected beta1 troughs based on data alpha peaks, and compared them to beta1 troughs observed in data (Fig. S5C-D). Finally, we compared the LAVI values at theta/ alpha and beta1 troughs of the data to the troughs of surrogates generated with aperiodic components and power in alpha matching the observed data, using filter arrays (Fig. S5E).

### Bursts analysis

To detect oscillatory bursts, we first estimated the power of the raw EEG using Morlet wavelets with a width of 5 cycles, centred at frequencies spanning logarithmically from 3 to 45 Hz. Artefactual segments—defined as time points where the EEG exceeded 250 μV or intervals marked as muscle artefacts during preprocessing—were removed, along with a 500 ms buffer before and after each segment^80^. Burst peak time and frequency were identified as local maxima in the two-dimensional time–frequency plane that exceeded the 90th percentile of power. We then recorded the peak frequency and its corresponding time point.

To align individual bursts to a common reference point and compute the burst-related potential (BRP; Fig. 3A), we band-pass filtered the raw signal using the band borders defined by the LAVI and ABBA methods as the filter’s corner frequencies. We then identified the nearest trough in the filtered signal and defined it as time zero (t = 0), extracting an epoch of 3 cycles before to 3 cycles after this point. To assess potential inter-band influences, we also extracted data at the time of each burst while suppressing activity in neighbouring bands (with notch filters).

Next, we determined the burst onset and offset in two steps. First, we defined the initial burst boundaries as the time points at which power at the peak frequency dropped below the 75th percentile. Next, we identified all frequencies that peaked at any time point within this initial window. The final burst onset and offset were then defined as the time points at which all of these peak frequencies fell below the 75th percentile of either power (Fig. 3B) or rhythmicity (WTPL, Fig. 3C).

Since we were interested in average durations and rates of bursts in the different bands over the whole session, we paid special attention to avoiding bursts overlapping in time/frequency. Therefore, if two bursts overlapped in time, and also had a peak frequencies difference smaller than a quarter of the peak frequency of either burst, they were merged into one burst. The merged burst was assigned the frequency of the burst with the higher energy (estimated as power x duration).

Then, to calculate the burst rate (Fig. 3D), we counted the total number of bursts in each band across the entire session and divided by the session duration and band width. This latter normalisation accounts for the fact that the band width increases logarithmically with frequency.

Occupancy (Fig. 3E) was calculated by summing up all time points between the beginning and end of each burst in each band, dividing by the total number of samples, and multiplying by 100 to obtain percentages.

Relative power (Fig. 3F) was defined as the dB value (10 × log_10_) of the ratio between the peak-power and the 90th power percentile of the peak-frequency.

Finally, band consistency (Fig. 3G) was defined, for each burst, as the number of time-points in which the peak-frequency was confined within the LAVI-defined frequency band of the burst itself, divided by the total number of samples in the burst, and multiplied by 100 for percentage representation.

### TMS- procedure

Biphasic single and repetitive TMS pulses were delivered using a DuoMAG XT−100 TMS stimulator and a figure-of-eight coil DuoMAG 70BF (Brainbox Ltd, Cardiff, UK). During the stimulation, participants sat in a comfortable recliner chair with a neck rest. To ensure the precise targeting of specific brain regions, the coil was controlled via Brainsight 2 neuronavigation system (Brainbox) in combination with an Axilum TMS-Cobot (Axilum Robotics, Schiltigheim, France). The Cobot is a robotic system that actively monitors and adjusts the positioning of the coil, and compensates for head movements throughout the experiment. To detect head movements, participants wore a headband reference tracker that was monitored by a Polaris Vega ST camera (NDI, Waterloo, Canada). The TMS coil was oriented with the handle pointing posteriorly with respect to the participant’s head, at an angle of 45 degrees relative to midline. The MNI coordinates for dorso-lateral prefrontal cortex (dlPFC, x = 33, y = 39, z = 26) stimulation were derived from the peak voxel showing the strongest effect in BOLD signal in a previous meta-analysis study^81^.

Our protocol established a TMS stimulation intensity at 90% of the resting motor threshold (RMT) of each participant. To determine the RMT, we positioned the coil over the hand area of the right primary motor cortex and asked the participant to keep their left hand relaxed and at rest. Then, we determined the minimum intensity at which a single TMS pulse produced a visible twitch in the abductor pollicis brevis muscle of their left hand, in five of ten successive pulses.

EEG recordings were obtained with the actiCHamp Plus 64 system (Brain Products GmbH, Gilching, Germany), which is TMS-compatible. The system includes a DC-coupled amplifier avoiding AC recoding and high-pass filter during the recording period. EEG signals were acquired from 64 active electrodes arranged on an actiCAP slim electrode cap. The ground electrode was placed at FPz, and the reference at Cz. Electrode impedance was maintained below 20 kOhm. We used a sampling rate of 1000 Hz for the EEG resting-state recording and 5 kHz for the TMS-EEG recordings.

After EEG setup, we used LAVI and ABBA to determine the individual alpha, beta1, and beta2 peak frequencies that would define the repetitive TMS stimulation frequencies. For this, we performed a resting-state EEG recording of 12 minutes (2-minutes with open eyes, 8-minutes closed eyes, and 2-minutes open eyes). During the fixation periods, participants were instructed to keep their eyes still and look at a white fixation cross presented on a black background. LAVI was calculated over the closed-eyes period.

The TMS-EEG session comprised a total of 480 trials distributed into 8 blocks. There were 7 conditions: 1 single pulse (sp-TMS), 3 rhythmic conditions consisting of a 6-pulses train at alpha, beta1 or beta2 individual frequencies, and 3 arrhythmic conditions, also consisting of 6-pulses train. For each arrhythmic condition we precomputed patterns of 6-pulses that excluded frequencies within a +/−2-Hz band centred on the frequency of the corresponding rhythmic condition, their harmonics, subharmonics and over 50 Hz. The length of both rhythmic stimuli was equal to 5 cycles of the peak frequency. Each block included 12 trials of the single pulse condition and 8 trials of each of the rhythmic and arrhythmic conditions, presented sequentially in a pseudorandomized order. During each block, participants were instructed to keep their eyes still and look at a white fixation cross presented on a black background. The inter-train interval (ITI) between two consecutive trials was adjusted to the preceding frequency according to safety guidelines^82^. For frequencies up to 10 Hz, we used a 3-s ITI; for frequencies over 10 Hz and up to 15 Hz, we used a 5-s ITI; for frequencies over 15 Hz and up to 20 Hz, we used an 8-s ITI; for frequencies over 20 Hz and up to 25 Hz, we used a 10-s ITI. Stimulus and TMS pulses delivery was controlled using Psychtoolbox-3^83^ in Matlab.

### TMS- data analysis

EEG data were pre-processed offline in Matlab using Fieldtrip and custom-written code, following previously published guidelines^84,85^. First, we removed the TMS ringing artefacts by replacing data values from 1 ms before to 15 ms after the TMS pulse trigger with NaNs (Not-a-Number). Then, we down-sampled the data to 1000 Hz. Afterwards, we visually inspected the data and marked fragments with major artefacts (e.g., jumps, movement, muscle); these fragments were also replaced by NaNs. Then, all the fragments with NaNs were interpolated with a linear method. To detect remaining TMS-related artefacts, we ran a first independent component analysis (ICA) with the FastICA algorithm. We averaged the components’ signal 50 ms after TMS pulse over all trials and rejected those components whose amplitude exceeded 30 μV. Then, we ran a second ICA. In this run, components relating to eye blinks, eye movements, muscle and sensor-localized noise were identified and removed. All bad data fragments were replaced via Piecewise Cubic Hermite Interpolation (pchip). Then, the Cz reference channel was recovered and the data was re-referenced to a common average reference. Finally, the data were cut into segments of each trial type, including a pre-train onset interval of 2.5 sec and post-train offset interval of 2 sec.

### Time-resolved rhythmicity

To measure rhythmicity-based burst-durations (Fig. 3C) and how rhythmicity changes in time within a trial in response to TMS pulses (Fig. 7D, 7F), we used the Within Trial Phase Lock (WTPL) method. This method is described in detail elsewhere^22^. In brief, WTPL measures time-resolved rhythmicity by asking how sustained the oscillation is for at least one period. Like LAVI, WTPL assumes that sustained oscillations are repetitive, and as such, the phase at any time point is expected to predict future and past phases. Specifically, the phase one cycle before or after a time point should be near zero, whereas phase slips cause deviations of this phase relation away from zero. In each frequency of interest, time-point, and trial, we computed the difference between the phase in the time-point of interest (*ϕ*_0_), one cycle beforehand (*ϕ*_−1_) and one cycle after (*ϕ*_1_).

The WTPL is computed as the mean resultant vector length of the two phase relations:

3 $${{\rm{WTPL}}}\left(f,t,n\right)=\frac{1}{2}|{e}^{i\left({\phi }_{0}-{\phi }_{1}\right)}+{e}^{i\left({\phi }_{0}-{\phi }_{-1}\right)}|$$

Where *f* stands for frequency, *t* for time point, and *n* for trial. A Matlab implementation for WTPL is available at https://github.com/laaanchic/WTPL^79^.

Note that since WTPL baseline levels of certain frequencies are higher than others (as learned from LAVI), between-frequency analysis is done after baseline subtraction (termed ΔWTPL in Fig. 7). We took the duration –1 s to –0.5 s (relative to the first TMS impulse) as the baseline. Note, that the TMS frequency used for each participant was dependent on their individual band peak frequency, which varied across participants. Therefore, in Fig. 7, we plotted the population-averaged ΔWTPL values after normalizing individual frequencies (that is, setting the peak frequency per band to “0”).

### Statistical tests

All statistical tests were performed using Matlab with the Statistics and Machine Learning toolboxes. Data throughout this manuscript are presented as mean ± *SEM* unless otherwise stated. Significance level was set at α = 0.05. If multiple tests were performed (e.g., different measures in Fig. 3), their *p*-values were corrected using the false discovery rate according to the Benjamini-Hochberg algorithm^86^. For post-hoc tests following ANOVA, we used the Fisher’s least significant difference procedure. All statistical tests are two-sided. Complete details of all statistical tests are presented in Table S1.

To define bands with statistically significant increased or decreased rhythmicity we adopted a bootstrap approach using a look-up table with α = 0.05 (see above, [Automated Band Border Algorithm (ABBA)]).

To test for differences in band-peak distributions between datasets or areas (Fig. 4), as well as for differences in burst characteristics between bands (Fig. 3), we used a one-way ANOVA (Matlab function “anova1”). In Fig. 3 we excluded participants with > 3 SD, resulting in the exclusion of 1 participant.

To assess whether two or more distributions differed, we computed the likelihood of the data under both the null and alternative hypotheses using Bayes Factors, which quantify the relative evidence for or against a difference. We used Bayes Factor (BF) *t*-tests or ANOVA, using a BF toolbox for Matlab^87^. The output of the bf.ttest or bf.anova Matlab function is BF_10_, or how strong is the evidence to support H_1_ (the distributions are different) and reject H_0_ (the distribution are not different). Its counterpart BF_01_ = 1/BF_10_ estimated how strong is the evidence to support H_0_ and reject H_1_. To determine the strength of this evidence we followed the guidelines suggested in ref. ^88^: 3 < BF_10_ < 10 indicates a moderate evidence for H_1_, and BF_10_ > 10 indicates a strong evidence for H_1_. Similarly, 1/10 < BF_10_ < 1/3 (or 3 < BF_01_ < 10) indicates a moderate evidence for H_0_, and BF_10_ < 1/10 (or BF_01_ > 10) indicates a strong evidence for H_0_. Default Cauchy priors for effect sizes were used (scale parameter = 0.707). No Markov-chain Monte-Carlo sampling was required, as Bayes factors were calculated analytically.

For comparing LAVI values across frequencies (Figs. 2D and 6C) we adopted a cluster-permutation approach^89,90^. This non-parametric method is appropriate to control the family-wise error rate since values of neighbouring frequencies are not independent from each other. We normalized the values of each subject by subtracting the median across frequencies. Then, we computed the *t* test at each frequency. After computing *t* values, we clustered neighbouring points exceeding a significance level of α = .05 and summed the *t* values of each cluster. This sum served as the value for comparison in the cluster-level statistics. We then created 1000 permutations of data with shuffled labels (i.e., eyes open or closed in Fig. 2D or medication On or Off in Fig. 6C), and took the *t* sum of the largest cluster in each permutation. We considered clusters in the original data as significant if their summed *t* values were higher than 99.5% or lower than 0.5% of the shuffled distribution (that is, *p* < 0.01, two-tailed) or higher than 97.5% or lower than 2.5% (*p* < 0.05), as specified in the main text. Significant clusters of WTPL in time and frequency (Fig. 7D, 7F) were defined with *t*-sums computed relative to a baseline before the visual stimulus or TMS pulses (–1 to –0.5 s), over 64 repetitions (2^6^, maximal permutation possible with six TMS subjects).

### Reporting summary

Further information on research design is available in the Nature Portfolio Reporting Summary linked to this article.

## Supplementary information

Supplementary Information

Reporting Summary

Transparent Peer Review file

## Source data

Source Data

## Data Availability

Dataset I used in this study is available in the Figshare database under accession code 10.6084/m9.figshare.29519285.v1 (https://figshare.com/articles/dataset/EEG_during_the_two-flash_task/29519285)^91^. Dataset II used in this study is available in the Figshare database under accession code 10.6084/m9.figshare.29519639.v1 (https://figshare.com/articles/dataset/EEG_at_wakeful_rest/29519639/1)^92^. Dataset III used in this study is available in the Figshare database under accession code 10.6084/m9.figshare.29519756.v1 (https://figshare.com/articles/dataset/EEG_during_tactile_timing_task/29519756/1)^93^. Dataset IV used in this study is available in the Harvard Dataverse database under accession code 10.7910/DVN/YD7PPU (https://dataverse.harvard.edu/dataset.xhtml?persistentId=doi:10.7910/DVN/YD7PPU)^94^. Dataset V used in this study is available under restricted access, as it is controlled by the original data providers; access can be obtained by contacting the corresponding authors of ref. ^68^, Mikael Johansson (mikael.johansson@psy.lu.se) and Robin Hellerstedt (robin.hellerstedt@ctb.upm.es). Dataset VI used in this study is available under restricted access, as it is controlled by the original data providers; access can be obtained by contacting the lead author of ref. ^69^, Pierre Gagnepain (pierre.gagnepain@inserm.fr). Dataset VII generated in this study is available in the Figshare database under accession code 10.6084/m9.figshare.27934962.v2 (https://figshare.com/articles/dataset/EEG_at_rest_and_with_TMS/27934962/2)^95^. Dataset VIII used in this study is available under restricted access; access can be obtained via the Cambridge Centre for Ageing and Neuroscience (Cam-CAN) data portal (https://camcan-archive.mrc-cbu.cam.ac.uk/dataaccess/) upon application and approval by the Cam-CAN data access committee. Dataset IX used in this study is available in the Open Science Framework database under accession code 10.17605/OSF.IO/4HXPW (https://osf.io/4hxpw/)^96^. Dataset X used in this study can be found in an online repository. The URL of the repository is: http://epi.fizica.unibuc.ro/scalesoldnew/. Dataset XI used in this study is available in the University of Oxford database under accession code 10.5287/BODLEIAN:MZJ7YWXVO (https://data.mrc.ox.ac.uk/stn-lfp-on-off-and-dbs)^97^. Dataset XII used in this study is available in the Collaborative Research in Computational. Neuroscience (CRCNS) database under accession code 10.6080/K09G5JRZ (https://portal.nersc.gov/project/crcns/download/hc-3)^98^. A small dataset generated in the study to demonstrate the LAVI algorithm is available at https://github.com/laaanchic/LAVI/blob/main/data.mat. Source data are provided with this paper.

## Code availability

A Matlab implementation for LAVI and ABBA (including pink surrogate and look-up table generation) is available at https://github.com/laaanchic/LAVI^79^.
