# Supplementary material for: Universal rhythmic architecture uncovers two modes of neural dynamics
Source: Nat Commun. 2026 May 30;17:7024. doi: 10.1038/s41467-026-73553-8 (PMC13392403; doi:10.1038/s41467-026-73553-8)
Supplement: Supplementary file 2 — Reporting Summary [file 41467_2026_73553_MOESM2_ESM.pdf]

nature portfolio

Corresponding author(s): Golan Karvat

Last updated by author(s): Apr 15, 2026

Reporting Summary

Nature Portfolio wishes to improve the reproducibility of the work that we publish. This form provides structure for consistency and transparency in reporting. For further information on Nature Portfolio policies, see our Editorial Policies and the Editorial Policy Checklist.

Statistics

For all statistical analyses, confirm that the following items are present in the figure legend, table legend, main text, or Methods section.

n/a	Confirmed	
☐	☒	The exact sample size (*n*) for each experimental group/condition, given as a discrete number and unit of measurement
☐	☒	A statement on whether measurements were taken from distinct samples or whether the same sample was measured repeatedly
☐	☒	The statistical test(s) used AND whether they are one- or two-sided<br>*Only common tests should be described solely by name; describe more complex techniques in the Methods section.*
☐	☒	A description of all covariates tested
☐	☒	A description of any assumptions or corrections, such as tests of normality and adjustment for multiple comparisons
☐	☒	A full description of the statistical parameters including central tendency (e.g. means) or other basic estimates (e.g. regression coefficient) AND variation (e.g. standard deviation) or associated estimates of uncertainty (e.g. confidence intervals)
☐	☒	For null hypothesis testing, the test statistic (e.g. *F*, *t*, *r*) with confidence intervals, effect sizes, degrees of freedom and *P* value noted<br>*Give P values as exact values whenever suitable.*
☐	☒	For Bayesian analysis, information on the choice of priors and Markov chain Monte Carlo settings
☒	☐	For hierarchical and complex designs, identification of the appropriate level for tests and full reporting of outcomes
☐	☒	Estimates of effect sizes (e.g. Cohen's *d*, Pearson's *r*), indicating how they were calculated

Our web collection on statistics for biologists contains articles on many of the points above.

Software and code

Policy information about availability of computer code

Data collection	TMS-EEG data in dataset VII were collected using BrainVision Recorder and preprocessed in Matlab 2020b (MathWorks, Natick, MA), with the Fieldtrip toolbox (release 20230822) and following previously published guidelines (Rogasch et al, NeuroImage, 2017; Zmeykina et al, Sci Rep, 2020); Stimulus and TMS pulses delivery was controlled using Psychtoolbox-3 in Matlab (Brainard, Spatial Vision, 1997).
Data analysis	The code and demo data for the algorithms central to the research are available on GitHub: https://github.com/laaanchic/LAVI and https://github.com/laaanchic/WTPL.<br>The MaxFilter 2.2.12 software (Elekta Neuromag Oy, Helsinki, Finland) was used to apply temporal signal space separation of MEG data, and later imported into Matlab using SPM12 (http://www.fil.ion.ucl.ac.uk/spm).<br>Pink noise simulation procedures are based on code contributed by Venema, V.: https://ch.mathworks.com/matlabcentral/fileexchange/4783-surrogate-time-series-and-fields (2023).<br>Bayes Factor (BF) t-tests or ANOVA were calculated using a BF toolbox for Matlab: Krekelberg, B. BayesFactor: Release 2022 (v2.3.0). Zenodo https://doi.org/10.5281/zenodo.7006300 (2022).

For manuscripts utilizing custom algorithms or software that are central to the research but not yet described in published literature, software must be made available to editors and reviewers. We strongly encourage code deposition in a community repository (e.g. GitHub). See the Nature Portfolio guidelines for submitting code & software for further information.

Data

Policy information about availability of data

All manuscripts must include a data availability statement. This statement should provide the following information, where applicable:

- Accession codes, unique identifiers, or web links for publicly available datasets
- A description of any restrictions on data availability
- For clinical datasets or third party data, please ensure that the statement adheres to our policy

Dataset I used in this study is available in the Figshare database under accession code 10.6084/m9.figshare.29519285.v1 (https://figshare.com/articles/dataset/EEG_during_the_two-flash_task/29519285) 91.
Dataset II used in this study is available in the Figshare database under accession code 10.6084/m9.figshare.29519639.v1 (https://figshare.com/articles/dataset/EEG_at_wakeful_rest/29519639/1) 92.
Dataset III used in this study is available in the Figshare database under accession code 10.6084/m9.figshare.29519756.v1 (https://figshare.com/articles/dataset/EEG_during_tactile_timing_task/29519756/1) 93.
Dataset IV used in this study is available in the Harvard Dataverse database under accession code 10.7910/DVN/YD7PPU (https://dataverse.harvard.edu/dataset.xhtml?persistentId=doi:10.7910/DVN/YD7PPU) 94.
Dataset V used in this study is available under restricted access, as it is controlled by the original data providers; access can be obtained by contacting the corresponding authors of ref. 68, Mikael Johansson (mikael.johansson@psy.lu.se) and Robin Hellerstedt (robin.hellerstedt@ctb.upm.es).
Dataset VI used in this study is available under restricted access, as it is controlled by the original data providers; access can be obtained by contacting the lead author of ref. 69, Pierre Gagnepain (pierre.gagnepain@inserm.fr).
Dataset VII generated in this study is available in the Figshare database under accession code 10.6084/m9.figshare.27934962.v2 (https://figshare.com/articles/dataset/EEG_at_rest_and_with_TMS/27934962/2) 95.
Dataset VIII used in this study is available under restricted access; access can be obtained via the Cambridge Centre for Ageing and Neuroscience (Cam-CAN) data portal (https://camcan-archive.mrc-cbu.cam.ac.uk/dataaccess/) upon application and approval by the Cam-CAN data access committee.
Dataset IX used in this study is available in the Open Science Framework database under accession code 10.17605/OSF.IO/4HXPW (https://osf.io/4hxpw/) 96.
Dataset X used in this study can be found in an online repository. The URL of the repository is: http://epi.fizica.unibuc.ro/scalesoldnew/.
Dataset XI used in this study is available in the University of Oxford database under accession code 10.5287/BODLEIAN:MZJ7YWXVO (https://data.mrc.ox.ac.uk/stn-lfp-on-off-and-dbs) 97.
Dataset XII used in this study is available in the Collaborative Research in Computational
Neuroscience (CRCNS) database under accession code 10.6080/K09G5JRZ (https://portal.nersc.gov/project/crcns/download/hc-3) 98.
A small dataset generated in the study to demonstrate the LAVI algorithm is available at https://github.com/laaanchic/LAVI/blob/main/data.mat.
Source Data are provided with this paper.

Research involving human participants, their data, or biological material

Policy information about studies with human participants or human data. See also policy information about sex, gender (identity/presentation), and sexual orientation and race, ethnicity and racism.

Reporting on sex and gender	We examined whether the rhythmicity architecture generalizes across sexes by analyzing a large cohort of participants (n = 625; 308 women [49.3%], 317 men [50.7%]). The results showed strong evidence for no sex-based differences (BF_{01} = 43.25). Consequently, data from female and male participants were pooled in subsequent analyses. To further establish rhythmicity architecture as a universal phenomenon, we analyzed a large body of existing data from multiple laboratories, comprising 859 participants of both sexes (52% female). Detailed sex composition for each dataset is provided below.<br>Datasets I-II: 30 women (71%) and 12 men (29%). Karvat, G., Ofir, N. & Landau, A. N. Sensory Drive Modifies Brain Dynamics and the Temporal Integration Window. Journal of Cognitive Neuroscience 36, 614–631 (2024).<br>Dataset III: 31 women (69%) and 14 men (31%). Ofir, N. & Landau, A. N. Neural signatures of evidence accumulation in temporal decisions. Current Biology 32, 4093-4100.e6 (2022).<br>Dataset IV: 17 women (59%) and 12 men (41%). Noguchi, Y. Individual differences in beta frequency correlate with the audio–visual fusion illusion. Psychophysiology 59, e14041 (2022).<br>Dataset V: 20 women (63%) and 12 men (38%). Hellerstedt, R., Johansson, M. & Anderson, M. C. Tracking the intrusion of unwanted memories into awareness with event-related potentials. Neuropsychologia 89, 510–523 (2016).<br>Dataset VI: 13 women (48%) and 14 men (52%). Legrand, N. et al. Attentional capture mediates the emergence and suppression of intrusive memories. iScience 25, 105516 (2022).<br>Dataset VII: four women (67%) and two men (33%) determined based on self-reporting particiapted in the study.<br>Dataset VIII: Taylor, J. R. et al. The Cambridge Centre for Ageing and Neuroscience (Cam-CAN) data repository: Structural and functional MRI, MEG, and cognitive data from a cross-sectional adult lifespan sample. NeuroImage 144, 262–269 (2017).<br>Dataset IX: 4 women (40%) and 6 men (60%). Vishne, G., Gerber, E. M., Knight, R. T. & Deouell, L. Y. Distinct ventral stream and prefrontal cortex representational dynamics during sustained conscious visual perception. Cell Reports 42, (2023).<br>Dataset X: 12 patients, gender/ sex undiscloased. Barborica, A. et al. Studying memory processes at different levels with simultaneous depth and surface EEG recordings. Front. Hum. Neurosci. 17, (2023).<br>Dataset XI: 2 women (14%) and 12 men (86%). Wiest, C. et al. Local field potential activity dynamics in response to deep brain stimulation of the subthalamic nucleus in Parkinson's disease. Neurobiology of Disease 143, 105019 (2020).<br>Dataset XII: 0 female (0%) and 11 male rats (100%). Mizuseki, K., Sirota, A., Pastalkova, E. & Buzsáki, G. Theta oscillations provide temporal windows for local circuit computation in the entorhinal-hippocampal loop. Neuron 64, 267–280 (2009).
Reporting on race, ethnicity, or other socially relevant groupings	Since we studied the rhythmic architecture as a universal phenomenon, race and ethnicity were not used in our manuscript to categorize participants.<br>Reports of race, ethnicity, or other socially relevant groupings of the different previously-collected datasets analyzed here are provided, when available, in their original publications. Details about race and ethnicity are not recorded for the volunteer

panel used in dataset VII.

Population characteristics

Participant characteristics (including age, gender, and clinical status) varied across datasets and are reported in detail in the original publications and below (see Research Sample). In brief, healthy adult participants were predominantly young adults recruited from university communities, whereas patient datasets comprised individuals undergoing intracranial monitoring for clinical purposes. Where relevant, these variables were considered in the original studies; the present analyses focus on aggregate neural signatures across datasets. Covariate-relevant population characteristics of the human research participants (age and sex) were addressed directly based on data from dataset VIII (Cam-CAN), and reported in figure 5 and 6.

Recruitment

Reports of recruitment procedures of the different previously-collected datasets analyzed here are provided, when available, in their original publications (and see above, availability of data). For the TMS-EEG study (dataset VII), participants were recruited from a panel of volunteers interested to participate in neuro-psychological studies (https://www.mrc-cbu.cam.ac.uk/take-part/).
Healthy adult datasets are subject to standard self-selection and convenience sampling biases associated with university-based recruitment, including over-representation of younger, highly educated populations.
Patient datasets are subject to selection biases inherent to clinical populations undergoing intracranial monitoring, which may limit generalisability.
However, as the present study focuses on fundamental neural dynamics rather than population-level behavioural differences, these biases are unlikely to substantially impact the main conclusions. The consistency of findings across multiple independently collected datasets further mitigates concerns related to sampling-specific biases.

Ethics oversight

The details of the local organizations approving the protocols of the different datasets used in the manuscript are provided in the original publications.
Approval for protocols for the new data collected here was given by Cambridge Psychology Research Ethics Committee. All participants provided written informed consent prior to data acquisition for the study.

Note that full information on the approval of the study protocol must also be provided in the manuscript.

Field-specific reporting

Please select the one below that is the best fit for your research. If you are not sure, read the appropriate sections before making your selection.

☐ Life sciences ☒ Behavioural & social sciences ☐ Ecological, evolutionary & environmental sciences

For a reference copy of the document with all sections, see nature.com/documents/nr-reporting-summary-flat.pdf

Behavioural & social sciences study design

All studies must disclose on these points even when the disclosure is negative.

Study description

Data are quantitative, including recording of electrophysiological activity (EEG/ MEG/ intracranial electrodes) at rest or in response to TMS stimulation.

Research sample

Since we studied the rhythmic architecture as a universal phenomenon, we analyzed a big and various research sample, collected in different laboratories and with varying ecquisition techniques. Details on the samples used in these existing datasets are provided below based on the original publications:
Datasets I-III: The original datasets comprised 45 participants in Datasets I–II and 31 participants in Dataset III. Data from eight participants in Datasets I–II were excluded due to technical issues (poor or missing EEG signal), and data from two participants in Dataset III were excluded due to technical issues (poor signal quality or missing triggers). The final samples comprised 37 participants in Datasets I–II (27 women, 10 men; age = 22.9 ± 2.6 years, mean ± SD) and 29 participants in Dataset III (16 women, 13 men; age = 24 ± 3.1 years, mean ± SD). Sex was determined based on participant self-report in the original studies. Participants were recruited from a university population. All procedures were approved by the institutional review board of the Hebrew University of Jerusalem, and participants provided informed consent. Participants were compensated either monetarily (10 € per hour) or with course credit.
Dataset IV: The sample comprised 29 participants (17 women, 12 men; age range = 18–42 years). Data from two participants were excluded due to excessive EEG noise and replaced to maintain sample size in the original study. Sex was determined based on participant self-report in the original study. All procedures were approved by the ethics committee of Kobe University, Japan, and participants provided informed consent.
Dataset V: The original dataset comprised 36 participants. In the original study, data from four participants were excluded, resulting in a sample of 32 participants (20 women, 12 men; mean age = 25 years, range = 20–34 years). In the present study, data from nine participants were excluded due to excessive EEG artefacts or poor data quality, yielding a final sample of 27 participants. Sex was determined based on participant self-report in the original study. All procedures were approved by the regional ethics committee at Lund University, and participants provided written informed consent. Participants were compensated with two cinema tickets.
Dataset VI: The sample comprised 24 participants (age range = 18–35 years). Sex information was not reported in the original study and could not be reconstructed from the available data. All procedures were conducted in accordance with institutional guidelines, and participants provided informed consent.
Dataset VII: Twelve participants were recruited via internet-based advertisements at the MRC Cognition and Brain Sciences Unit SONA participant pool. Six participants withdrew due to discomfort during frontal rTMS. The final sample comprised six participants (four women, two men; age = 27.1 ± 7.1 years, mean ± SD). Sex was determined based on participant self-report. All procedures were approved by the Cambridge Psychology Research Ethics Committee, and participants provided written informed consent prior to participation. Participants were compensated £12 per hour.
Dataset VIII: MEG data were drawn from Stage 2 of the Cambridge Centre for Ageing and Neuroscience (Cam-CAN; www.cam-can.org) study, a population-based adult lifespan cohort (18–88 years) . Ethical approval was obtained from the East of England–

Cambridge Central Research Ethics Committee, and participants provided written informed consent. Participants were recruited from the general population via primary care registers. The present study included 625 participants (317 women, 308 men) with resting-state MEG data suitable for analysis following motion correction. Sex was recorded in the original dataset based on participant self-report and was approximately balanced across the lifespan sample. Sex differences were explicitly examined in the present study (see Results, Fig. 5E–F), revealing strong evidence for no sex-based differences. Further details on inclusion and exclusion criteria are provided in the original report (see Table 1 in Shafto et al., BMC Neurol, 2014).

Dataset IX: Intracranial electrophysiological (ECoG) data were obtained from 10 patients (4 women, 6 men; age = 41.0 ± 12.2 years, mean ± SD) with drug-resistant epilepsy undergoing clinical monitoring with subdural electrodes. Electrode implantation sites were determined solely based on clinical considerations. Participants were recruited from clinical centres (Stanford School of Medicine, California Pacific Medical Center, and UCSF Medical Center). All participants provided written informed consent, and all procedures were approved by the UC Berkeley Committee on Human Research and the corresponding institutional review boards at the clinical recording sites.

Dataset X: Simultaneous scalp EEG and stereoelectroencephalography (SEEG) data were obtained from 12 patients (age = 27.5 ± 6.3 years, mean ± SD) with drug-resistant epilepsy undergoing pre-surgical clinical evaluation. Sex information was not reported in the original study and could not be reconstructed from the available data. Electrode implantation and placement were determined solely based on clinical considerations. Participants were recruited from the Emergency University Hospital Bucharest. All participants (or their legal guardian/next of kin) provided written informed consent, and all procedures were approved by the University of Bucharest Ethics Committee for Research.

Dataset XI: Local field potential (LFP) recordings were obtained from 17 patients (4 women, 13 men; age = 56.4 ± 6.7 years, mean ± SD) with Parkinson's disease undergoing deep brain stimulation of the subthalamic nucleus. Recordings were performed postoperatively while electrode leads were externalised, and electrode placement was determined solely based on clinical considerations. Participants were recruited at St George's University Hospital and King's College Hospital (London, UK), and the University Medical Center Mainz (Germany). All participants provided written informed consent, and all procedures were approved by the relevant local ethics committees.

Dataset XII: LFP and single-unit recordings were obtained from five male Long-Evans rats (250–400 g). Animals were implanted with multisite silicon probes targeting the medial entorhinal cortex and hippocampus and recorded during freely behaving spatial tasks. All experimental procedures were approved by the Institutional Animal Care and Use Committee of Rutgers University.

Sampling strategy

The datasets analysed in this study were collected as part of previously published experiments. For healthy adult participants, recruitment in the original studies primarily relied on convenience sampling from university communities and surrounding populations, occasionally with efforts to balance gender. As such, these samples are characteristic of typical WEIRD populations.

For intracranial (epilepsy and PD) patient datasets, participants were recruited based on clinical indications, with inclusion and exclusion criteria defined in the original studies.

No new sample size calculations were performed for the present study, as all available data from the original datasets were included. The current analyses do not aim to test specific effects from the original studies but instead leverage aggregated datasets to characterise generalisable neural signatures. The large combined sample size across datasets provides substantial statistical power and robustness for the present purposes.

For dataset VII generated in this study is, participants were recruited from a panel of volunteers interested to participate in neuro-psychological studies (https://www.mrc-cbu.cam.ac.uk/take-part/). Since the study was designed with an exploratory nature, it was not possible to estimate effect size and variation a-prioi, which are necessary for power analysis. As an alternative, we used the 'resource equation' approach, stating that the acceptable range of degrees of freedom for the error term is between 10 to 20 (Festing & Altman, doi: 10.1093/ilar.43.4.244; Arifin & Zahiruddin, doi: 10.21315/mjms2017.24.5.11; http://wnarifin.github.io). For repeated measures within subjects, the minimal number of participants can be determined by the formula: n = ceil (10/(b–1) + 1). For b = 3 bands (alpha, beta 1, and beta2), the minimal n is 6 (as is the final sample size in this study).

Data collection

Reports of data collection of the different previously-collected datasets analyzed here are provided in their original publications (and see above, Availability Of Data and Research Sample).

In dataset VII, Biphasic single and repetitive TMS pulses were delivered using a DuoMAG XT-100 TMS stimulator and a figure-of-eight coil DuoMAG 70BF (Brainbox Ltd, Cardiff, UK). During the stimulation, participants sat in a comfortable recliner chair with a neck rest. To ensure the precise targeting of specific brain regions, the coil was controlled via Brainsight 2 neuronavigation system (Brainbox) in combination with an Axilum TMS-Cobot (Axilum Robotics, Schiltigheim, France). The Cobot is a robotic system that actively monitors and adjusts the positioning of the coil, and compensates for head movements throughout the experiment. To detect head movements, participants wore a headband reference tracker that was monitored by a Polaris Vega ST camera (NDI, Waterloo, Canada). The TMS coil was oriented with the handle pointing posteriorly with respect to the participant's head, at an angle of 45 degrees relative to midline. The MNI coordinates for dorso-lateral prefrontal cortex (dlPFC, x = 33, y = 39, z = 26) stimulation were derived from the peak voxel showing the strongest effect in BOLD signal in a previous meta-analysis study (Apšvalka et al, Nat Commun, 2022).

Our protocol established a TMS stimulation intensity at 90% of the resting motor threshold (RMT) of each participant. To determine the RMT, we positioned the coil over the hand area of the right primary motor cortex and asked the participant to keep their left hand relaxed and at rest. Then, we determined the minimum intensity at which a single TMS pulse produced a visible twitch in the abductor pollicis brevis muscle of their left hand, in five of ten successive pulses.

EEG recordings were obtained with the actiCHamp Plus 64 system (Brain Products GmbH, Gilching, Germany), which is TMS-compatible. The system includes a DC-coupled amplifier avoiding AC recoding and high-pass filter during the recording period. EEG signals were acquired from 64 active electrodes arranged on an actiCAP slim electrode cap. The ground electrode was placed at FPz, and the reference at Cz. Electrode impedance was maintained below 20 kOhm. We used a sampling rate of 1000 Hz for the EEG resting-state recording and 5 kHz for the TMS-EEG recordings.

After EEG setup, we used LAVI and ABBA to determine the individual alpha, beta1, and beta2 peak frequencies that would define the repetitive TMS stimulation frequencies. For this, we performed a resting-state EEG recording of 12 minutes (2-minutes with open eyes, 8-minutes closed eyes, and 2-minutes open eyes). During the fixation periods, participants were instructed to keep their eyes still and look at a white fixation cross presented on a black background. LAVI was calculated over the closed-eyes period.

The TMS-EEG session comprised a total of 480 trials distributed into 8 blocks. There were 7 conditions: 1 single pulse (sp-TMS), 3 rhythmic conditions consisting of a 6-pulses train at alpha, beta1 or beta2 individual frequencies, and 3 arrhythmic conditions, also consisting of 6-pulses train. For each arrhythmic condition we precomputed patterns of 6-pulses that excluded frequencies within a +/-2-Hz band centred on the frequency of the corresponding rhythmic condition, their harmonics, subharmonics and over 50 Hz. The length of both rhythmic stimuli was equal to 5 cycles of the peak frequency. Each block included 12 trials of the single pulse condition

	and 8 trials of each of the rhythmic and arrhythmic conditions, presented sequentially in a pseudorandomized order. During each block, participants were instructed to keep their eyes still and look at a white fixation cross presented on a black background. The inter-train interval (ITI) between two consecutive trials was adjusted to the preceding frequency according to safety guidelines (Rossi et al., Clinical Neurophysiology, 2009). For frequencies up to 10 Hz, we used a 3-s ITI; for frequencies over 10 Hz and up to 15 Hz, we used a 5-s ITI; for frequencies over 15 Hz and up to 20 Hz, we used an 8-s ITI; for frequencies over 20 Hz and up to 25 Hz, we used a 10-s ITI. Stimulus and TMS pulses delivery was controlled using Psychtoolbox-3 in Matlab.<br>During data collection, only the participant and two experimenters (G.K. and M.C.G.) were present in the room. The experimental design was within-subject with randomized condition order. Experimenters were not formally blinded to condition, however, the automated delivery and randomized design minimized potential experimenter bias.
Timing	Reports of timing of the different previously-collected datasets analyzed here are provided, when available, in their original publications (and see above, availability of data).<br>TMS-EEG data (dataset VII) were collected in one cohort from Aug. 29th, 2023 to Oct. 10th, 2023.
Data exclusions	Reports of data exclusions for the previously collected datasets analysed here are provided, where available, in the original publications (see also "Research sample").<br>For the TMS–EEG dataset generated in this study, data from all participants who completed the experiment (n = 6) were included in the analyses; no datasets were excluded.
Non-participation	Reports of non-participation for the previously collected datasets analysed here are provided, where available, in the original publications (see also "Research sample").<br>For the TMS–EEG dataset generated in this study, 6 out of 12 participants discontinued participation due to discomfort during frontal rTMS.
Randomization	Participants were not allocated to experimental groups. The study employed a within-subject design with randomized condition order.

Reporting for specific materials, systems and methods

We require information from authors about some types of materials, experimental systems and methods used in many studies. Here, indicate whether each material, system or method listed is relevant to your study. If you are not sure if a list item applies to your research, read the appropriate section before selecting a response.

Materials & experimental systems

n/a	Involved in the study
☒	☐ Antibodies
☒	☐ Eukaryotic cell lines
☒	☐ Palaeontology and archaeology
☒	☐ Animals and other organisms
☒	☐ Clinical data
☒	☐ Dual use research of concern
☒	☐ Plants

Methods

n/a	Involved in the study
☒	☐ ChIP-seq
☒	☐ Flow cytometry
☐	☒ MRI-based neuroimaging

Plants

Seed stocks	*Report on the source of all seed stocks or other plant material used. If applicable, state the seed stock centre and catalogue number. If plant specimens were collected from the field, describe the collection location, date and sampling procedures.*
Novel plant genotypes	*Describe the methods by which all novel plant genotypes were produced. This includes those generated by transgenic approaches, gene editing, chemical/radiation-based mutagenesis and hybridization. For transgenic lines, describe the transformation method, the number of independent lines analyzed and the generation upon which experiments were performed. For gene-edited lines, describe the editor used, the endogenous sequence targeted for editing, the targeting guide RNA sequence (if applicable) and how the editor was applied.*
Authentication	*Describe any authentication procedures for each seed stock used or novel genotype generated. Describe any experiments used to assess the effect of a mutation and, where applicable, how potential secondary effects (e.g. second site T-DNA insertions, mosiacism, off-target gene editing) were examined.*

Magnetic resonance imaging

Experimental design

Design type	Resting-state, anatomical
Design specifications	One block, ~ 5 minutes
Behavioral performance measures	No behavioural performance

Acquisition

Imaging type(s)	Structural
Field strength	3T
Sequence & imaging parameters	Pulse sequence type: Gradient echo or spin echo<br>Imaging type: EPI, spiral<br>Field of view: 100<br>Matrix size: [0,256,246,0]<br>Slice thickness: 1<br>Orientation:<br>TE/TR/flip angle: 3.02/ 2250/ 9
Area of acquisition	Whole brain

Diffusion MRI ☐ Used ☒ Not used

Preprocessing

Preprocessing software	SPM12 (dicom to nii, coregistration to MNI template ICBM152)
Normalization	Linear coregistration
Normalization template	MNI template ICBM152
Noise and artifact removal	N/A (MRI was used only for structural anatomy, hence no further preprocessing was performed)
Volume censoring	N/A (MRI was used only for structural anatomy, hence no further preprocessing was performed)

Statistical modeling & inference

Model type and settings	MRI was used only for structural anatomy, hence no further statistical tests performed.
Effect(s) tested	MRI was used only for structural anatomy, hence no further statistical tests performed.

Specify type of analysis: ☒ Whole brain ☐ ROI-based ☐ Both

Statistic type for inference<br>(See Eklund et al. 2016)	MRI was used only for structural anatomy, hence no further statistical tests performed.
Correction	MRI was used only for structural anatomy, hence no further statistical tests performed.

Models & analysis

n/a	Involved in the study
☒	☐ Functional and/or effective connectivity
☒	☐ Graph analysis
☒	☐ Multivariate modeling or predictive analysis
