# Supplementary material for: Universal rhythmic architecture uncovers two modes of neural dynamics
Source: Nat Commun. 2026 May 30;17:7024. doi: 10.1038/s41467-026-73553-8 (PMC13392403; doi:10.1038/s41467-026-73553-8)
Supplement: Supplementary file 3 — Transparent Peer Review file [file 41467_2026_73553_MOESM3_ESM.pdf]

nature portfolio

Peer Review File

Universal rhythmic architecture uncovers two modes of neural dynamics

Corresponding Author: Dr Golan Karvat

This file contains all reviewer reports in order by version, followed by all author rebuttals in order by version.

Version 0:

Reviewer comments:

Reviewer #1

(Remarks to the Author)
In this paper, Karvat and colleagues studied brain rhythmic activity, incorporating several existing datasets in their analysis from humans and rodents, obtained with different recording modalities, and one novel dataset they recorded in their lab combining EEG/TMS. They perform a comparative study across species, modalities, behavioral tasks/states and age, with the aim to determine if there is a rhythmic architecture with distinct modes that is comparable across the different datasets. We consider this topic of high interest, and the authors addressed this question from a novel and interesting point of view. During the last decade more and more studies have moved away from considering brain rhythms as sustained oscillations, and embraced the notion of transient rhythmic bursts, which can best be studied at the single trial level. We still need a good description and understanding of brain rhythms in this novel perspective of transient bursts, and this study can provide a valuable contribution to this.
We appreciate the effort to include a large variety of datasets, with the aim of determining the universal aspect of their findings, across modalities, species etc. This is a strength of your paper. However, we miss that you go in depth in the individual datasets, in particular in individual subjects. While developing general principles based on common findings across large datasets is very valuable, and useful to build models aimed to gain mechanistic understanding, this also washes out any details, somewhat hindering this mechanistic understanding.
For instance, one the one hand, to which extent can individual subjects have all the transient and sustained bands you describe (which is not clear in Fig. 1 E/F). On the other hand to which degree can one specific band be both transient and sustained within the same subject, depending e.g. on task epoch or behavioral state. In the analysis all data is collapsed when estimating rhythmicity for each frequency, such that within one subject each frequency only can get one label.
It is difficult to understand Fig. 1E/F, but one way to interpret it is that only a fraction of subjects actually present anyone of the sustained/transient bands they describe, thus the general principles seem not systematically observed in individual datasets and subjects? Otherwise, how to interpret the fractions for each frequency? This needs clarification, or maybe a different way to visualize. Furthermore, if one would look at a specific frequency, such as e.g. 6Hz, of the fraction of subjects that have significantly sustained or transient in this epoch, it seems to split 50/50 between the two categories?
Thus, are you claiming that the exact bands that you find universally have a ‘transient’ or ‘rhythmic’ nature, so that for instance beta1 is in general a transient band and beta2 is a sustained band? In this case, it is a bit disturbing that you set a very broad range of the alpha band that you use to label the other bands, and that the method will assign the names of the frequency bands always relative to the “standard” alpha peak. In Fig1-L it is stated that when the dataset was published the band was considered Theta, yet here the band would be assigned to Alpha based on the ABBA algorithm. How can we understand the claims directed to specific frequency bands? In this case the effect of theta would be hidden inside the alpha behavior… Such a strong claim, of e.g. beta1 being transient and beta2 sustained, seems indeed to be what you aim for, given your discussion on page 10.
Or are you more loosely claiming that there will be transient and sustained/rhythmic bands, and that the sustained ones will dominate the power spectrum, but that which bands are either or can change across subjects and or datasets?
Furthermore, you link transient and sustained bands to rate code and phase code, respectively. However, you never seem to actually test phase coding. Since you find that transient bands correspond to <4 cycles and sustained/rhythmic as >6 cycles (P8), both transient and sustained bands have ‘bursts’, that mainly differ in duration. However, even a few cycles in a transient burst to some extent suggest phase coding, and your link with high-frequency was an amplitude co-variance analysis? Do you need to explicitly make the link to phase/rate coding? If so, then this should maybe be tested more directly?
It is also somewhat unclear why the sustained bands dominate the power spectrum, since the shorter-duration transient

bursts to some extent 'compensate' by being more frequent (Fig. 2A)? Do the sustained bands also reflect bursts with higher instantaneous amplitude, or can transient bursts (<4 cycles) have similar amplitude as sustained bursts (>6 cycles)? Then, to go into more detail to understand the underlying mechanisms, to which degree would frequency drift within long bursts tend to limit the LAVI measure, making the signal less rhythmic, and then defined as more transient? For instance, Atallah and Scanziani (2009) showed that the frequency of gamma bursts can change from cycle to cycle in the rodent hippocampus (interestingly linked to amplitude in the preceding cycle). Frequency drift within individual burst can also be observed in data from the macaque prefrontal cortex (e.g. Lundqvist et al. 2016, Fig. 4a), even if not necessarily highlighted by the authors.

Furthermore, Kilavik et al. 2012 showed that in the macaque motor cortex both low and high beta (i.e. beta1 and beta 2) are present in delays and in epochs immediately following an informative visual cue (more transient and with slightly increased frequency and lower power after visual cues). Thus both beta1 and beta2 can be linked with epochs of a task typically associated with sustained and transient (evoked) activity in the same subject. Although they did not study bursts, this is linked to the questions above, whether one band can be transient in one context and rhythmic/sustained in another?

It is clear that the choice of lag does not influence the relative results - and that the specific lag used is optimal for not flooring or ceiling the results. The method is working well for finding significantly bursty and significantly oscillatory events linked to specific frequencies. However, if two 'oscillatory' or 'bursty' frequencies are too close in peak frequency, the ABBA method will collapse this into one same band, e.g. Fig1-D inside the beta2 band; in this case the second peak is clearly stronger, but if both were comparable in amplitude, only the maximum would be chosen, disregarding the possibility of having two different frequency bands in a small frequency range of same sustained or transient nature?

I guess that it would be natural that you cite Rosanova et al. 2009, or other more recent studies for the TMS section? This brings us to the point of the largely lacking discussion section. This seems rather awkward given the huge amount of data covered in the study, the novel methods developed, and novel concepts coined. Is this a feature of the specific journal? Otherwise it would seem natural to discuss the potential limits of the methods, or other comparable approaches?

Minor
We never see raw data, beyond a glimpse of 1 subject in 1C.
Why only a selection of EEG datasets in E and F, instead of all 6 EEG datasets?
L96 Not necessarily a novel category of bands, as many recent papers suggest that bursts are very transient (i.e. below your 4 cycles), e.g. Feingold et al. 2015 (2-3 cycles in monkey basal ganglia), or Bonaiuto et al. 2021(<2 cycles in human MEG). Even what you refer to as sustained is in reality transient, as even if it outlasts 6 cycles, it is not truly sustained in time…

L119 LAVI is only explained in the figure legend (and not here in the results)(and then also in the methods…) I would find it natural to describe it a bit better here in the results, as you aim for 'online' methods…

L148 ABBA was not explained in enough detail to understand the results/figure legend… While readers can go to online methods to get details for an analysis or to replicate it, they should get enough information from the main manuscript to grasp intuitively how it works. In particular, this also renders Fig. 1E/F difficult to interpret.

L183-187 You here use oscillations to also talk about transient bursts. Maybe there should be a clear set of definitions somewhere, as to me an oscillation reflects both 'sinusoidal' and sustained…

Fig. 1 C/D if these are the same subject, should the 10Hz label in C rather be 12Hz, since in D 10Hz is neither rhythmic nor transient?

When calculating the WTPF across subjects, it would be nice to see how the peaks of frequencies are distributed in the subjects shown in Fig2-F. Are all of the subjects the ones from Fig1-G?

When calculating the HF response and covariance with LF amplitude, what happens if the channel response is not systematic across frequency ranges or analysis windows? If HF is significantly increasing in one of the windows and significantly decreasing in another, what is considered for that channel? Is it the biggest modulation for deciding whether it is positively or negatively responding? Is it rejected?

Results P3-L115/116. The sentence "we found that the relative rhythmicity between frequencies of individual subjects remains similar over lags" it's complicated to understand. Rephrase for something like "the choice of lag does not influence the relative rhythmicity values obtained across the different frequencies for each individual subject".

Fig 2. Center in 0 the colormap of the WTPL

Typos:
P11-L428: "rhythmicity may index may reflect/explain variability between individuals .."
P8-L261: Fig 2C before Fig 2B

Sincerely, Bjørg Kilavik and Laura Lopez-Galdo

(Remarks on code availability)

Reviewer #2

(Remarks to the Author)
I co-reviewed this manuscript with one of the reviewers who provided the listed reports. This is part of the Nature Communications initiative to facilitate training in peer review and to provide appropriate recognition for Early Career Researchers who co-review manuscripts.

(Remarks on code availability)

Reviewer #3

(Remarks to the Author)
General Comments

This is an ambitious, interesting, and well executed study with deep theoretical implications. It also introduces a novel phase-consistency measure (LAVI) that has significant advantages over lagged coherence. The text and some figures require a substantial re-organization for greater clarity; the current organization results in a chaotic exposition. I recommend that the ms be resubmitted after revisions.

The key conclusions stated in the Abstract are:

1. "we found a universal spectral architecture divisible into two categories: high-rhythmicity bands associated with sustained oscillations and novel low-rhythmicity bands dominated by brief oscillatory bursts"
2. "sustained bands signify maintenance of ongoing activity, whereas transient bands signify the brain's response to change"

The first conclusion is well supported by the data. The second conclusion makes a great deal of theoretical sense, but seems less well supported by the data. Perhaps if the manuscript were re-organized to make a clear separation between theoretical considerations and interpretation of data, the empirical support for conclusion #2 would become clearer. In its current form, conclusion #2 appears to be based entirely on data that are not shown.

There are also "four testable predictions" that are listed in the Introduction:

"(i) There should be defined frequency “sustained bands” that clearly express sustained oscillations.

(ii) Additionally, there should be a separate, novel category of bands specializing in transient activity. In these “transient bands”, spectral phenomena should manifest as short-lived bursts.

(iii) The architecture is a universal organizing principle of the brain's operating system; hence it should arise across datasets, recording techniques, brain areas, and species.

(iv) The architecture mediates cognition, hence its operation should be sensitive to variations in cognition arising with age and neuropathology, and to different cognitive states, such as input processing."

Predictions (i), (ii) and (iii) are confirmed by the presented data.

Frequent reference is made to "our universal rhythmic architecture hypothesis", but that hypothesis is never clearly articulated. It would help to describe the hypothesis in full detail in the Introduction.

The TMS experiments in general seem less convincing than the natural frequency band analyses.

Specific Comments

Lines 87-88: "Indeed, we recently showed that momentary neuronal oscillations (bursts) occur together with transient firing-rate changes". The word "indeed" implies that the result cited supports Roelfsema's contention that binding is accomplished by rate-coding, but it does not. It is to be expected that oscillatory activity and rate increases go together for simple oscillatory circuits like PING/ING (see e.g. Whittington et al. 2011, Developmental Neurobiology 71, 92–106) regardless of whether either one of them has anything to do with binding.

Lines 91-92: "sustained oscillations support phase-coding and maintenance of ongoing activity, whereas bursts reflect rate-coding and response to change" The conflation of the burst-versus-sustained distinction with the rate-coding-versus-phase-coding distinction goes well beyond the scope of the present paper, and is theoretically troublesome as well. Phase-coding inherently conveys information much more rapidly than rate-coding, and is therefore better suited to transmission by bursts, whereas rate-coding requires a sustained response whose duration is roughly proportional to the degree of precision required by the next layer of neural processing.

114-16: "we found that the relative rhythmicity between frequencies of individual subjects remains similar over lags." This

sentence is hard to understand. After having read the whole paper, I can retrospectively infer that the authors are saying that the authors get more or less the same sustained oscillation bands and burst oscillation bands from the same subject regardless of the lag used to perform the analysis. However, none of those concepts is explained until later in the paper. Also the use of the term "rhythmic" to mean "sustainedly oscillating" further muddies the presentation. Oscillatory bursts can be just as rhythmic as sustained oscillations, the rhythm just lasts for a shorter time.

116-118: "Hence, with carefully chosen parameters, the rhythmicity spectrum can be derived from a single lag (for further discussion, see Supplementary Text, parametrical effects on rhythmicity, and fig. S5)." Given that the main point of Figs. S5A and S5B is that one does not need to be particularly careful in choosing the delay value, it seems odd to say "with carefully chosen parameters". I think it might be a mistake to relegate Figs. S5A&B to the supplement rather than featuring them in the main text, because they are so central to the issues being explored. Also, some of the exploration of the properties of lagged coherence and LAVI could be profitably moved into the main text. It should at least be noted that the LAVI in the "sustained" bands is only two times higher than that in the "bursty" bands. This suggests that "bursty" bands contain bursts about half of the time, i.e. the time between bursts is similar to the durations of the bursts.

129: "rhythmicity bands" - no, these are "phase-consistency bands", and we need references to Figures 1 and S5 to support the statement. "Rhythmicity" is a more complicated concept that should be discussed in the text, but instead it is used interchangeably with "phase-consistency". As used in this manuscript, "rhythmicity" is introduced as meaning "sustained vs. bursty" in lines 27-28, which is an excellent first-order definition. Some authors would also insist that "rhythmicity" implies the existence of an underlying rhythm-generator circuit, and there is a simple form of evidence for such circuits that is available in certain types of experiments: if a rhythm can be induced and/or entrained (e.g. by periodic electrical or sensory stimulation), and the rhythm persists for at least a cycle or two after the end of the stimulation, then there must be an underlying rhythm-generator. (Failure to show this does not prove the non-existence of an underlying rhythm-generator, however.) Other expected characteristics of rhythmicity typically include relative constancy of frequency and amplitude. The problematic word here is "relative", because biological oscillators often due drift substantially in frequency and/or amplitude, and this is very likely the reason why a lag of 3 cycles (Fig. S5B) fails to detect phase-consistency bands. In other words, the typical duration of "relative constancy" of frequency in a sustainedly-oscillatory band seems to be about the same as the duration of a typical burst in a bursty band. Ultimately, the gold standard proof of rhythmicity is to elucidate the cellular (or sub-cellular) mechanism of the underlying rhythm-generator.

151-152: The text describes Fig. 1E as "the distribution of peak frequencies of each rhythmicity band across subjects", but there is a disconnect between the bands shown in Fig. 1E&F and those shown in Fig. 1G-J, particularly regarding the beta1 and beta2 bands. The peak of "beta2" spans roughly the 20-28 Hz range in Fig. 1E (roughly the central 50% of the distribution as judged by eye), but about 18-22 Hz in Fig. 1G-J and L ("95% confidence interval per group/ area"). Similarly, the central ~50% of the "beta1" distribution runs 15-20 Hz in Fig. 1E, but the 95% CLs run more like 12-16 Hz in Fig. 1G-J and L. These discrepancies require some explanation. Additional reasons why they are troublesome are described below.

165-166: "transient bands were as common as sustained bands" This paragraph begins with the sentence, "Having confirmed the existence of sustained bands, we next examined whether transient bands exist", but simply showing that some bands have higher phase-consistency than others does not prove that "transient bands" exist. (It should also be noted that it does not prove that "sustained bands" exist either, and the claim should not be made that their existence has been confirmed either.) Therefore, this is much too early to refer to "transient bands" as if their existence has already been proven. It remains to be shown that there actually are transient bursts of oscillation in these bands, and that the lower phase-consistency is due to the extreme brevity of these bursts. (Unfortunately, the actual demonstration of oscillatory bursts, which is well described in Online Methods section "Bursts detection", is buried in a section that superficially seems to be about phase-versus-rate coding and TMS, "Sub-second manifestations of rhythmicity".)

219-221: "Our hypothesis posits that the universal rhythmic architecture supports both rate- and phase-coding, by allocating “bursty” activity to transient-bands, and “genuine” oscillations to sustained-bands." This is another unfortunate sentence that simultaneously conflates the rate-versus-phase coding issue with the bursty-versus-sustained issue, reveals the excellent depth of theoretical thinking that lurks between the lines, and also implies that brief oscillations are inherently less "genuine" than sustained oscillations. It might be a good idea to defer the deep theoretical discussion to the Discussion section (and if necessary the Supplemental materials) and stick with data-descriptive terminology here. It would certainly be an improvement to avoid implying that rate-versus-phase coding is the same distinction as bursty-versus-sustained oscillation, regardless of where these two issues come up. However, this or one of the earlier passages cited above would be a good place to clarify what is meant by "genuine" versus "false" oscillations. Genuine oscillations come from oscillators, and false ones do not. Jones et al. (2007) Journal of Neuroscience, October 3, 2007 • 27(40):10751–10764 provides a wonderful example of how what appears to be a 1.5-cycle burst of beta oscillation can be explained by a cellular mechanism that superposes two bell-shaped bursts of activity that have different temporal widths but the same peak time to produce exactly the right waveform. If the proposed mechanism is correct, the waveform would have to be called a "false" oscillation because there is nothing oscillatory about the underlying mechanism. The waveform itself, unfortunately, provides no clues as to whether it is a false oscillation produced as proposed, or a true oscillation produced by a beta-frequency oscillator that somehow gets disabled almost immediately after it starts. It is unfortunate that no waveforms of transient versus sustained oscillations are shown in the figures. This would be a great enhancement to the paper.

237-240: The ANOVA results are very difficult to unpack from this block of text. They would be enormously much clearer if they were provided in tabular form and simply referenced in the text.

240-242: "Post-hoc Tukey Honest Significant Difference tests revealed higher burst-rates and shorter burst-durations in all

transient bands compared to their neighbouring sustained bands." Finally, we have the result that low phase-consistency bands truly are dominated by brief bursts of oscillation! This result also makes good on the claim that higher phase-consistency bands show fewer but longer periods of oscillation, i.e. "sustained" oscillations. It comes as a surprise, however, how little difference there is in duration between low phase-consistency band oscillations (about 2.2 cycles on average) and high phase-consistency (about 2.6 cycles on average).

It is a shame that the introductory paragraph of this section is so full of distractors. This problem could easily be solved by devoting a whole section to the proof of the fact that low phase-consistency bands are bursty bands, and this proof certainly deserves a whole section to itself. The histograms of Fig. 2A are much appreciated, and should be mentioned in the text in place of the ANOVA results. This would be a good place to show waveforms and describe the differences between sustained and transient bands. A plot similar to Figure 2C but for the physiological data would be very helpful as well. (It is true that Fig. 2C shows only the surrogate data, isn't it? This should be more clearly stated in the figure legend.) The analysis of the surrogate dataset with random phases is a good control analysis, and belongs in the same section, as does the analysis of the simulated data in lines 258-271.

272-273: "To causally demonstrate periodic input's role in rhythmicity, we stimulated six participants' prefrontal cortices with TMS (Fig. 2D)." This is a completely separate topic and belongs in a separate section, and Figs. 2D-F belong in a separate figure as well. To quote from the Nature Communications submission guidelines, "Depending on the word count, Articles may have up to 10 display items (figures and/or tables)." There is no need to cram separate topics into a single figure when you only have a total of 4 display items in the manuscript. The rest of this paragraph illustrates particularly well what I think is the chief defect of the whole manuscript, namely that empirical results are combined with thought-provoking theoretical issues and ill-founded claims of making successful predictions (in this case not because they were successful, but because they were trivial). It is as if an excellent, well-structured paper had been put into a blender, and we are reading the result of thinning the homogenate into a monolayer.

276-277: "we predicted that single impulses should yield transient oscillations ("bursts" or "ripples") as their spectral fingerprint" It has been known for many years that single TMS pulses administered to a healthy, awake brain produce long-lasting and complicated oscillations in the EEG, and indeed the complexity of the oscillations has been successfullly used as an index of patients' states of consciousness when they are non-verbal. Performing a spectrogram or wavelet analysis on a complicated series of peaks and valleys of variable timing will indeed produce a time-frequency plot that appears to have many brief bursts of oscillation at many different frequencies, so the predictions mentioned in this paragraph are not very impressive. However, there is no reason to expect that any of the apparent oscillations will turn out to be "genuine" oscillations, nor that they will be limited to the "bursty bands" previously identified in this manuscript. How does the authors' hypothesis lead them to expect that they will confined to "bursty bands"? Is this a reference to the above-quoted hypothesis that "bursts reflect rate-coding and response to change"? Is there an implicit assumption that a TMS pulse is in some way equivalent to the onset of a sensory stimulus? Is rate-coding a necessary part of any of those expectations or assumptions? There is a great deal left out here.

279-281: "Our TMS approach indeed provided causal evidence that in low-rhythmicity bands transient activity is reverberated, whereas sustained bands are dominated by ongoing oscillations." This is another very densely-packed, though-provoking sentence that urgently needs to be unpacked! First off, is it saying that the TMS experiments actually did confirm all the expectations and justify all the assumptions (whatever they were)? Second, is it saying that the authors' theory is that bursts of oscillation represent reverberatory activity (i.e. a delay-line-based oscillator) whereas sustained oscillations represent local microcircuit network activity (of which PING/ING would be a simple example)? And finally, does the authors' theory postulate that local network oscillations are necessarily sustained and cannot be responsible for brief bursts of oscillation? If so, why?

353-354: "Consistent with our hypothesis, single-pulse stimulations elicited reverberations in frequencies corresponding to the transient bands and beta1" How do the authors know that they are reverberations, and not successive stops in a long open-loop delay line (analogous to the brainstem auditory evoked potential)? The rest of this paragraph would be considerably more convincing if plots were shown of time-domain waveforms in addition to the plots of Figures 2F&G. Ideally, these should be a number of single-trial waveforms superimposed, each aligned at the onset of the stimulation. This would show not only the time course of the development (or lack) of oscillations, but also the degree to which the oscillations are temporally aligned to the individual pulses in the stimulation, and the degree to which the stimulation actually interfered with previously ongoing oscillations. The concluding sentence, "These results thus support our hypothesis..." would then be well-justified.

370-371: "two types of processes: change in response to inputs, manifested in oscillatory bursts which are transient, and maintenance of ongoing activity, reflected in sustained oscillations" This distinction makes excellent sense conceptually, and should be mentioned much earlier in the paper as part of a detailed description of the hypothesis under test.

372-373: "Our fourth prediction therefore suggests that patterns of activity within the rhythmicity architecture should respond to cognitive states, and particularly those related to inputs." The use of the word "therefore" seems to imply that there is some deductive logic to be followed here, but that is misleading and makes the sentence harder to understand. I believe the intended meaning would be better stated as, "Our fourth prediction is that patterns of activity within the rhythmicity architecture should respond to cognitive states, and particularly those related to inputs."

377-379: "If activity in sustained bands entails maintenance of ongoing activity as opposed to response to input, then rhythmicity levels in these bands should decrease in the presence of sensory input." The validity of this statement is far from

self-evident, and it takes some imagination to guess the assumptions that make it sensible. If we assume that there is some sort of trade-off between activity in sustained bands and activity in transient bands, then it makes sense. If we assume that changes in sensory input give rise to transient responses including transient oscillatory bursts, and we assume that transient bursts cannot be produced in sustained bands, then a rapidly changing sensory environment should produce more activity specifically in the transient bands (but possibly without changing the phase-consistency in those bands). However, if we assume that transient bursts can be induced in all bands, then we should see the phase-consistency go down in sustained bands, whereas the phase-consistency might already be close to its floor value in the transient bands. All of these assumptions and their logical relations to each other should be articulated early in the paper, before the presentation of Results.

386-388: "a significant increase in rhythmicity with eyes closed was observed also in the sustained band beta2 but was not observed in the transient band beta1" This is a very interesting observation! But, to be clear, does "eyes closed" here mean "rest with their eyes closed" (mentioned a few sentences earlier)? Is it possible that alpha indicates visual "idling" (a common interpretation), whereas beta2 indicates cognitive "idling"? And does this imply that the bursty beta1 band indicates cognitive activity, in particular changes in cognitive activity? These issues should all be discussed in the Discussion, but could be briefly alluded to here as part of the Results presentation.

Incidentally, this whole paragraph combined with Figure 1 show the ambiguity of the Greek letter band designations, which becomes problematic. Figures 1E&F show clearly how much overlap there is between neighboring bands, but beta1 and beta2 are shown in their customary ranges, typically specified as 13-22 Hz and 22-30 Hz. Figures 1G-J&L, however, show beta1 as 13-15 Hz and beta-2 as 18-22 Hz, with gamma1 occupying the range of 22-35 Hz that is labeled as "beta2" in Fig. 1E. This makes it hard to understand which bands are being referred to as "the sustained band beta2" and "the transient band beta1" in the present passage. Perhaps the 13-15 Hz band, which is color-coded blue for "transient", and the 18-22 Hz band which is color-coded green for "sustained", might better be referred to as "lower beta1" and "upper beta1", whereas the 22-35 Hz band marked "gamma1", which is color-coded blue for "transient", would usually be regarded as "beta2". ("Low gamma" usually means something like 30-50 or 40-60 Hz.) Or better yet, can we simply dump the Greek letters and denote bands by frequency? That would at least make it clear what frequency band a given sentence is referring to.

402-405: "We found that over the whole session, where in general the amount of input is low, HF significantly covaried with activity in sustained bands. In contrast, in response to a visual input, responding channels transiently increased their HF-LF covariance at a transient frequency band." This is another interesting and relevant statement, but where are the data? There should be a figure showing data that illustrate the statement.

417-422: "In line with these predictions, positively responding channels significantly increased..." Another very intriguing statement that works out very nicely. As always, a bit more connection to the data would be appreciated. Since the title of the original study was, "Distinct Ventral Stream and Prefrontal Cortex Representational Dynamics during Sustained Conscious Visual Perception", I assume there were EoG grids over lateral temporal cortex and lateral prefrontal cortex. Did both sets of grids contain both positively and negatively responding contacts? Was the fraction of each similar in both sets of grids? Were the contacts of each type uniformly distributed throughout the grids, or did they form clusters?

441-442: "Therefore, we hypothesized that medication would specifically affect the transient-band beta1, and not the sustained band, beta2, of PD patients." The Greek letters rear their ugly heads again! Does "beta2" mean 18-22 Hz here, 20-28 Hz, 22-35 Hz, or something else, e.g. the shaded region in Fig 3D occupying 16-20 Hz (which I would call "upper beta1", not just "beta1")? And what about the 20-28 Hz band, where there is white space clearly visible between the SEM shading, suggesting a p level slightly below 0.05 across the entire band? 20-28 Hz is frequently included in the computation of "beta power" in PD studies.

486-487: "Rhythmicity-based spectral segmentation reveals twice as many bands as previously described with two divergent modes of operation." Or possibly more; it seems unclear as to whether the typical "beta1" (~12-20 Hz) and "beta2" (~20-30 Hz) bands are each subdivided by this analysis into a transient lower half (12-16 and 20-24 Hz) and a sustained upper half (16-20 and 24-30 Hz), or whether in fact "beta1" (13-22 Hz in Fig. 1E or 13-15 Hz in Fig. 1G) is transient and "beta2" (20-28 Hz in Fig. 1E or 18-22 Hz in Fig 1G) is sustained. This murkiness regarding sustained and transient sub-bands in the 12-30 Hz range needs to be clarified, or at least discussed together with early reports of transient bursts of beta (~18 Hz in rat, Howe et al. 2011 Proc Natl Acad Sci U S A. 2011 Oct 4;108(40):16801-6; 16-20 Hz in monkey, Feingold et al. 2015, Proc Natl Acad Sci U S A. 2015 Nov 3;112(44):13687-92).

487-488: "This indicates that there is room for both rate- and phase-codes with important complementary roles" Same unsupported conflation objected to above.

565: Is LAVI based on a fixed interval independent of frequency, or a fixed phase lag for a given frequency?

577-578: "The time frequency representation was calculated by multiplying the Fourier transforms of the data and the wavelet of each frequency, and taking the inverse transform of the product." Clearly this cannot mean multiplying a time-domain signal by a frequency-domain signal, so presumably the wavelet was also Fourier transformed. It is true that multiplying in the freq domain maps onto convolution in the time domain, which is how the wavelet transform is computed in the time domain, but a Fourier transform implies a fixed time window in the time domain and a time-independent spectrum in the frequency domain. The mechanics of how these operations were adapted using moving time windows of various widths is completely unspecified.

598: the value of n must be specified.

606-607: "shuffling the data" Why? How?

609-611: "assigning the values of the original data to the adjusted time-series based on ranking (the highest value of the surrogate gets the highest value of the original data and so forth)" The confusion intensifies. Point by point? Window by window? Frequency by frequency??

635: "Spectral parameterization (specparam, formerly fooof" What was the actual frequency band over which specparam was applied? This is important, because the 1/f model is only approximately correct and does not work very well when it is applied over a frequency band that spans more than a factor of 10 or so.

641-643: "we decomposed the signal using an array of Butterworth bandpass filters (order = 3), centred at frequencies 3 to 100 Hz in steps of 1 Hz and bandwidth of 1 Hz," Really??? At 100 Hz, a 1 Hz bandwidth is extremely high-Q and resonant, and a 1 Hz passband implies that temporal resolution is limited to about 0.5 s at all frequencies. Aren't the bursts being simulated of shorter duration than 0.5 s?

646-5647: "we first “notch filtered” the activity in 14-16 Hz by multiplying the filtered signal at this frequency by 0.5." Hard to understand. Are the authors saying that they made FFTs of 0.5 sec long windows so that a single frequency point occupies a 2 Hz band, and multiplied that single amplitude by 0.5? Then the authors would have to transform back to time domain to measure burst frequency. Needs step-by-step explanation.

668: "can be explained as an (artefactual) effect of the peak in alpha" This must have something to do with the comment that creating a simulated peak results in desynchronized sidebands, but it's unclear what.

764-765: "we computed the difference between the phase in the time-point of interest (0), one cycle beforehand (-1) and one cycle after (1)." Better description needed! By definition, there is zero phase difference between a time point of interest and the time point one cycle beforehand.

797-798: "To estimate the Low-Frequency (LF) activity, we computed the time-frequency representation of the raw data using complex Morlet wavelets." Given that HF starts at 70 Hz, I presume LF means everything below 70 Hz? Wait, top Morlet freq is 45 Hz... Why use different methods for HF and LF? And what bands are meant by LF and HF?

815-817: "Thus, frequencies in the original data with LAVI values higher than the maximal value of the surrogate distribution were defined as having significantly (p=1/20=0.05) high rhythmicity." Are the authors sure this is equivalent to the more usual method of bootstrapping, in which a large number of surrogates are analyzed and the top and bottom 2.5% of values are thrown out to find the p=0.05 levels? I suspect they are probably not equivalent. Do other investigators use the authors' method?

828: "Bayes Factor (BF) t-tests" What's the advantage of this lesser-known method over a traditional t-test?

Supp 47-49: "The wider the Wavelet, the higher the assessed rhythmicity (fig. S5D)." A wide wavelet forces the phase to be strongly correlated over a wide time lag, so a distinction must be made between that analysis nartifact and the data-driven correlation determined by the physiology.

Supp 48: "allows statistical significance inference of each band (see ABBA above). " ABBA is not described "above", it is in the main text.

(Remarks on code availability)
There are some remarks in passing about certain code functions that are available on Github, but there does not appear to be any upload of code or data used to produce the figures.

Version 1:

Reviewer comments:

Reviewer #1

(Remarks to the Author)
We appreciate the effort in restructuring the paper for this second submission. Concepts, hypothesis and predictions are clearer; each section responds to one individual question and individual figures focus on testing the specific predictions of the study. We values the single subject data, as well as the population summaries, which also make the claims more interpretable. Separating the phase-coding/rate-coding concepts (moved to discussion) from the sustained/transient nature of oscillations allows for a more direct test of hypothesis, leaving the possible interpretations for the discussion session, which was also considerably extended.
We believe the main claim of the paper, i.e. the idea of including ‘rhythmicity’ as a relevant feature in spectral analyses provides a new dimension in which we could understand neural activity, and the coexistence of low-rhythmicity with high-rhythmicity bands is a highly systematic result across populations, datasets and recording methods.

The ABBA algorithm helps connecting sustained/transient frequency bands to the canonical frequency bands defined on the frequency dimension. It is useful to detect the significance of high-vs-low rhythmicity by comparing them to the noise ribbon. However, in my opinion, inter-subject frequency variability complicates the generalization of specific frequency band as sustained or transient (e.g. gamma is transient, beta2 sustained, beta1 transient). The data clearly supports the existence of high-and-low rhythmicity bands (both in individual subjects and across populations/recordings), but the dynamics of each frequency band seem to be more subject and dataset dependent.
Finally, the number of bands detected, as well as the similar profile across sexes, the age differences and pathological differences seem clearer as presented now.

Specific comments:
1. From Low- and high-rhythmicity bands show distinct burst dynamics, it is clear that the sustained and transient bands are significantly different. However, it is not very clear why in lines 211-214 there is the idea that sustained corresponds to more than 3 cycles and transient around 1 cycle, when in later analysis results seem to differ (e.g. 3.6 vs. 2.9 cycles) – and it changes based on the method used for duration detection. Also, in simulated data it seems that bursts below 4 cycles correspond to significantly low rhythmicity, which would correspond to sustained bands as well, and longer than 6 to sustained, which does not occur on real data. It is possible that results from simulated data and real data can't be directly comparable, and we agree that the 1/f phase-randomization test is important, but not sure what would be the message we extract from the simulations with generated bursts -if we assume that the cycle numbers can't be directly linked to real data.
2. From the LAVI calculation (used in Figure 4A), in the preprocessing EEG methods it is discussed that only a few channels are rejected from the analysis, but then that electrode Cz is used for rhythmicity and burst analysis (line 713). Then, are the values obtained from that unique electrode, or are they the average across the different scalp electrodes? If only calculated based on Cz, did you see any gradient/differences across the different contacts? Should we expect the same results from different locations?
3. In Figure 5, we understand the need for the different scales to show the two results, but especially in C, it is very misleading that values of yellow are way higher when in the full graph they are smaller, It could be useful to maybe set the 0.4 (which belongs to both graphs at the same hight (similar to A).
4. Furthermore, in Fig. 5, is the EEG source localized to the same site as the LFP? If not, would one at all expect them to contain the same signals so that a direct comparison is possible?
5. From Dual rhythmic modes and input processing, the assumption is that positively responding channels are engaged in the process of the current stimuli and negatively responding channels are engaged in something else, but negatively responding could as well be a response to the stimuli, not necessarily the interruption of something else.
6. Figure 7B, when comparing left to right shows another example of the differently defined frequency bands across contacts (comparing the blue/green bands), e.g. beta2-R aligns better with beta1-L and gamma1-R is in between of gamma1-L and beta2-L. The result of high rhythmicity bands showing a negative change in rhythmicity (and lower rhythmicity reverse) is interesting, yet not very clean in the left plot. The beta and gamma bands are not very clearly dissociated between them and it is not very clear to which band is the increased WTPL associated with, or whether the increase we see corresponds to the gamma band defined by the ABBA.
7. Similarly, how different contacts yield different frequency bands, could it be possible that when selecting the whole section vs. the 0-0.2s the bands would also change? It is not clear that it is possible to run the ABBA algorithm on a small subset of data (like the 0-0.2s) but could some differences in Fig7C be associated with that?
8. In Figure 7C (left), it seems clear that in the increasing population for the visual input time, there is a clear peak in the transient band, and for both lines there is a decrease in the alpha band. However, it is not very clear if in the whole session the peak is really at theta or just at an in-between of the bands, pointing more towards a reduce in the power than a real change from sustained (gray) to transient (purple).
9. Furthermore, 7C (right) is not very convincing as supporting your concept. The increase in ACOV just after visual stimulation is covering both the sustained alpha and the transient beta1, and idem for the sustained theta and the transient gamma1. Thus both transient and sustained bands are modified by stimulus onset, and not in a logic way that would assign roles to transient vs. sustained, but rather as theta/gamma going hand in hand (as often seen in literature) and alpha/beta going hand in hand, as also often seen in literature. In short, given all these issues with this section, we would suggest removing it completely, and instead making a separate, more detailed study of this for another paper.
10. Furthermore L459: here you say HF-LF covariance, in the figure legend you say LF-HF covariance or ACOV... please be consistent with naming, the paper is very dense and many abbreviations and concepts that are not used consistently.
11. Then for the TMS section, figure 8C is nice as an extension to highlight the importance of the finding in 8D, which seems the most convincing, clear and interesting part of the TMS. 8F is still complex, in particular for the arrhythmic effects. Again, we propose to remove the rhythmic/arrhythmic part of the TMS, and leave this to a separate study, and potentially only keep the single-pulse TMS result (Fig. 8 B-D)?
12. I am not at all an expert on TMS, but I can understand the hypothesis of sustained oscillations enhanced by rhythmic pulses and disrupted by arrhythmic ones. I was just surprised that transient oscillations would also be enhanced (increased rhythmicity) by rhythmic stimulation (Figure 8F-middle). Should this be expected? So, in other words, transient bursts would become more rhythmic by any external stimulation, no matter if it is rhythmic or arrhythmic?
13. On the discussion, similar comment that before (lines 616-618), I find it difficult to generalize the sustained/transient to specific frequency bands, since we have seen than recording modality, species and different subjects within populations have different frequency bands.
14. In the Figure 3 caption end you say: "Dotted lines mark troughs; arrows indicate phase shifts, likely due to neighbouring high-rhythmicity bands." Would this mean that the high rhythmicity band makes the low rhythmicity band next to it look seemingly low? i.e. is beta1 'obliged' to be low in your analysis, as it is affected by the neighbouring alpha band which has higher power and thus distorts the beta1 signal? This is again taken up in the discussion, where you say that L633-636 : "We observed that high-rhythmicity bands can suppress the emergence of oscillations in adjacent bands via phase interference (Fig. 3A). Specifically, ongoing oscillatory activity in a dominant high-rhythmicity band may curtail the development of nearby

rhythms through phase-based interactions (i.e., interference).” Might this only be a problem of your measurement, but not for the neurons engaged in the two neighbouring rhythms? For that one would possibly need intracellular recordings from neurons engaged in the different rhythms, which is of course out of scope for this paper… so maybe best to not speculate too much on this? But it indeed makes one question whether beta1 is somehow dictated a transient label in your analysis, just from happening to be next to alpha?
15. In line 350 you say sustained bands characterized by long-lasting oscillations and transient bands dominated by brief bursts, but even if the difference between 3.6 and 2.9 might be significant, it remains a tiny effect… also with the other measures you test, there will be a great overlap in duration of transient band vs. sustained band bursts, and none of them seem to be ‘long-lasting’? Also on line 553 you say sustained and bursty, please be consistent with naming.

Sincerely, Laura Lopez-Galdo and Bjørg Kilavik

(Remarks on code availability)

Reviewer #2

(Remarks to the Author)
I co-reviewed this manuscript with one of the reviewers who provided the listed reports. This is part of the Nature Communications initiative to facilitate training in peer review and to provide appropriate recognition for Early Career Researchers who co-review manuscripts.

(Remarks on code availability)

Reviewer #3

(Remarks to the Author)
The revised manuscript is head and shoulders above the first draft. The greatly expanded set of figures is an enormous improvement. All of this reviewer's comments have been addressed well, many truly excellently. The goal mentioned in the authors' response to reviewer comment 3.18 of making the paper "akin to a well-prepared gel, where distinct bands can be clearly resolved and interpreted" has truly been achieved. I feel it is ready for publication with minor corrections.

There is one recurring typographical issue that should be corrected before the paper goes to press. Typographically, the symbol for "approximately" is "≈" (Unicode 247 = 0xF7), not "±" (Unicode 177 = 0xB1) which means "plus or minus" and can be very misleading.

There are also some issues that could optionally be corrected to make the paper more understandable, but they are no reason to hold it back from publication. They are described in the following.

Assuming that the phrase "the maintenance of ongoing activity" is meant to include the maintenance of lack of activity as well, a slight modification of the way it is initially spelled out might help. Some possible alternatives would include "the maintenance of current state", "the maintenance of ongoing activity (or lack thereof)", etc.

The many instances of the abbreviation "HF", defined as "High-Frequency (HF) activity ... as reported in 29", are slightly confusing, especially on a quick read-through. Ref. 29 states: "high-frequency activity (HFA, 70-150 Hz)". This is very clear, and it would help the clarity of the current paper simply to quote it, and then use the abbreviation "HFA" as it is used in ref. 29.

Legend for Fig. 7A, "Panels B-C are also divided to increased (left) and decreased (right) units": if the recordings are ECoG, they are almost certainly massively multiunit, and should not be referred to as "units". Also, the tail end of the legend for Fig. 7A is not a good place to specify that all of the panels show "increased" channels on the left and "decreased" channels on the right; better to put this information before the panel-by-panel descriptions start.

Fig. 8B: beta1 filter
There does not seem to be a specification anywhere in the paper of what "filtered in the beta1 band" means. It appears to be filtered with a zero phase shift filter (e.g. Matlab's 'filtfilt' function), and the waveform looks quite reasonable, but filter type (e.g. "4th-order Butterworth") and passband should be specified somewhere.

Figs. 8C-D: This set of results is now easily understandable, and the question that comes immediately to mind is, "Are these changes statistically significant?" Legend states: "Trace plotted to the right of the panel depicts the mean power during the first 200 ms after TMS (C) or baseline rhythmicity during the [-1 -0.5] s baseline window (D)." However, no scales are given for the traces, and there is no statement as to what the black outlines in the pseudocolor plots mean. Are they outlines of statistically significant regions? At what p level? The CLs around the traces "to the right" would give some hints to answer these questions if there were a scale bar and an explanation as to what kind of CLs they are (95% Confidence? Plus/minus SEM? Plus/minus SD?).

New lines 632-646 present some interesting ideas, but it is hard to tease apart the biology and the analysis technology. On the one hand, the band-stopped signals are a technical manipulation that shows how strong oscillations in neighboring

bands could potentially confuse the LAVI algorithm. Rather than disrupting oscillations in neighboring bands, it masks them by creating peaks and valleys that are similar to those that may occur in-band. On the other hand, biological mechanisms that are sensitive to rhythm could similarly get confused. It would be good to point this out so that readers don't dismiss the point as "just a technical artifact".

Fig. 3A: the gray lines are very hard to see, especially in the averages at the bottom.

Signed,
Daniel J. Gibson

(Remarks on code availability)
I have only very briefly glanced at the code, primarily to make sure that it is easily accessible as stated on Github. It appears to be well organized and clearly documented, but I have not analyzed it in any detail.

Version 2:

Reviewer comments:

Reviewer #1

(Remarks to the Author)
The authors did a great effort in answering all our remaining concerns, and improving the clarity of the manuscript and the large amount of work, in a satisfactory manner. We have no further questions at this point.

Sincerely, Laura Lopez-Galdo and Bjørg Kilavik

(Remarks on code availability)

Reviewer #2

(Remarks to the Author)
I co-reviewed this manuscript with one of the reviewers who provided the listed reports. This is part of the Nature Communications initiative to facilitate training in peer review and to provide appropriate recognition for Early Career Researchers who co-review manuscripts.

(Remarks on code availability)

Reviewer #3

(Remarks to the Author)
Excellent paper! All my comments have been addressed with changes that are spot-on.

(Remarks on code availability)

Open Access This Peer Review File is licensed under a Creative Commons Attribution 4.0 International License, which permits use, sharing, adaptation, distribution and reproduction in any medium or format, as long as you give appropriate credit to the original author(s) and the source, provide a link to the Creative Commons license, and indicate if changes were made.
In cases where reviewers are anonymous, credit should be given to 'Anonymous Referee' and the source.
The images or other third party material in this Peer Review File are included in the article's Creative Commons license, unless indicated otherwise in a credit line to the material. If material is not included in the article's Creative Commons license and your intended use is not permitted by statutory regulation or exceeds the permitted use, you will need to obtain permission directly from the copyright holder.
To view a copy of this license, visit https://creativecommons.org/licenses/by/4.0/

Please find our point by point response to the reviewers' comments below in green text. To facilitate the tracking of amendments to the manuscript text, we cite amended text in this document and refer to the line numbers in the manuscript.

==

REVIEWER COMMENTS

Reviewer #1 (Remarks to the Author):

In this paper, Karvat and colleagues studied brain rhythmic activity, incorporating several existing datasets in their analysis from humans and rodents, obtained with different recording modalities, and one novel dataset they recorded in their lab combining EEG/TMS. They perform a comparative study across species, modalities, behavioral tasks/states and age, with the aim to determine if there is a rhythmic architecture with distinct modes that is comparable across the different datasets.
We consider this topic of high interest, and the authors addressed this question from a novel and interesting point of view. During the last decade more and more studies have moved away from considering brain rhythms as sustained oscillations, and embraced the notion of transient rhythmic bursts, which can best be studied at the single trial level. We still need a good description and understanding of brain rhythms in this novel perspective of transient bursts, and this study can provide a valuable contribution to this.
We appreciate the effort to include a large variety of datasets, with the aim of determining the universal aspect of their findings, across modalities, species etc. This is a strength of your paper.

Reviewer's comment 1.01:
However, we miss that you go in depth in the individual datasets, in particular in individual subjects. While developing general principles based on common findings across large datasets is very valuable, and useful to build models aimed to gain mechanistic understanding, this also washes out any details, somewhat hindering this mechanistic understanding.

For instance, on the one hand, to which extent can individual subjects have all the transient and sustained bands you describe (which is not clear in Fig. 1 E/F).

Response: we sincerely thank the reviewer for their detailed and constructive comments, which have significantly helped us improve the clarity and depth of our findings.

In response to this first point, we appreciate the emphasis on examining individual datasets more closely. We have now expanded our analysis to include several examples at the individual-subject level (Fig. 1F–H, Fig. 2B–C), and we explore

individual datasets in greater depth throughout the revised manuscript (Fig. 2D–E, Fig. 4 [3 datasets], Fig. 5 [2 datasets], Fig. 6 [2 datasets], Fig. 7B, and Fig. 8D).

To address the specific question regarding the extent to which individual subjects exhibit all the transient and sustained bands, we have added new summary data in Fig. 2E, as well as in Fig. 4. In particular, Fig. 4C, 4F, and 4I quantify the number of bands observed per subject across the EEG datasets. For example, Fig. 4C shows the distribution of band counts per individual, illustrating the variability and prevalence of these features across participants:

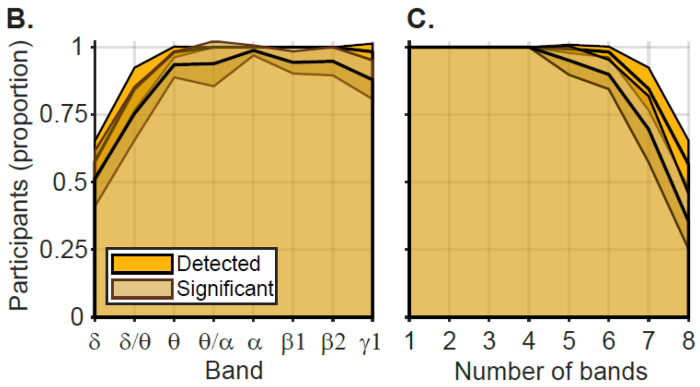

Reviewer’s comment 1.02:
On the other hand to which degree can one specific band be both transient and sustained within the same subject, depending e.g. on task epoch or behavioral state. In the analysis all data is collapsed when estimating rhythmicity for each frequency, such that within one subject each frequency only can get one label.

Response: We thank the reviewer for raising the important point regarding the potential for a specific band to be both transient and sustained within the same subject. As noted, our analysis estimates rhythmicity per frequency across the entire session, resulting in each frequency being assigned a single rhythmicity value.

In comment 1.10, the reviewer refers to Kilavik et al. (2012), where beta frequency and power varied with task stage. This raises the possibility that a given frequency might exhibit sustained activity in one cognitive state and transient activity in another.

Our findings suggest that this is unlikely. Both the rhythmicity per frequency and the frequency ranges of detected bands appear to be stable across states. To address this directly, we added new analyses and figures:

In Fig. 2, we show that while behavioural states (i.e., eyes open vs eyes closed) influence rhythmicity magnitude, the automatically detected band frequencies remain stable. In Fig. 3F, we demonstrate that approximately 80% of burst frequencies fall within the peak’s band, indicating limited cross-band “slip-over.”

We now explicitly mention this in the main text (line 197-199):

> "Notably, the frequency ranges of bands identified by ABBA, as reflecting high or low rhythmicity, remained stable across states ($t_{295} = 1.4$, $p = 0.16$, $BF_{10} = 0.17$), underscoring the robustness of the spectral architecture."

Finally, we note that in the time-resolved analysis of rhythmicity (WTPL; Figs. 7 and 8), dynamics in rhythmicity magnitude within bands become apparent only after baseline normalization, as baseline activity—a proxy for LAVI—remains highly stable and different between bands. This indicates that differences and separations between bands are overwhelmingly greater than within-band dynamics.

Reviewer's comment 1.03:

It is difficult to understand Fig. 1E/F, but one way to interpret it is that only a fraction of subjects actually present anyone of the sustained/transient bands they describe, thus the general principles seem not systematically observed in individual datasets and subjects? Otherwise, how to interpret the fractions for each frequency? This needs clarification, or maybe a different way to visualize.

Response: We thank the reviewer for highlighting the ambiguity in the original visualisation of Fig. 1E/F. In response, we have revised this figure and now present the data across two separate figures (Figs. 2 and 4) to improve clarity.

Specifically, instead of the previous population distributions, we now first show a forest plot of all detected bands for 3 individual participants alongside their raw LAVI rhythmicity profiles (Fig. 2B/C). This is followed by a population-level summary of both LAVI and ABBA (Fig. 2D/E), where distributions are presented with means, interquartile ranges, and 95% confidence intervals.

To directly address the concern that "only a fraction of subjects actually present any one of the sustained/transient bands," we added a new analysis summarising the proportion of participants and electrodes in which each band was detected, and how many of those detections were statistically significant (Fig. 4B, 4E, 4H). Additionally, we now report the proportion of participants/electrodes presenting at least x bands. For example, at least 6 bands were detected in 98% of EEG participants, at least 7 in 85%, and all 8 bands in 57%.

We also clarify this point in the main text (line 289-295):

> "Using ABBA, we consistently detected the canonical sustained bands—θ, α, and β2—as well as the transient bands—θ/α, β1, and γ1—in nearly all participants (Fig. 4B, 4E, 4H). Detection rates were lower for δ and δ/θ, likely due to our choice of 3 Hz as the lowest frequency analysed, which might not capture δ and δ/θ rhythmicity,

> resulting in a noisy estimate at these frequencies (i.e., the actual peaks may be in lower frequencies than measured). Importantly, for most participants and electrodes, at least 6 out of 8 bands were detected (Fig. 4C, 4F, 4I)."

Reviewer's comment 1.04:

Furthermore, if one would look at a specific frequency, such as e.g. 6Hz, of the fraction of subjects that have significantly sustained or transient in this epoch, it seems to split 50/50 between the two categories?

Response: We thank the reviewer for this insightful observation. Indeed, specific frequencies—such as 6 Hz—can be classified as sustained in some subjects and transient in others. This variability is a key point that underscores the importance of subject-specific spectral categorisation, which is one of the central contributions of LAVI and the rhythmic architecture framework.

Following this comment, we now include an explicit example in Fig. 2C and clarify this point in the main text (line 152-155):

> "Importantly, within the universal framework, individual subjects showed considerable variability, with some frequencies classified as high- or low-rhythmicity depending on the subject (Fig. 2D–E and fig. S2G). This variability underscores the need for an objective, single-subject band-detection framework."

Notably (and relevant also to comments 1.6 and 1.11 below), when two LAVI peaks of similar amplitude occur in close proximity (e.g., 24 Hz and 28 Hz in Figure 2A), and the trough between them does not go below the noise ribbon, the ABBA method will indeed prioritise the stronger one, potentially collapsing them into a single band. This is a deliberate design choice to avoid over-segmentation in noisy spectra. However, when the peaks are sufficiently distinct in frequency or rhythmicity magnitude (e.g., 7.5 Hz and 12 Hz in Figure 2A), they are typically resolved as separate bands.

Reviewer's comment 1.05:

Thus, are you claiming that the exact bands that you find universally have a 'transient' or 'rhythmic' nature, so that for instance beta1 is in general a transient band and beta2 is a sustained band? In this case, it is a bit disturbing that you set a very broad range of the alpha band that you use to label the other bands, and that the method will assign the names of the frequency bands always relative to the "standard" alpha peak.

Response: We thank the reviewer for raising this point. Indeed, we do find that certain bands tend to exhibit consistent characteristics across subjects—for example, beta1 is generally a transient band, while beta2 is typically sustained.

However, we would like to clarify a possible misunderstanding regarding the ABBA algorithm. We agree that 6-14 Hz is a rather broad range. But we do not assume that the alpha band has this range. Rather, we *search* within a broad frequency window (6–14 Hz) because alpha is reliably (~100% of datasets, Figure 4) found within this range. Once the alpha peak is identified, the actual bands are defined more narrowly and individually for each subject, and by statistically comparing the rhythmicity of those bands to a surrogate distribution. This can be seen in Fig. 2B–C for individual examples, and in Figs. 2D–E, 5B/D, 6B/D, 7B-C, and 8D for population-level data.

This process is described in the Methods section (lines 817–821):

> "Since most subjects have a clear peak in the rhythmicity profile at the alpha band, the peak between 6 to 14 Hz can be used as an anchor to automatically allocate bands identity: the frequency with highest rhythmicity between 6 and 14 Hz is taken as alpha, the trough following alpha, as the frequency increases (i.e., to the right of alpha) is beta1, the next peak is beta2, and so forth, and also for frequencies lower than alpha."

and to ensure this point is also clear in the main text, we have added the following clarification (lines 146–152):

> "…we consistently observed a dominant peak within the 6-14 Hz range, aligned with the traditional 'alpha' band, allowing for automatic annotation of band labels. We label this peak as 'alpha' (α); the subsequent trough is labelled 'beta1' (β1), followed by the next peak as 'beta2' (β2), and the following trough as 'gamma1' (γ1). Similarly, the trough preceding alpha is labelled as 'theta/alpha' (θ/α), the adjacent lower-frequency peak as theta (θ), the next lower trough as 'delta/theta' (δ/θ) and the lowest peak considered in this manuscript is labelled delta (δ) (see Fig. 2A)."

Reviewer's comment 1.06:

In Fig1-L it is stated that when the dataset was published the band was considered Theta, yet here the band would be assigned to Alpha based on the ABBA algorithm. How can we understand the claims directed to specific frequency bands? In this case the effect of theta would be hidden inside the alpha behavior… Such a strong claim, of e.g. beta1 being transient and beta2 sustained, seems indeed to be what you aim for, given your discussion on page 10. Or are you more loosely claiming that there will be transient and sustained/rhythmic bands, and that the sustained ones will dominate the power spectrum, but that which bands are either or can change across subjects and or datasets?

Response: we thank the reviewer for raising this conceptual point. Indeed, we are proposing that certain bands—such as beta1 and beta2—tend to exhibit consistent characteristics across subjects, with beta1 generally being transient and beta2 sustained. However, we also acknowledge that the precise frequency ranges of these bands can vary across individuals and datasets.

The reviewer is correct in noting that, in Fig. 1L, a band originally labelled as 'theta' in the published dataset is identified as 'alpha' by ABBA. This highlights a key motivation for our approach: traditional band labels, when based only on frequency, can be ambiguous leading to misguided comparisons across species and contexts. ABBA addresses this by assigning band identities based on the subject-specific rhythmicity profile, rather than fixed frequency boundaries.

Notably, this relabelling of bands does not necessarily coincide in frequency with the bands traditionally defined by that name. For example, we do not suggest that every instance in the literature where researchers referred to beta1 (based on frequency) corresponds to our transient beta1 band (defined by rhythmicity). Rather, our aim is to encourage researchers to revisit existing frequency-based findings through the lens of rhythmicity.

To clarify this point, we have added the following paragraph to the main text (line 325-337):

> "Furthermore, the invasive recordings from patients' hippocampi enabled a cross-species comparison of rhythmic architecture with rats. A longstanding debate concerns whether human alpha oscillations (8–12 Hz [3]) are functionally analogous to rodent theta (4–12 Hz [34]). While some argue these rhythms reflect similar mechanisms despite frequency differences [35,36], others question whether rodents exhibit alpha at all, noting their hippocampal activity is dominated by theta [37,38]. To address this and test whether the rhythmicity architecture generalizes across species, we analyzed hippocampal recordings from 11 rats (Dataset XII). As in humans, both sustained and transient bands from θ to γ1 were detected in nearly all rats (100% for θ–γ1; significant in 100% for θ/α, α, and β1; 90% for β2; 82% for θ and γ1). Although rhythmicity magnitudes were higher in rats (note scale in Fig. 5C), band frequency distributions were strongly conserved across species (BF_{01} = 10.99, Fig. 5D). Notably, the dominant peak in rats was around 8 Hz—labelled 'theta' in the original publication of dataset XII [32], but identified as 'alpha' by ABBA—suggesting that rodent theta reported in the literature may correspond to human alpha, as the most prominent rhythmic band below ~15 Hz."

This example illustrates that while the *labels* may shift, the underlying rhythmic structure is preserved, and ABBA provides a principled way to resolve such ambiguities in a data-driven, assumption-free approach.

Reviewer's comment 1.07:

Furthermore, you link transient and sustained bands to rate code and phase code, respectively. However, you never seem to actually test phase coding. Since you find that transient bands correspond to <4 cycles and sustained/rhythmic as >6 cycles (P8), both transient and sustained bands have 'bursts', that mainly differ in duration. However, even a few cycles in a transient burst to some extent suggest phase coding, and your link with high-frequency was an amplitude co-variance analysis? Do you need to explicitly make the link to phase/rate coding? If so, then this should maybe be tested more directly?

Response: we thank the reviewer for this thoughtful observation. We agree that the transient–sustained distinction can be considered independently of the rate–phase coding framework. To reflect this, we have revised the manuscript by moving the discussion of rate and phase coding to the *Discussion* section, where it is presented as a possible interpretation rather than a central claim. This adjustment ensures that our conclusions remain grounded in the data, while still allowing for broader theoretical implications to be considered.

Reviewer's comment 1.08:

It is also somewhat unclear why the sustained bands dominate the power spectrum, since the shorter-duration transient bursts to some extent 'compensate' by being more frequent (Fig. 2A)? Do the sustained bands also reflect bursts with higher instantaneous amplitude, or can transient bursts (<4 cycles) have similar amplitude as sustained bursts (>6 cycles)?

Response: we thank the reviewer for raising this point. As noted, the higher rate of transient bursts compensates for their shorter duration. Indeed, as shown in the newly added Fig. 3E, the overall time spent above threshold (termed 'occupancy') is comparable between transient and sustained bands.

However, bursts in sustained bands tend to exhibit slighlty higher peak power than in transient bands, even after normalising to baseline power to account for 1/f scaling (Fig. 3F). This likely contributes to their dominance in the power spectrum. This point is reflected in the revised results section (line 220-221):

> "with high-rhythmicity bursts showing slightly higher normalized amplitudes (Fig. 3F)"

We also mention this point in the *Discussion* to highlight it as an important direction for future work (line 632-646):

> "A further promising direction for future research concerns the interactions between neighbouring frequency bands. We observed that high-rhythmicity bands can suppress the emergence of oscillations in adjacent bands via phase interference (Fig. 3A).

> Specifically, ongoing oscillatory activity in a dominant high-rhythmicity band may curtail the development of nearby rhythms through phase-based interactions (i.e., interference). This interpretation is supported by our finding that removing the high-rhythmicity band using a stop-band filter abolished the observed phase shift (Fig. 3A), consistent with LAVI's sensitivity to phase differences. This interpretation, together with the dominance of high-rhythmicity events (higher power even when normalized to compensate the 1/f slope, Fig. 3F) also carries intriguing implications for the broader operational principles of the brain. It raises the possibility that high-rhythmicity bands may serve to establish dedicated "quiet" spectral zones—frequency ranges held in readiness for incoming input. In such a scenario, an external or internal input would produce a brief, ephemeral response, which is then rapidly curtailed by the surrounding rhythmic context. This mechanism would not only support a top-down role for sustained oscillations but also suggest that such control is not purely inhibitory. Rather, it may enhance contrast and selectivity by dynamically shaping the spectral landscape to prioritise response-relevant signals"

Reviewer's comment 1.09:

Then, to go into more detail to understand the underlying mechanisms, to which degree would frequency drift within long bursts tend to limit the LAVI measure, making the signal less rhythmic, and then defined as more transient? For instance, Atallah and Scanziani (2009) showed that the frequency of gamma bursts can change from cycle to cycle in the rodent hippocampus (interestingly linked to amplitude in the preceding cycle). Frequency drift within individual burst can also be observed in data from the macaque prefrontal cortex (e.g. Lundqvist et al. 2016, Fig. 4a), even if not necessarily highlighted by the authors.

Response: The reviewer makes here an interesting suggestion; As noted, Atallah and Scanziani (2009) demonstrated that the momentary frequency of gamma bursts can vary from cycle to cycle, with frequency shifts linked to the amplitude of preceding peaks. Similarly, Fig. 4a of Lundqvist et al. (2016) shows that frequency drift can occur within individual bursts in macaque prefrontal cortex. To assess whether such frequency drift might limit the LAVI measure—potentially leading to lower rhythmicity estimates and classification as more transient—we conducted two complementary analyses: one empirical and one simulated.

In the empirical analysis, we examined each burst from each participant in Dataset I. For each time point within a burst, we measured the peak frequency and calculated the proportion of time that the frequency remained within the designated band. As shown in Fig. 3G (and reproduced below for convenience), over 80% of peak frequencies during bursts were confined within the band boundaries, both for sustained and transient bands. This suggests that while frequency drift might occur, the drift is small and typically remains within the defined band. To best capture the

impact of such small drifts we simulate the drifts (see below) and conclude that they likely do not substantially reduce rhythmicity.

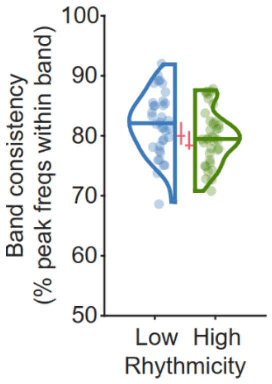

We now include this point in the main text (line 220-223):

> "Moreover, although instantaneous frequencies can drift within bursts [17,23] (fig. S4), in both modes over 80% of peak frequencies during bursts were confined within the LAVI-defined frequency band (Fig. 3G)."

To systematically test the effect of frequency drift on LAVI, we also ran simulations. We generated 10-cycle, 15 Hz bursts embedded in pink noise, and introduced linear frequency drifts across the burst duration (from 15 + drift/2 to 15 – drift/2 Hz). As the reviewer anticipated, frequency drift does reduce rhythmicity. However, rhythmicity remained above noise level for drifts up to 5 Hz (i.e. ~33% of the original frequency, and as beta1 is often found in the 14-19 Hz, 84% of the band), as shown in Supplementary Fig. 4.

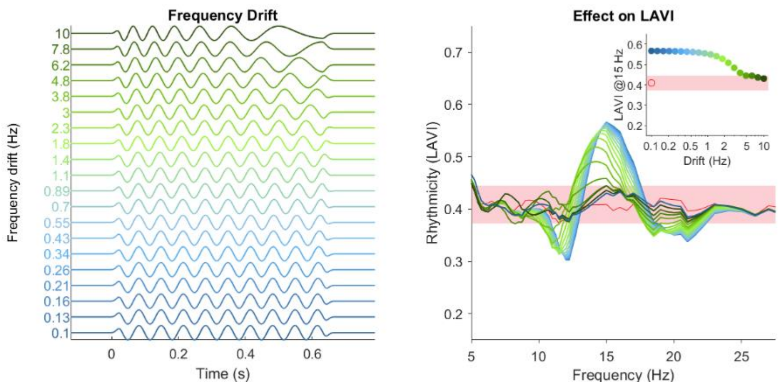

These results suggest that while frequency drift can influence rhythmicity estimates, LAVI remains robust to moderate within-burst frequency variation.

Reviewer's comment 1.10:

Furthermore, Kilavik et al. 2012 showed that in the macaque motor cortex both low and high beta (i.e. beta1 and beta 2) are present in delays and in epochs immediately following an informative visual cue (more transient and with slightly increased frequency and lower power after visual cues). Thus, both beta1 and beta2 can be linked with epochs of a task typically associated with sustained and transient (evoked) activity in the same subject. Although they did not study bursts, this is linked to the questions above, whether one band can be transient in one context and rhythmic/sustained in another?

Response: We are grateful to the reviewer for highlighting the study by Kilavik et al. (2012), which indeed lends further support to our interpretation. In this work, two macaque monkeys performed a centre-out reaching task involving two delay periods. The authors reported increased beta power in a broad frequency range (12–40 Hz), with one monkey showing a peak around 20 Hz (low-beta), and the other exhibiting peaks around both 18 Hz (low-beta) and 30 Hz (high-beta). Notably, during periods of expectancy (E1 and E3), the momentary peak frequency was lower and power higher, whereas following the informative visual cue (E2), the frequency increased by 4-6 Hz and power decreased—suggesting a shift in the spectral profile associated with task demands.

We thank the reviewer for drawing our attention to this paper, which we believe aligns well with our framework. Specifically, we interpret the delay and anticipation periods (E1 and E3) as reflecting sustained oscillatory activity, likely falling within high-rhythmicity bands. In contrast, the cue-evoked epoch (E2), which reflects a rapid response to external input, is more consistent with transient, burst-like activity in low-rhythmicity bands. The frequency difference (4-6 Hz) fits well to differences often found between beta1 and beta2. Although Kilavik et al. did not explicitly analyse bursts, their findings support the idea that distinct frequency sub-bands within the beta range may correspond to different rhythmicity regimes and functional roles.

In our revised Discussion (line 625-631), we now explicitly refer to this study:

> "Notably, one study [11] demonstrated that both transient and sustained beta responses can co-occur within individuals, varying with task stage—transient responses following informative cues, and sustained responses during anticipatory periods. These were also spectrally distinct, occupying different sub-bands within the broader beta range. It is therefore plausible that previously reported, and at times seemingly contradictory, functional roles attributed to beta activity may in fact reflect spectral segregation into low- and high-rhythmicity sub-bands within the band referred to as beta."

We would also like to note to the reviewer, that we already raise this possibility in the introduction citing another work on this line, titled "Low and high beta rhythms have

different motor cortical sources and distinct roles in movement control and spatiotemporal attention", from the same group (line 57-58):

> "On the functional level, at least two of the canonical bands (beta and gamma) are thought to encompass distinct — and even opposing — roles."

Finally, regarding the reviewer's insightful question about whether a single band can be transient in one context and sustained in another: based on the evidence presented in our manuscript, we would argue that this is unlikely. Rather, we propose that each band maintains a relatively stable rhythmicity profile, and that transitions between sustained and transient activity reflect shifts between distinct bands. Apparent within-band changes may arise due to the imprecise frequency boundaries of canonical bands—particularly in the beta range. We now clarify this point in both the Discussion (paragraph cited above) and Results sections. For example, in the Results (line 197-199), we state:

> "the frequency ranges of bands identified by ABBA remained stable across both states (t_{295} = 1.4, *p* = 0.16, BF_{10} = 0.17), underscoring the robustness of the spectral architecture."

Reviewer's comment 1.11:

It is clear that the choice of lag does not influence the relative results - and that the specific lag used is optimal for not flooring or ceiling the results. The method is working well for finding significantly bursty and significantly oscillatory events linked to specific frequencies. However, if two 'oscillatory' or 'bursty' frequencies are too close in peak frequency, the ABBA method will collapse this into one same band, e.g. Fig1-D inside the beta2 band; in this case the second peak is clearly stronger, but if both were comparable in amplitude, only the maximum would be chosen, disregarding the possibility of having two different frequency bands in a small frequency range of same sustained or transient nature?

Response: we thank the reviewer for this thoughtful observation, which indeed follows the logic of the previous comment regarding the interpretation of low- and high-beta as potentially distinct sub-bands within the broader beta range. We would like to note, however, that in the Kilavik et al. (2012) study, the differences in peak frequencies between the reported beta components were relatively large—6 Hz and 4 Hz—which would likely be resolved as separate bands by LAVI, given its sensitivity to such distinctions.

We would also like to clarify a possible misunderstanding in the reviewer's phrasing. The method does not detect bursty events per se but rather is influenced by their presence. Specifically, LAVI is computed over the entire session, not on a per-event basis (although stable and similar sub-bands profiles are observed also in shorter

time frames, as evident in baseline WTPL values, Figure 7). As we show in the revised Results subsection “Low- and high-rhythmicity bands show distinct burst dynamics” and in Fig. 3, there is a clear association between rhythmicity levels and burst statistics when analysed across the full session.

Reviewer’s comment 1.12:

I guess that it would be natural that you cite Rosanova et al. 2009, or other more recent studies for the TMS section? This brings us to the point of the largely lacking discussion section. This seems rather awkward given the huge amount of data covered in the study, the novel methods developed, and novel concepts coined. Is this a feature of the specific journal? Otherwise it would seem natural to discuss the potential limits of the methods, or other comparable approaches?

Response: we thank the reviewer for pointing out the relevance of Rosanova et al. (2009) in contextualising our TMS findings. We have now added a reference to this important work in the Results section (line 507-508):

> “consistent with previous studies [48], this response was reflected in increases in the power across a wide range of frequencies (Fig. 8C).”

Notably, Rosanova et al. used power-based analysis to define the “natural frequency” as the frequency with the highest induced power. To facilitate comparison, we have added a similar power analysis to our manuscript, which yields highly comparable results (Fig. 8C). This comparison highlights a key advantage of our rhythmicity-based approach: it provides finer frequency resolution than power alone, enabling more precise detection and functional differentiation between bands.

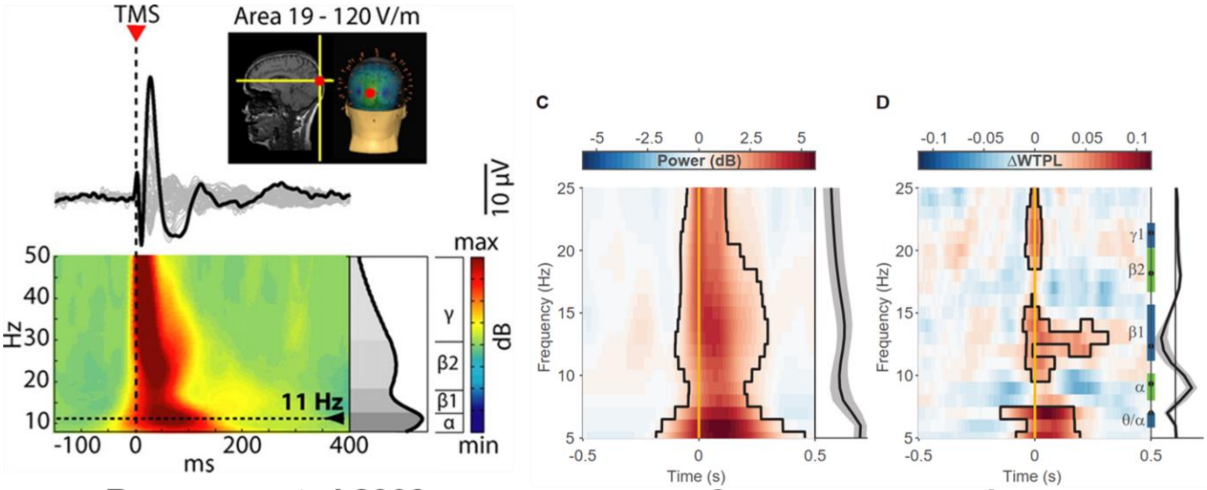

Rosanova et al 2009 Current manuscript

Regarding the broader point about the discussion section, we fully agree that a more extensive discussion is warranted given the scope of the data, the novel methods introduced, and the conceptual contributions made. In response to this comment, we

have substantially expanded the Discussion section. Specifically, we relate directly to the limitations of our TMS study (line 662-668):

> "Although the current protocol yielded informative results consistent with previous reports of spectrally confined responses to single-pulse TMS [48], it was constrained by factors such as sample size and the absence of behavioural correlates. Future studies could strengthen these findings by incorporating larger and more diverse cohorts, as well as concurrent behavioural or task-based measures to directly link rhythmicity modulation with cognitive function. Such refinements would enable a more precise characterisation of how rhythmic and arrhythmic inputs interact with distinct spectral modes and their functional relevance."

Minor

Reviewer's comment 1.13:

We never see raw data, beyond a glimpse of 1 subject in 1C.
Response: We thank the reviewer for pointing out the limited visibility of raw data in the original version. In response, we have added representative plots of raw data in several key figures to improve transparency and interpretability. These are now included in Fig. 1G, Fig. 2B, Fig. 3A, Fig. 8B, and Fig. 8E.

Reviewer's comment 1.14:

Why only a selection of EEG datasets in E and F, instead of all 6 EEG datasets?
Response: following comment 1.3, we changed the way we visualize this figure. Consequently, we focus on one dataset at a time (Fig. 2 and Fig. 3) to allow for clearer interpretation. Subsequently, we present all 6 EEG datasets in Fig. 4A/B/C to provide a comprehensive overview.

Reviewer's comment 1.15:

L96 Not necessarily a novel category of bands, as many recent papers suggest that bursts are very transient (i.e. below your 4 cycles), e.g. Feingold et al. 2015 (2-3 cycles in monkey basal ganglia), or Bonaiuto et al. 2021(<2 cycles in human MEG). Even what you refer to as sustained is in reality transient, as even if it outlasts 6 cycles, it is not truly sustained in time…

Response: we thank the reviewer for highlighting the growing body of work characterising short-lived spectral phenomena, including the important contributions by Feingold et al. (2015) and Bonaiuto et al. (2021). We share the enthusiasm surrounding these developments, which—as noted in the Introduction—were a key

motivation for our investigation into whether a spectral segregation exists between sustained and bursty frequency bands.

While bursts are typically defined as transient events in the existing literature, the frequency bands in which they occur are generally based on canonical definitions (e.g., beta, gamma). In contrast, our findings suggest that sustained and bursty activity are not only temporally distinct but also segregated along the frequency axis. To our knowledge, this spectral distinction has not been previously demonstrated, and it was in this context that we originally used the term ‘novel’.

Nonetheless, to avoid any misunderstanding—particularly the impression that we claim the detection of transient bursts itself is novel—we have removed the term ‘novel’ from the manuscript.

Reviewer’s comment 1.16:

L119 LAVI is only explained in the figure legend (and not here in the results)(and then also in the methods…) I would find it natural to describe it a bit better here in the results, as you aim for ‘online’ methods…

Response: following this comment, we added a description of LAVI to the main text. It reads (line 103-115):

> “We term the algorithm used to resolve rhythmicity across different frequencies at a specific lag the *lagged-angle vector index* (LAVI). LAVI is defined as the vector mean, computed across all time points, of the phase difference between the signal and a lagged copy of itself. As a measure of phase-consistency, it is influenced by the rate of phase-shift occurrence (fig. S1). Importantly, LAVI values show a clear baseline: the median across frequencies is stable across participants, channels, and datasets, and depends only on experimenter-controlled parameters such as lag duration (Fig. 1D) and the window size used for spectral estimation (Fig. 1E). To test whether the LAVI spectrum reflects background noise, we generated surrogate datasets. These were constructed by randomizing phases while matching each dataset’s 1/f power profile—the inverse relationship between power and frequency characteristic of neurophysiological signals (see Methods; Fig. 1F–H). Recorded and surrogate data had similar median rhythmicity values, but the surrogates showed a narrower, flatter range. Leveraging this property, we define the median across frequencies as baseline rhythmicity and the surrogate range as the ‘noise ribbon’ (see Methods and Supplementary Text; fig. S6).”

Reviewer's comment 1.17:

L148 ABBA was not explained in enough detail to understand the results/figure legend… While readers can go to online methods to get details for an analysis or to replicate it, they should get enough information from the main manuscript to grasp intuitively how it works. In particular, this also renders Fig. 1E/F difficult to interpret.

Response: we have changed the layout of Figure 1E/F (now appears in Figure 2). We also added the description of ABBA to the main text (line 141-152):

> "we developed an *automated band-border detection algorithm* (ABBA), which delineates the boundaries of significantly high- and low-rhythmicity bands at the individual subject level. Applying ABBA revealed a considerable measure of consistency across subjects, suggesting a universal rhythmic architecture, while maintaining sensitivity to inter-individual differences within this universality. Supporting universality, peak frequencies of high-rhythmicity bands defined by ABBA across participants were consistently confined to the canonical, power-based frequency bands (Fig. 2B–C) [3]. In particular, we consistently observed a dominant peak within the 6-14 Hz range, aligned with the traditional 'alpha' band, allowing for automatic annotation of band labels. We label this peak as 'alpha' (α); the subsequent trough is labelled 'beta1' (β1), followed by the next peak as 'beta2' (β2), and the following trough as 'gamma1' (γ1). Similarly, the trough preceding alpha is labelled as 'theta/alpha' (θ/α), the adjacent lower-frequency peak as theta (θ), the next lower trough as 'delta/theta' (δ/θ) and the lowest peak considered in this manuscript is labelled delta (δ) (see Fig. 2A)."

Reviewer's comment 1.18:

L183-187 You here use oscillations to also talk about transient bursts. Maybe there should be a clear set of definitions somewhere, as to me an oscillation reflects both 'sinusoidal' and sustained…

Response: In line 351, "characterized with transient oscillations" was changed into "dominated by brief bursts".

Reviewer's comment 1.19:

Fig. 1 C/D if these are the same subject, should the 10Hz label in C rather be 12Hz, since in D 10Hz is neither rhythmic nor transient?

Response: we thank the reviewer for this careful observation. The panels indeed show data from the same subject. The apparent discrepancy arises because panel C presents a short 600 ms snippet used purely to illustrate the method, whereas panel D shows the LAVI computed over the entire session. The subject's alpha peak is at 11.2 Hz.

To maintain consistency between panels C and D, and in line with the reviewer's suggestion, we have updated the snippet in panel C to reflect an 11 Hz oscillation. We appreciate the reviewer's input, which helped us improve the clarity and coherence of the illustration (LAVI at 11 Hz = 0.69, compared to 0.63 at 10 Hz).

Reviewer's comment 1.20:

When calculating the WTPF across subjects, it would be nice to see how the peaks of frequencies are distributed in the subjects shown in Fig2-F. Are all of the subjects the ones from Fig1-G?

Response: we thank the reviewer for this constructive suggestion. In response, we added the distribution of peak frequencies in the new Fig. 8D. To clarify, the subjects in the TMS study (i.e. those for whom WTPL was computed) are distinct from those in Fig. 1 and Fig. 2.

Reviewer's comment 1.21:

When calculating the HF response and covariance with LF amplitude, what happens if the channel response is not systematic across frequency ranges or analysis windows? If HF is significantly increasing in one of the windows and significantly decreasing in another, what is considered for that channel? Is it the biggest modulation for deciding whether it is positively or negatively responding? Is it rejected?

Response: We thank the reviewer for raising this important point, which may indeed be a source of confusion for readers. The HF response label (increase or decrease) is determined relative to the onset of the visual stimulus, analogous to peri-stimulus firing rate analyses in invasive electrophysiology. This labelling is performed prior to the covariance analysis with LF amplitude. To clarify how channels are classified as showing increased or decreased HF responses, we have added Fig. 7A, which illustrates this distinction more explicitly.

Reviewer's comment 1.22:

Results P3-L115/116. The sentence "we found that the relative rhythmicity between frequencies of individual subjects remains similar over lags" it's complicated to understand. Rephrase for something like "the choice of lag does not influence the relative rhythmicity values obtained across the different frequencies for each individual subject".

Response: we changed this sentence as the reviewer suggested (line 100-101).

Reviewer’s comment 1.23:

Fig 2. Center in 0 the colormap of the WTPL

Response: done.

Typos:

Reviewer’s comment 1.24:

P11-L428: “rhythmicity may index may reflect/explain variability between individuals ..”

Response: following reorganisation of the manuscript, this sentence, including the word ‘index’, was removed.

Reviewer’s comment 1.25:

P8-L261: Fig 2C before Fig 2B
Corrected, thank you.

Sincerely, Bjørg Kilavik and Laura Lopez-Galdo

Reviewer #2 (Remarks to the Author):

I co-reviewed this manuscript with one of the reviewers who provided the listed reports. This is part of the Nature Communications initiative to facilitate training in peer review and to provide appropriate recognition for Early Career Researchers who co-review manuscripts.

Reviewer #3 (Remarks to the Author)

General Comments

This is an ambitious, interesting, and well executed study with deep theoretical implications. It also introduces a novel phase-consistency measure (LAVI) that has significant advantages over lagged coherence. The text and some figures require a substantial re-organization for greater clarity; the current organization results in a chaotic exposition. I recommend that the ms be resubmitted after revisions.

Reviewer’s comment 3.01:

The key conclusions stated in the Abstract are:

1. "we found a universal spectral architecture divisible into two categories: high-rhythmicity bands associated with sustained oscillations and novel low-rhythmicity bands dominated by brief oscillatory bursts"
2. "sustained bands signify maintenance of ongoing activity, whereas transient bands signify the brain’s response to change"

The first conclusion is well supported by the data. The second conclusion makes a great deal of theoretical sense, but seems less well supported by the data. Perhaps if the manuscript were re-organized to make a clear separation between theoretical considerations and interpretation of data, the empirical support for conclusion #2 would become clearer. In its current form, conclusion #2 appears to be based entirely on data that are not shown.

Response: we sincerely thank the Reviewer for their thorough, thoughtful, and constructive comments. The feedback has been invaluable in helping us clarify key aspects of the manuscript and improve its overall quality. We believe the revisions made in response to each comment have strengthened the manuscript considerably.

Specifically for this comment, we carefully followed the recommendation and agree that it has helped to improve the clarity and structure of the manuscript. In particular, we have re-organised the manuscript to more clearly distinguish theoretical considerations from empirical findings. We now dedicate a separate section—“Dual Rhythmic Modes and Input Processing” (starting at line 421)—to provide direct empirical support for conclusion #2. We hope this revised structure makes the basis for our interpretation more transparent.

We also rephrased the sentence in the abstract, to reflect it being a theoretical considerations and interpretation of data (line 29-31):

> “This architecture reflects two modes of operation: sustained bands suitable for maintaining ongoing activity, and transient bands which can signal responses to change.”

Reviewer’s comment 3.02:
There are also "four testable predictions" that are listed in the Introduction:
"(i) There should be defined frequency “sustained bands” that clearly express sustained oscillations.
(ii) Additionally, there should be a separate, novel category of bands specializing in transient activity. In these “transient bands”, spectral phenomena should manifest as short-lived bursts.
(iii) The architecture is a universal organizing principle of the brain’s operating system;

hence it should arise across datasets, recording techniques, brain areas, and species.
(iv) The architecture mediates cognition, hence its operation should be sensitive to variations in cognition arising with age and neuropathology, and to different cognitive states, such as input processing."
Predictions (i), (ii) and (iii) are confirmed by the presented data.

Response: We thank the reviewer for this helpful observation. In response to this comment, as well as comments 3.3 and 3.22 below, we have revised prediction (iv) in the Introduction to read (line 77-78):

> "If the architecture mediates cognition, its distinct modes of operation should differentially respond to varying cognitive states and input processing."

We believe that the expanded analysis and new data presented in Figs. 2, 7, and 8 provide empirical support for this prediction. We hope the revised framing and additional evidence will clarify how this aspect of the theoretical framework is substantiated by the data.

Reviewer's comment 3.03:

Frequent reference is made to "our universal rhythmic architecture hypothesis", but that hypothesis is never clearly articulated. It would help to describe the hypothesis in full detail in the Introduction.

Response: We thank the reviewer for highlighting the need for a clearer and more comprehensive articulation of the hypothesis. In response, we have now explicitly defined the hypothesis in the Introduction (line 64-70) as follows:

> "We therefore hypothesized that neural activity is organized within a rhythmicity-resolved spectral architecture. This architecture is defined not only by frequency but also by a rhythmicity axis that distinguishes between sustained (high-rhythmicity) and bursty (low-rhythmicity) modes of activity. This spectral segregation carries functional significance: it allocates specialized frequency bands to support two distinct processes—the maintenance of ongoing activity, which we hypothesize to be reflected by sustained oscillations in high-rhythmicity bands 5,15, and transient responses to inputs, manifested as oscillatory bursts in low-rhythmicity bands."

We hope this clearer formulation helps to convey the conceptual framework underpinning our work.

Reviewer’s comment 3.04:

The TMS experiments in general seem less convincing than the natural frequency band analyses.

Response: We thank the reviewer for this valuable observation. We agree that neurostimulation studies, including TMS, often present interpretative challenges due to their inherent complexity and variability. Nonetheless, we believe the TMS results meaningfully complement the natural frequency band analyses by providing causal evidence for how rhythmic and arrhythmic inputs interact with the proposed spectral architecture.

To address the reviewer’s concern, we have revised the relevant section of the Results (within the newly added “Dual Rhythmic Modes and Input Processing” sub-section, starting at line 421) to clarify the rationale, methodological controls, and interpretation of the TMS findings. In particular, we emphasise how the observed band-specific changes in rhythmicity following rhythmic versus arrhythmic stimulation align with the functional distinctions proposed by our framework. While we acknowledge that these findings are more exploratory in nature, they provide a valuable proof-of-concept and motivate further targeted investigation. To support this, we now dedicate a standalone figure to the TMS results and include additional analyses to contextualise their significance—specifically, a comparison of rhythmicity and power responses (Fig. 8C), and, addressing comments 1.12 and 3.19, visualisation of responses at the raw time-series level (Figs. 8B and 8E).

Specific Comments

Reviewer’s comment 3.05:

Lines 87-88: "Indeed, we recently showed that momentary neuronal oscillations (bursts) occur together with transient firing-rate changes". The word "indeed" implies that the result cited supports Roelfsema's contention that binding is accomplished by rate-coding, but it does not. It is to be expected that oscillatory activity and rate increases go together for simple oscillatory circuits like PING/ING (see e.g. Whittington et al. 2011, Developmental Neurobiology 71, 92–106) regardless of whether either one of them has anything to do with binding.

Response: We thank the reviewer for this important observation. We have rephrased this section, omitting the word “indeed”.

Reviewer’s comment 3.06:

Lines 91-92: "sustained oscillations support phase-coding and maintenance of ongoing activity, whereas bursts reflect rate-coding and response to change" The conflation of the burst-versus-sustained distinction with the rate-coding-versus-phase-coding distinction goes well beyond the scope of the present paper, and is theoretically troublesome as well. Phase-coding inherently conveys information much more rapidly than rate-coding, and is therefore better suited to transmission by bursts, whereas rate-coding requires a sustained response whose duration is roughly proportional to the degree of precision required by the next layer of neural processing.

Response: We thank the reviewer for this thoughtful and theoretically grounded comment. In response, we have moved the discussion of rate- versus phase-coding to the *Discussion* section, where it is now framed more cautiously and explicitly as a theoretical perspective (starting at line 573). We have also moderated the language throughout the manuscript to avoid conflating the burst-versus-sustained distinction with the rate- versus phase-coding framework, in recognition that this relationship extends beyond the scope of the present data.

Reviewer’s comment 3.07:

114-16: "we found that the relative rhythmicity between frequencies of individual subjects remains similar over lags." This sentence is hard to understand. After having read the whole paper, I can retrospectively infer that the authors are saying that the authors get more or less the same sustained oscillation bands and burst oscillation bands from the same subject regardless of the lag used to perform the analysis. However, none of those concepts is explained until later in the paper. Also the use of the term "rhythmic" to mean "sustainedly oscillating" further muddies the presentation. Oscillatory bursts can be just as rhythmic as sustained oscillations, the rhythm just lasts for a shorter time.

We thank the reviewer for this helpful comment. To improve clarity, we have rephrased the sentence in question (line 100-102) as follows:

> “We found that the choice of lag does not influence the relative rhythmicity values obtained across the different frequencies for each individual subject (Fig. 1C).”

This revised phrasing aims to more clearly convey that the identification of sustained and burst-prone frequency bands remains consistent within individuals, regardless of the lag used in the analysis.

To further support this point, we have added a figure (now Fig. 1C) visualising the stability of rhythmicity profiles across lags. This figure was previously included in the

Supplementary Information but has now (addressing comment 3.9) been moved to the main text to improve accessibility and clarity.

Additionally, we now provide an explicit definition of “rhythmicity” earlier in the manuscript (line 95-96), drawing on prior literature:

> “Previous authors defined rhythmicity as “the consistency of the phase relations between timepoints that are separated by some interval (lag) [18].”

We also acknowledge the reviewer’s point regarding the potential ambiguity of using the term “rhythmic” to refer specifically to sustained oscillations. While we retain this terminology for consistency with our framework, we have taken care to clarify our usage throughout the manuscript and to distinguish between rhythmicity as a measurable property and the duration of oscillatory events. We note that this concern is also shared in comment 3.11, and we have addressed it accordingly in both instances.

<u>Reviewer’s comment 3.08:</u>

116-118: "Hence, with carefully chosen parameters, the rhythmicity spectrum can be derived from a single lag (for further discussion, see Supplementary Text, parametrical effects on rhythmicity, and fig. S6)." Given that the main point of Figs. S5A and S5B is that one does not need to be particularly careful in choosing the delay value, it seems odd to say "with carefully chosen parameters".

Response: Following the reviewer’s comment, we deleted the phrase "with carefully chosen parameters".

<u>Reviewer’s comment 3.09:</u>

I think it might be a mistake to relegate Figs. S5A&B to the supplement rather than featuring them in the main text, because they are so central to the issues being explored. Also, some of the exploration of the properties of lagged coherence and LAVI could be profitably moved into the main text.

Response: we thank the reviewer for the helpful suggestion to include key supplementary material in the main text. In response, we now present the properties of lagged coherence and LAVI in Fig. 1 (panels B–H), reflecting their central role in the framework developed in this study.

We have also added the following explanatory text to the main manuscript (line 103-115):

> “We term the algorithm used to resolve rhythmicity across different frequencies at a specific lag the *lagged-angle vector index* (LAVI). LAVI is defined as the vector mean, computed across all time points, of the phase difference between the signal and a lagged copy of itself. As a measure of phase-consistency, it is influenced by the rate of phase-shift occurrence (fig. S1). Importantly, LAVI values show a clear baseline: the median across frequencies is stable across participants, channels, and datasets, and depends only on experimenter-controlled parameters such as lag duration (Fig. 1D) and the window size used for spectral estimation (Fig. 1E). To test whether the LAVI spectrum reflects background noise, we generated surrogate datasets. These were constructed by randomizing phases while matching each dataset’s 1/f power profile—the inverse relationship between power and frequency characteristic of neurophysiological signals (see Methods; Fig. 1F–H). Recorded and surrogate data had similar median rhythmicity values, but the surrogates showed a narrower, flatter range. Leveraging this property, we define the median across frequencies as baseline rhythmicity and the surrogate range as the ‘noise ribbon’ (see Methods and Supplementary Text; fig. S6).”

Reviewer’s comment 3.10:

It should at least be noted that the LAVI in the "sustained" bands is only two times higher than that in the "bursty" bands. This suggests that "bursty" bands contain bursts about half of the time, i.e. the time between bursts is similar to the durations of the bursts.

Response: ee thank the reviewer for suggesting this interesting interpretation of the LAVI score and its potential relationship to burst occupancy.

LAVI (like lagged coherence) is defined as the vector mean of all phase relations between the signal and a lagged copy of itself. As shown in Fig. S1, it serves as a measure of phase consistency, and is primarily influenced by the frequency of phase shifts. While the phase shifts illustrated in Fig. S1 are artificial (hence their placement in the Supplement), bursts represent a physiological example of such shifts.

The reviewer raises an intriguing question: could LAVI values reflect the proportion of time that bursts are “on”? That is, as suggested, might a LAVI ratio of 2:1 between sustained and bursty bands imply that bursty bands contain bursts approximately half of the time?

To explore this, we defined “burst occupancy” as the total duration of all bursts relative to the full session time (following Karvat et al., 2021, PNAS), and simulated its effect on LAVI (figure shown to the reviewer only).

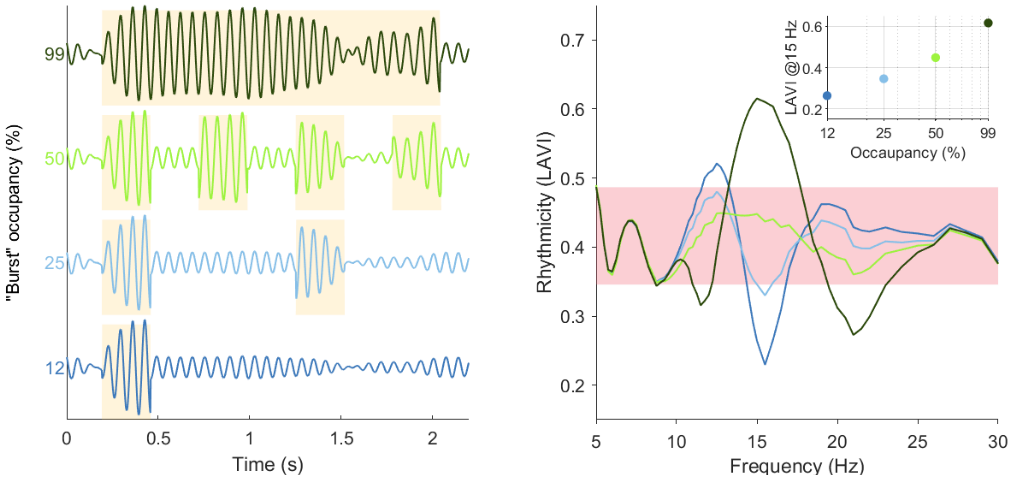

In this simulation (burst duration = 3 cycles; amplitude = 2× 1/f amplitude), we observed that 50% occupancy yielded a LAVI value near 0.5, within the noise ribbon. However, the relationship between occupancy and LAVI was clearly non-linear: for example, 12.5% occupancy yielded a LAVI of ~0.25, while near-total occupancy (~100%) resulted in a LAVI of ~0.6. Doubling occupancy did not double LAVI, and achieving the reviewer’s proposed scenario required highly unrealistic parameters—hence we opted not to include this simulation in the final manuscript.

Empirically, burst occupancy values cluster around 20%, with no significant difference between sustained and transient bands (Fig. 5D; also shown here for convenience).

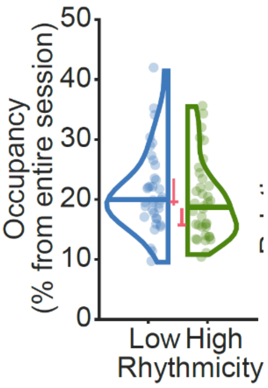

We now reference this analysis in the main text (line 219-220):

> “resulting in comparable total durations during which signals exceeded burst thresholds across bands (Fig. 3E).”

<u>Reviewer’s comment 3.11:</u>

129: "rhythmicity bands" - no, these are "phase-consistency bands", and we need references to Figures 1 and S5 to support the statement. "Rhythmicity" is a more complicated concept that should be discussed in the text, but instead it is used interchangeably with "phase-consistency". As used in this manuscript, "rhythmicity" is introduced as meaning "sustained

vs. bursty" in lines 27-28, which is an excellent first-order definition. Some authors would also insist that "rhythmicity" implies the existence of an underlying rhythm-generator circuit, and there is a simple form of evidence for such circuits that is available in certain types of experiments: if a rhythm can be induced and/or entrained (e.g. by periodic electrical or sensory stimulation), and the rhythm persists for at least a cycle or two after the end of the stimulation, then there must be an underlying rhythm-generator. (Failure to show this does not prove the non-existence of an underlying rhythm-generator, however.) Other expected characteristics of rhythmicity typically include relative constancy of frequency and amplitude. The problematic word here is "relative", because biological oscillators often due drift substantially in frequency and/or amplitude, and this is very likely the reason why a lag of 3 cycles (Fig. S5B) fails to detect phase-consistency bands. In other words, the typical duration of "relative constancy" of frequency in a sustainedly-oscillatory band seems to be about the same as the duration of a typical burst in a bursty band. Ultimately, the gold standard proof of rhythmicity is to elucidate the cellular (or sub-cellular) mechanism of the underlying rhythm-generator.

Response: We thank the reviewer for this thoughtful and detailed comment. We agree that the term *“rhythmicity”* can carry multiple interpretations, and we appreciate the opportunity to clarify our intended usage. In response, we now explicitly state the definition of rhythmicity adopted in this study (line 95-96):

> “Previous authors defined rhythmicity as “the consistency of the phase relations between timepoints that are separated by some interval (lag) [ref 18 = Fransen et al., 2015].”

And further clarify (line 98-99):

> “Here we adopt this definition of rhythmicity, and note that the term is not meant to imply the existence of an underlying rhythm-generating circuit, but rather denotes the degree of phase consistency.”

We have revised the manuscript to ensure that this usage is consistent and clearly distinguished from broader physiological interpretations of rhythmicity. We also acknowledge the reviewer’s broader point that rhythmicity, in a physiological sense, may imply additional properties such as frequency and amplitude stability, or the presence of rhythm-generating mechanisms. While these aspects are beyond the scope of the present study, we agree that they are important considerations and have added a note in the *Discussion* to acknowledge this broader context and its relevance for future work in line 675-678:

> “Might distinct mechanisms underlie the generation of oscillations in the two modes—such as PING (pyramidal-interneuron gamma) circuits for bursty activity [68], versus pacemaker cells for sustained rhythms [69]? (For a comprehensive review, see [70].) “

Reviewer’s comment 3.12:

151-152: The text describes Fig. 1E as "the distribution of peak frequencies of each rhythmicity band across subjects", but there is a disconnect between the bands shown in Fig. 1E&F and those shown in Fig. 1G-J, particularly regarding the beta1 and beta2 bands. The peak of "beta2" spans roughly the 20-28 Hz range in Fig. 1E (roughly the central 50% of the distribution as judged by eye), but about 18-22 Hz in Fig. 1G-J and L ("95% confidence interval per group/ area"). Similarly, the central ~50% of the "beta1" distribution runs 15-20 Hz in Fig. 1E, but the 95% CLs run more like 12-16 Hz in Fig. 1G-J and L. These discrepancies require some explanation. Additional reasons why they are troublesome are described below.

Response: we thank the reviewer for pointing out the inconsistencies in the visualisation of band distributions. In response to this comment—as well as a similar concern raised by Reviewer 1 (comment 1.3)—we have revised the figure presentation for clarity and accuracy. The relevant content is now presented in a dedicated figure (Fig. 2).

Specifically, instead of showing population-level distributions alone (as in the original Fig. 1E/F), we now first display a forest plot of all detected bands for individual participants alongside their raw LAVI rhythmicity profiles (Fig. 2B/C). This is followed by population-level summaries of both LAVI and ABBA metrics (Fig. 2D/E), with means, interquartile ranges, and 95% confidence intervals clearly indicated. This revised presentation allows for a more transparent comparison across figures and better illustrates the consistency of band definitions across individuals and methods.

We have also amended the text to clarify this point (line 145-146):

> “Supporting universality, peak frequencies of high-rhythmicity bands defined by ABBA across participants were consistently confined to the canonical, power-based frequency bands (Fig. 2B–C) [3].

Reviewer’s comment 3.13:

165-166: "transient bands were as common as sustained bands" This paragraph begins with the sentence, "Having confirmed the existence of sustained bands, we next examined whether transient bands exist", but simply showing that some bands have higher phase-consistency than others does not prove that "transient bands" exist. (It should also be noted that it does not prove that "sustained bands" exist either, and the claim should not be made that their existence has been confirmed either.) Therefore, this is much too early to refer to "transient bands" as if their existence has already been proven. It remains to be shown that there actually are transient bursts of oscillation in these bands, and that the lower phase-consistency is due to the extreme brevity of these bursts. (Unfortunately, the actual demonstration of oscillatory bursts, which is well described in Online Methods section "Bursts detection", is

buried in a section that superficially seems to be about phase-versus-rate coding and TMS, "Sub-second manifestations of rhythmicity".)

Response: We thank the reviewer for this important clarification. We agree that referring to “sustained” and “transient” bands at this point in the manuscript was premature, as the presence of transient bursts had not yet been demonstrated. In the revised version, we have addressed this by introducing a new section earlier in the Results, prior to this paragraph, which provides direct empirical evidence that low-rhythmicity bands are dominated by transient bursts, whereas high-rhythmicity bands are characterised by more sustained oscillatory activity (“Low- and high-rhythmicity bands show distinct burst dynamics”, starting at line 200).

This restructuring ensures that the terminology is only introduced after the relevant evidence has been presented. We have also revised the wording throughout the earlier sections to avoid implying that the existence of “transient” or “sustained” bands is assumed in advance of this demonstration.

<u>Reviewer’s comment 3.14:</u>

219-221: "Our hypothesis posits that the universal rhythmic architecture supports both rate- and phase-coding, by allocating “bursty” activity to transient-bands, and “genuine” oscillations to sustained-bands." This is another unfortunate sentence that simultaneously conflates the rate-versus-phase coding issue with the bursty-versus-sustained issue, reveals the excellent depth of theoretical thinking that lurks between the lines, and also implies that brief oscillations are inherently less "genuine" than sustained oscillations. It might be a good idea to defer the deep theoretical discussion to the Discussion section (and if necessary the Supplemental materials) and stick with data-descriptive terminology here. It would certainly be an improvement to avoid implying that rate-versus-phase coding is the same distinction as bursty-versus-sustained oscillation, regardless of where these two issues come up. However, this or one of the earlier passages cited above would be a good place to clarify what is meant by "genuine" versus "false" oscillations. Genuine oscillations come from oscillators, and false ones do not. Jones et al. (2007) Journal of Neuroscience, October 3, 2007 • 27(40):10751–10764 provides a wonderful example of how what appears to be a 1.5-cycle burst of beta oscillation can be explained by a cellular mechanism that superposes two bell-shaped bursts of activity that have different temporal widths but the same peak time to produce exactly the right waveform. If the proposed mechanism is correct, the waveform would have to be called a "false" oscillation because there is nothing oscillatory about the underlying mechanism. The waveform itself, unfortunately, provides no clues as to whether it is a false oscillation produced as proposed, or a true oscillation produced by a beta-frequency oscillator that somehow gets disabled almost immediately after it starts. It is unfortunate that no waveforms of transient versus sustained oscillations are shown in the figures. This would be a great enhancement to the paper.

Response: We appreciate the reviewer's insightful feedback regarding the conflation of rate-versus-phase coding with bursty-versus-sustained oscillatory activity. In response, we have substantially revised the relevant section to avoid theoretical overreach in the Results. Specifically, we have:

- Moved the discussion of rate- versus phase-coding to the *Discussion* section, where we can more appropriately explore the theoretical implications.
- Removed references to "genuine" or "false" oscillations, which we agree could be misleading or imply unjustified assumptions about underlying mechanisms.
- Rewritten the introductory sentence in the Results section to focus on data-driven terminology and avoid conflating distinct conceptual issues. The revised text can be found in the sub-section titled *"Low- and high-rhythmicity bands show distinct burst dynamics"* (starting at line 200).

In addition, following the reviewer's excellent suggestion, we now include **examples of raw bursts** across different frequency bands, as well as the **mean waveform across all bursts**, in **Figure 3A**. This addition helps illustrate the diversity of burst morphologies and supports the distinction between transient and sustained rhythmic activity in a more empirical and visually accessible manner

Reviewer's comment 3.15:

237-240: The ANOVA results are very difficult to unpack from this block of text. They would be enormously much clearer if they were provided in tabular form and simply referenced in the text.

Response: We thank the reviewer for pointing out that the statistical results were difficult to interpret in their original form. To improve clarity, we have simplified the presentation in the main text by collapsing the data across frequency bands according to rhythmicity level (i.e., low- and high-rhythmicity). This allows for a more streamlined and accessible summary of the key findings.

The full set of comparisons, including results for each individual band across all datasets, is now provided in **Supplementary Figure S3**. In addition, we include a **comprehensive summary table** in **Supplementary Table S1** (referred to in the main text, e.g., in line 218), which presents all relevant statistics in a clear and organized format for readers who wish to explore the detailed results.

Reviewer's comment 3.16:

240-242: "Post-hoc Tukey Honest Significant Difference tests revealed higher burst-rates and shorter burst-durations in all transient bands compared to their neighbouring sustained bands." Finally, we have the result that low phase-consistency bands truly are dominated by brief bursts of oscillation! This result also makes good on the claim that higher phase-

consistency bands show fewer but longer periods of oscillation, i.e. "sustained" oscillations. It comes as a surprise, however, how little difference there is in duration between low phase-consistency band oscillations (about 2.2 cycles on average) and high phase-consistency (about 2.6 cycles on average).

We thank the reviewer for highlighting both the significance of the observed differences in burst dynamics and the relatively modest difference in burst durations between low- and high-rhythmicity bands. We agree that the initially reported durations were shorter than might be expected, and we believe this is largely due to the strict criteria we used to define burst duration—specifically, requiring that power at the *peak frequency alone* exceed the 75th power percentile.

In response to this comment (as well as Comment 1.9), we explored alternative definitions of burst duration to better capture the underlying rhythmic activity:

1. **Multi-frequency power-based duration**: We first extended the definition to include all frequencies that exhibited peaks during the burst. This yielded longer average durations—3.5 vs. 2.9 cycles for high- and low-rhythmicity bands, respectively.

2. **Rhythmicity-based duration**: We then applied a time-resolved measure of rhythmicity, the Within-Trial Phase-Lock (WTPL), which estimates local phase consistency. Using the 75th percentile of WTPL as a threshold, we found even more pronounced differences in burst duration—5.3 vs. 3.6 cycles.

Full statistical details for all measures are provided in **Supplementary Table S1**. The results are now reported in the main text (starting at line 200), and illustrated in **Figures 3B and 3C**. We also note in the text that power-based definitions likely underestimate the true duration of rhythmic activity, as seen in **Figure 3A**, where oscillations often persist beyond the power threshold, albeit at reduced amplitude (line 216-218):

> “bursts in high-rhythmicity bands lasted significantly longer (3.5 vs. 2.9 cycles), a pattern amplified when using Within-Trial Phase-Lock (WTPL [22]), a time-resolved measure of phase-consistency (5.3 vs. 3.6 cycles; Fig. 3B-C.”

<u>Reviewer’s comment 3.17:</u>

It is a shame that the introductory paragraph of this section is so full of distractors. This problem could easily be solved by devoting a whole section to the proof of the fact that low phase-consistency bands are bursty bands, and this proof certainly deserves a whole section to itself. The histograms of Fig. 2A are much appreciated, and should be mentioned in the text in place of the ANOVA results. This would be a good place to show waveforms and describe the differences between sustained and transient bands. A plot similar to Figure 2C

but for the physiological data would be very helpful as well. (It is true that Fig. 2C shows only the surrogate data, isn't it? This should be more clearly stated in the figure legend.) The analysis of the surrogate dataset with random phases is a good control analysis, and belongs in the same section, as does the analysis of the simulated data in lines 258-271.

Response: we thank the reviewer for this thoughtful and constructive suggestion. In response, we have reorganized the relevant content to improve clarity and focus. Specifically, we now devote an entire section to the demonstration that low phase-consistency bands are characterized by bursty dynamics. This new section is titled *"Low- and high-rhythmicity bands show distinct burst dynamics"* (beginning at line 200).

To enhance interpretability:

- The **histograms** originally shown in Figure 2A have been updated for clarity. We now collapse across all low- and high-rhythmicity bands and include additional measures to better illustrate the differences. The full analysis across all frequency bands and EEG datasets is now presented in **Supplementary Figure S3**, with complete statistical details provided in **Supplementary Table S1**.

- As suggested, we now include **examples of raw waveforms** and describe how they differ between sustained and transient bands. These additions help to visually and conceptually distinguish the two types of rhythmic activity.

- Regarding **Figure 2C** (now **Figure 3I**), we clarify in the figure legend that this plot is based on simulated data, as it requires systematic manipulation of burst properties that is not feasible with empirical data. The updated legend now reads:
  "I. Rhythmicity profiles of the simulated data."

- Finally, the **analyses of surrogate data with randomized phases** and the **simulated data analysis** (originally described in lines 258–271) are now integrated into the same section, as recommended. This consolidation provides a more coherent narrative around the validation and interpretation of burst dynamics.

<u>Reviewer's comment 3.18:</u>

272-273: "To causally demonstrate periodic input's role in rhythmicity, we stimulated six participants' prefrontal cortices with TMS (Fig. 2D)." This is a completely separate topic and belongs in a separate section, and Figs. 2D-F belong in a separate figure as well. To quote from the Nature Communications submission guidelines, "Depending on the word count,

Articles may have up to 10 display items (figures and/or tables)." There is no need to cram separate topics into a single figure when you only have a total of 4 display items in the manuscript. The rest of this paragraph illustrates particularly well what I think is the chief defect of the whole manuscript, namely that empirical results are combined with thought-provoking theoretical issues and ill-founded claims of making successful predictions (in this case not because they were successful, but because they were trivial). It is as if an excellent, well-structured paper had been put into a blender, and we are reading the result of thinning the homogenate into a monolayer.

Response: We thank the reviewer for pointing out that the TMS experiment introduces a distinct topic that merits its own space. In the revised manuscript, we have reorganized the content accordingly. The experiments and analyses related to the role of rhythmic architecture in input processing now appear in a dedicated section titled *"Dual Rhythmic Modes and Input Processing"* (starting at line 421).

This section includes two distinct experiments:

1. A visual stimulation study using ECoG recordings, now presented in **Figure 7**.
2. A TMS study targeting prefrontal cortex, now presented in **Figure 8**.

Separating these experiments into their own section and figures improves clarity and aligns with *Nature Communications* guidelines on display items. It also allows us to present each dataset and its implications more clearly and cohesively.

In addition, we have reviewed the entire Results section to remove nonessential theoretical commentary, ensuring that empirical findings are presented with appropriate focus and precision.

Finally, we appreciated the reviewer's vivid metaphor. While the original presentation may have resembled a homogenized monolayer, we hope the revised manuscript now reveals the underlying structure and richness of the data—more akin to a well-prepared gel, where distinct bands can be clearly resolved and interpreted.

<u>Reviewer's comment 3.19:</u>

276-277: "we predicted that single impulses should yield transient oscillations ("bursts" or "ripples") as their spectral fingerprint" It has been known for many years that single TMS pulses administered to a healthy, awake brain produce long-lasting and complicated oscillations in the EEG, and indeed the complexity of the oscillations has been successfullly used as an index of patients' states of consciousness when they are non-verbal. Performing a spectrogram or wavelet analysis on a complicated series of peaks and valleys of variable timing will indeed produce a time-frequency plot that appears to have many brief bursts of oscillation at many different frequencies, so the predictions mentioned in this paragraph are

not very impressive. However, there is no reason to expect that any of the apparent oscillations will turn out to be "genuine" oscillations, nor that they will be limited to the "bursty bands" previously identified in this manuscript. How does the authors' hypothesis lead them to expect that they will confined to "bursty bands"? Is this a reference to the above-quoted hypothesis that "bursts reflect rate-coding and response to change"? Is there an implicit assumption that a TMS pulse is in some way equivalent to the onset of a sensory stimulus? Is rate-coding a necessary part of any of those expectations or assumptions? There is a great deal left out here.

Response: We thank the reviewer for raising these important points and for prompting us to clarify the rationale behind our expectations regarding the spectral and rhythmic response to transient stimulation.

In the revised manuscript, we now explicitly state the reasoning that led us to hypothesize that responses to brief inputs would be confined to transient bands. At the beginning of the new section *"Dual Rhythmic Modes and Input Processing"* (line 423-425), we write:

> "We asked whether sustained rhythms, being stable over many cycles, support ongoing maintenance, whereas transient rhythms, being brief and event-linked, reflect responsiveness to change."

This framing reflects our working hypothesis that transient bands are preferentially engaged by discrete, time-locked inputs—whether sensory or externally applied—due to their temporal dynamics and sensitivity to change.

In response to this comment and to Comment 1.12, we have added raw EEG traces following TMS pulses, along with a direct comparison between power and rhythmicity (Figures 8C and 8D), to better contextualize our findings within the broader literature. As now described in the main text (line 505-512):

> "Single TMS pulses evoked characteristic EEG responses consisting of a sequence of peaks and troughs ("ripples"; Fig. 8B). In the frequency domain, filtering transient responses (delta functions) can produce apparent broadband power increases. Indeed, consistent with previous studies [48], this response was reflected in increases in the power across a wide range of frequencies (Fig. 8C). However, according to the rhythmic architecture framework, the rhythmic response to brief events should be confined to the transient bands. To test this, we measured time-resolved rhythmicity (WTPL), and found that ripples were associated with significantly increased rhythmicity specifically in the transient bands θ/α, β1, and γ1 (p<0.05, cluster permutation test; Fig. 8D), lasting up to 300 ms post-stimulation."

We agree with the reviewer that a TMS pulse is not equivalent to the onset of a sensory stimulus. For this reason, we employed two complementary approaches to examine the relationship between rhythmic architecture and input processing: one

using visual stimuli (as a naturalistic input), and one using single and repetitive TMS (as a controlled, externally applied input). This dual approach allows us to test the generalizability of the transient/sustained distinction across different input modalities.

Finally, we have moved the broader discussion of rate-coding to the *Discussion* section, where it can be more appropriately contextualized.

Reviewer's comment 3.20:

279-281: "Our TMS approach indeed provided causal evidence that in low-rhythmicity bands transient activity is reverberated, whereas sustained bands are dominated by ongoing oscillations." This is another very densely-packed, though-provoking sentence that urgently needs to be unpacked! First off, is it saying that the TMS experiments actually did confirm all the expectations and justify all the assumptions (whatever they were)? Second, is it saying that the authors' theory is that bursts of oscillation represent reverberatory activity (i.e. a delay-line-based oscillator) whereas sustained oscillations represent local microcircuit network activity (of which PING/ING would be a simple example)? And finally, does the authors' theory postulate that local network oscillations are necessarily sustained and cannot be responsible for brief bursts of oscillation? If so, why?

Response: we thank the reviewer for pointing out that this sentence was overly dense and potentially ambiguous. In the revised manuscript, we have removed this sentence to avoid conflating empirical findings with broader theoretical interpretations. Instead, we now present the results of the TMS experiment in a more focused and descriptive manner, without implying confirmation of all theoretical assumptions.

Reviewer's comment 3.21:

353-354: "Consistent with our hypothesis, single-pulse stimulations elicited reverberations in frequencies corresponding to the transient bands θ/α and beta1" How do the authors know that they are reverberations, and not successive stops in a long open-loop delay line (analogous to the brainstem auditory evoked potential)? The rest of this paragraph would be considerably more convincing if plots were shown of time-domain waveforms in addition to the plots of Figures 2F&G. Ideally, these should be a number of single-trial waveforms superimposed, each aligned at the onset of the stimulation. This would show not only the time course of the development (or lack) of oscillations, but also the degree to which the oscillations are temporally aligned to the individual pulses in the stimulation, and the degree to which the stimulation actually interfered with previously ongoing oscillations. The concluding sentence, "These results thus support our hypothesis..." would then be well-justified.

Response: we thank the reviewer for this thoughtful comment and for encouraging a more precise and transparent presentation of our findings.

In the revised manuscript, we have removed the figurative use of the term *"reverberations"* and replaced it with a more neutral and descriptive phrasing. Specifically, we now state that the TMS-evoked responses were *"confined to the transient bands"* (line 509 and figure 8D), which more accurately reflects the empirical observation without implying a specific underlying mechanism.

To address the reviewer's excellent suggestion, we have added **time-domain plots** of the EEG responses, including both **single-trial waveforms** and **trial-averaged traces**, aligned to the onset of stimulation. These are now shown in **Figures 8B and 8E**. We agree that these additions significantly strengthen the interpretation of our findings. They illustrate:

- The time course of oscillatory activity following single-pulse stimulation,
- The temporal alignment of oscillations to individual pulses in rhythmic stimulation,
- And the disruption of ongoing oscillations—particularly in the alpha band—by arrhythmic stimulation.

These additions provide a more comprehensive view of the data and, we believe, offer stronger support for the conclusions drawn in this section.

<u>Reviewer's comment 3.22:</u>

370-371: "two types of processes: change in response to inputs, manifested in oscillatory bursts which are transient, and maintenance of ongoing activity, reflected in sustained oscillations" This distinction makes excellent sense conceptually, and should be mentioned much earlier in the paper as part of a detailed description of the hypothesis under test.

Response: we thank the reviewer for highlighting the conceptual clarity of this distinction and for suggesting that it be introduced earlier in the manuscript. In response, we have revised the introduction to more clearly articulate the hypothesis under investigation. The updated text now reads (line 67-70):

> "This spectral segregation carries functional significance: it allocates specialized frequency bands to support two distinct processes—the maintenance of ongoing activity, which we hypothesize to be reflected by sustained oscillations in high-rhythmicity bands [5,15], and transient responses to inputs, manifested as oscillatory bursts in low-rhythmicity bands [16,17]."

We believe this earlier and more explicit statement of the hypothesis improves the conceptual framing of the study and helps guide the reader through the subsequent analyses

Reviewer’s comment 3.23:

372-373: "Our fourth prediction therefore suggests that patterns of activity within the rhythmicity architecture should respond to cognitive states, and particularly those related to inputs." The use of the word "therefore" seems to imply that there is some deductive logic to be followed here, but that is misleading and makes the sentence harder to understand. I believe the intended meaning would be better stated as, "Our fourth prediction is that patterns of activity within the rhythmicity architecture should respond to cognitive states, and particularly those related to inputs."

Response: we thank the reviewer for pointing out the misleading use of the word *“therefore”*, which was not warranted in this context. We have revised the sentence for clarity and relocated it to an earlier part of the manuscript. It now reads (line 187-188):

> “This alignment also enabled us to explore the functional relevance of the spectral architecture and to test our prediction that rhythmicity patterns are modulated by cognitive states.”

We believe this revision improves both the clarity and logical flow of the manuscript.

Reviewer’s comment 3.24:

377-379: "If activity in sustained bands entails maintenance of ongoing activity as opposed to response to input, then rhythmicity levels in these bands should decrease in the presence of sensory input." The validity of this statement is far from self-evident, and it takes some imagination to guess the assumptions that make it sensible. If we assume that there is some sort of trade-off between activity in sustained bands and activity in transient bands, then it makes sense. If we assume that changes in sensory input give rise to transient responses including transient oscillatory bursts, and we assume that transient bursts cannot be produced in sustained bands, then a rapidly changing sensory environment should produce more activity specifically in the transient bands (but possibly without changing the phase-consistency in those bands). However, if we assume that transient bursts can be induced in all bands, then we should see the phase-consistency go down in sustained bands, whereas the phase-consistency might already be close to its floor value in the transient bands. All of these assumptions and their logical relations to each other should be articulated early in the paper, before the presentation of Results.

Response: We thank the reviewer for highlighting the need to clarify the assumptions and logic underlying this prediction. In response, we revised the *Introduction* to more explicitly articulate the conceptual framework guiding our study. The revised text now reads (line 64-70):

> "We therefore hypothesized that neural activity is organized within a rhythmicity-resolved spectral architecture. This architecture is defined not only by frequency but also by a rhythmicity axis that distinguishes between sustained (high-rhythmicity) and bursty (low-rhythmicity) modes of activity. This spectral segregation carries functional significance: it allocates specialized frequency bands to support two distinct processes—the maintenance of ongoing activity, reflected by sustained oscillations in high-rhythmicity bands [5,15], and transient responses to inputs, manifested as oscillatory bursts in low-rhythmicity bands. [16,17]"

Additionally, at the beginning of the new section *"Dual Rhythmic Modes and Input Processing"*, we revised the original sentence to avoid implying a deductive inference and instead clearly state the empirical question being addressed. The updated sentence now reads (line 422-425):

> "To investigate how this architecture supports cognition, we next examined how its distinct modes operate on shorter timescales during input processing. Specifically, we asked whether sustained rhythms, being stable over many cycles, support ongoing maintenance, while transient rhythms, being brief and event-linked, reflect responsiveness to change."

We believe these revisions make the underlying assumptions and logic more transparent and provide a clearer foundation for interpreting the results that follow

Reviewer's comment 3.25:

386-388: "a significant increase in rhythmicity with eyes closed was observed also in the sustained band beta2 but was not observed in the transient band beta1" This is a very interesting observation! But, to be clear, does "eyes closed" here mean "rest with their eyes closed" (mentioned a few sentences earlier)? Is it possible that alpha indicates visual "idling" (a common interpretation), whereas beta2 indicates cognitive "idling"? And does this imply that the bursty beta1 band indicates cognitive activity, in particular changes in cognitive activity? These issues should all be discussed in the Discussion, but could be briefly alluded to here as part of the Results presentation.

Response: we thank the reviewer for these thoughtful and insightful interpretations. Indeed, our conceptualisation of the cognitive roles of the different bands aligns closely with the reviewer's suggestions. We now elaborate on this point in the revised *Discussion* section, beginning on line 601-615:

> "An important advantage of introducing rhythmicity as an additional dimension is that it allows us to build upon, rather than discard, prior knowledge. We do not suggest that all transient bands, or all sustained bands serve the same function. There remains considerable scope for functional specialisation among specific bands. For example, within sustained rhythms, alpha activity is often associated with the maintenance of a widespread, general rest state [63], whereas beta2 has been linked to keeping more targeted forms of cognitive control [61].
> Turning to transient bands, these may reflect inputs from distinct sources. For instance, evoked responses—often considered the frequency-domain analogue of time-domain event-related potentials—are typically dominant just below 10 Hz [64], suggesting that the theta/alpha transient band may reflect broad, stimulus-driven synchronisation. In contrast, beta bursts have been associated with the spontaneous activation of neural networks, where coincident bursts across regions may indicate functional connectivity [65]. In this context, beta1 may be conceptualised as reflecting internally generated inputs, such as packets of action potential sent from one brain region to its targets [66]. These examples highlight the potential of rhythmicity-based segmentation to support a more nuanced functional characterisation of the electrophysiological spectrum in future studies."

We also clarify the sentence from the *Results* section mentioned by the reviewer in line 196 (and see comment 3.26 below).

Reviewer's comment 3.26:

Incidentally, this whole paragraph combined with Figure 1 show the ambiguity of the Greek letter band designations, which becomes problematic. Figures 1E&F show clearly how much overlap there is between neighboring bands, but beta1 and beta2 are shown in their customary ranges, typically specified as 13-22 Hz and 22-30 Hz. Figures 1G-J&L, however, show beta1 as 13-15 Hz and beta-2 as 18-22 Hz, with gamma1 occupying the range of 22-35 Hz that is labeled as "beta2" in Fig. 1E. This makes it hard to understand which bands are being referred to as "the sustained band beta2" and "the transient band beta1" in the present passage. Perhaps the 13-15 Hz band, which is color-coded blue for "transient", and the 18-22 Hz band which is color-coded green for "sustained", might better be referred to as "lower beta1" and "upper beta1", whereas the 22-35 Hz band marked "gamma1", which is color-coded blue for "transient", would usually be regarded as "beta2". ("Low gamma" usually means something like 30-50 or 40-60 Hz.) Or better yet, can we simply dump the Greek letters and denote bands by frequency? That would at least make it clear what frequency band a given sentence is referring to.

Response: We thank the reviewer for highlighting the ambiguity introduced by the use of Greek letter band designations and for pointing out the inconsistencies across

figures. We agree that clarity in band definitions is essential, especially when interpreting results across multiple datasets and visualizations.

In response to this and related comments (1.1, 1.3, 1.17, and 3.12), we have revised **Figure 1** to improve consistency and clarity. Additionally, we now present the **eyes open vs. closed** results in **Figure 2**, where we also introduce the automated band definition algorithm (ABBA) used to define frequency bands in a data-driven manner.

While we retain the use of Greek letter labels to align with longstanding conventions in the field—and to accommodate inter-individual variability—we now explicitly report the frequency ranges associated with each band in all relevant results. For example, in line 195-197 we now write:

> “A significant increase in rhythmicity during rest with eyes-closed was also observed in the beta2 band (18.8-22.4 Hz), but not in beta1 (14.7-17.8 Hz).”

We hope this dual approach—preserving conventional labels while specifying precise frequency ranges—strikes a balance between interpretability, reproducibility, and field-wide familiarity”

<u>Reviewer’s comment 3.27:</u>

402-405: "We found that over the whole session, where in general the amount of input is low, HF significantly covaried with activity in sustained bands. In contrast, in response to a visual input, responding channels transiently increased their HF-LF covariance at a transient frequency band." This is another interesting and relevant statement, but where are the data? There should be a figure showing data that illustrate the statement.

Response: we thank the reviewer for pointing out the need to more clearly present the data underlying this finding. In the revised manuscript, we now describe this experiment in greater detail to improve clarity and interpretability. Specifically, we elaborate on the observed covariation between activity in high-frequency (HF) and low-frequencies (LF). We now reference the figure that presents these data explicitly (line 454-457):

> “We found that over the whole session, where in general the amount of input is low, HF significantly covaried more with activity in the sustained band theta (defined by LAVI and ABBA; Fig. 7C, grey traces; increased-HF channels: 5.0-6.0 Hz; decreased-HF: 5.8-6.4 Hz), and less in alpha (increased-HF: 7.2-10.3 Hz; decreased-HF: 7.8-9.8 Hz).”

This figure now includes the relevant data to support the statement, as requested. We believe this addition strengthens the empirical grounding of the conclusion and

improves the overall clarity of the manuscript.

<u>Reviewer's comment 3.28:</u>

417-422: "In line with these predictions, positively responding channels significantly increased..." Another very intriguing statement that works out very nicely. As always, a bit more connection to the data would be appreciated. Since the title of the original study was, "Distinct Ventral Stream and Prefrontal Cortex Representational Dynamics during Sustained Conscious Visual Perception", I assume there were EoG grids over lateral temporal cortex and lateral prefrontal cortex. Did both sets of grids contain both positively and negatively responding contacts? Was the fraction of each similar in both sets of grids? Were the contacts of each type uniformly distributed throughout the grids, or did they form clusters?

Response: We thank the reviewer for these insightful questions regarding the anatomical distribution of positively and negatively responding contacts. In response, we conducted an additional analysis to examine the spatial distribution of these contacts across recorded brain regions.

This analysis revealed a significant difference in the distribution of increased- and decreased-HF contacts across regions (Chi-square = 39.81, df = 4, p = 4.74e-08; see image below). Based on this finding, and to maintain anatomical specificity, we chose to focus our analysis on the **occipital cortex**, which contained a representative mix of both response types.

Within the occipital cortex, **increased-HF contacts** were broadly distributed, while **decreased-HF contacts** showed a tendency to cluster around the **cuneus** (median MNI coordinates: 4.1, –81.4, 25.5). While this anatomical pattern is beyond the primary scope of the current manuscript, we present it here in response to the reviewer's question.

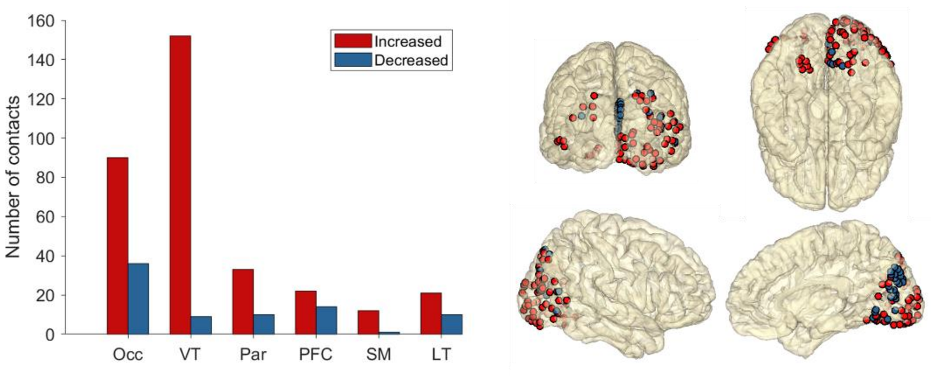

To improve clarity for readers, we now present the HF responses of increased and decreased contacts separately in **Figure 7A**, and we have updated the main text accordingly (line 429-433):

> “As previously reported [29], analysis of high-frequency (HF; 70–150 Hz) signal amplitude—an established proxy for neuronal firing—revealed that among 157 contacts over the occipital cortex, 90 (57%) exhibited increased HF activity in response to a brief (300 ms) visual stimulus, while 36 (23%) showed decreased activity (Fig. 7A; 31 contacts [20%] were non-responsive and are not shown).”

We believe this additional analysis and clearer presentation strengthen the interpretation of the results and directly address the reviewer’s important questions

Reviewer’s comment 3.29:

441-442: "Therefore, we hypothesized that medication would specifically affect the transient-band beta1, and not the sustained band, beta2, of PD patients." The Greek letters rear their ugly heads again! Does "beta2" mean 18-22 Hz here, 20-28 Hz, 22-35 Hz, or something else, e.g. the shaded region in Fig 3D occupying 16-20 Hz (which I would call "upper beta1", not just "beta1")? And what about the 20-28 Hz band, where there is white space clearly visible between the SEM shading, suggesting a p level slightly below 0.05 across the entire band? 20-28 Hz is frequently included in the computation of "beta power" in PD studies.

Response: We thank the reviewer for this important observation regarding the ambiguity of Greek-letter band designations, particularly in the context of (the former) Figure 3D and the medication-related effects in PD patients.

As the reviewer correctly notes, with a significance threshold of α = 0.05, the entire beta range (approximately 15–28 Hz) shows a significant difference between ON and OFF medication states. However, the effect is **most pronounced** in the lower portion of this range—specifically, 15.8–20.0 Hz—where a clear peak in rhythmicity OFF medication becomes a trough ON medication. In the previous version of the manuscript, we focused on this narrower range (referred to as beta1) due to space constraints.

In response to this comment, we now present both the **full significant range** and the **peak effect** more transparently in **Figure 6C**, and we specify the exact frequency ranges in the main text (line 379-384):

> “We found a significant increase in rhythmicity OFF medication compared to ON in the entire beta-band (15.0 - 26.6 Hz, $p<0.05$, cluster permutation test; Fig. 6C). Notably, using a permutation test with stricter threshold of $p<0.01$ the medication effect was localized to the transient band beta1 (15.8 – 20.0 Hz, $p<0.01$). This suggests that while PD pathology elevates rhythmicity across the beta range, the effect is particularly pronounced in the transient band beta1, implicating burst dynamics in the pathophysiology.”

We hope this clarification, along with the updated figure and explicit frequency ranges (line 386-387), resolves the ambiguity and strengthens the interpretation of the findings.

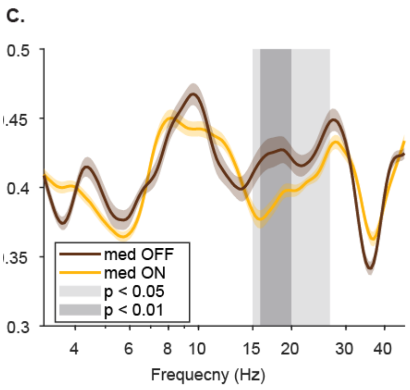

<u>Reviewer's comment 3.30:</u>

486-487: "Rhythmicity-based spectral segmentation reveals twice as many bands as previously described with two divergent modes of operation." Or possibly more; it seems unclear as to whether the typical "beta1" (~12-20 Hz) and "beta2" (~20-30 Hz) bands are each subdivided by this analysis into a transient lower half (12-16 and 20-24 Hz) and a sustained upper half (16-20 and 24-30 Hz), or whether in fact "beta1" (13-22 Hz in Fig. 1E or 13-15 Hz in Fig. 1G) is transient and "beta2" (20-28 Hz in Fig. 1E or 18-22 Hz in Fig 1G) is sustained. This murkiness regarding sustained and transient sub-bands in the 12-30 Hz range needs to be clarified, or at least discussed together with early reports of transient bursts of beta (~18 Hz in rat, Howe et al. 2011 Proc Natl Acad Sci U S A. 2011 Oct 4;108(40):16801-6; 16-20 Hz in monkey, Feingold et al. 2015, Proc Natl Acad Sci U S A. 2015 Nov 3;112(44):13687-92).

Response: we thank the reviewer for highlighting the need to clarify how the bi-dimensionality introduced by our rhythmicity-based method relates to the longstanding ambiguity in band definitions—particularly within the beta range. We agree that this is a critical point, and we now address it explicitly in the revised *Discussion* section (line 616-625):

> "Our consistent observation that broad frequency bands can be subdivided into rhythmically distinct sub-bands may help clarify longstanding ambiguities about their functional roles. For instance, activity within the canonical beta band (~12–30 Hz) has been associated with both sustained and burst-like dynamics. When conceptualised as sustained oscillations, beta is thought to support the maintenance of ongoing motor or cognitive states—for example, continuous beta activity over sensorimotor cortex has been linked to tonic motor inhibition or postural maintenance [60], and sustained beta synchronisation in frontoparietal networks has been associated with stabilising

> cognitive sets and top-down control, suggesting a role in signalling the cognitive status quo [61]. Conversely, when viewed as transient bursts, beta activity has been linked to event-related processes, such as brief motor cortex beta bursts marking movement termination [16] or successful inhibition in stop-signal tasks [62]."

Reviewer's comment 3.31:

487-488: "This indicates that there is room for both rate- and phase-codes with important complementary roles" Same unsupported conflation objected to above.

Response: we appreciate the reviewer's continued attention to the interpretation of our findings in the context of the rate- versus phase-coding debate. While we have retained the sentence in the *Discussion* to reflect one possible implication of the dual-mode spectral architecture, we have substantially revised the manuscript to avoid conflation. In particular, we now present the rate- and phase-code frameworks as complementary perspectives that may be differentially supported by transient and sustained rhythmic modes, without asserting a direct or exclusive mapping. Our intention is not to resolve this complex debate, but rather to highlight how the rhythmicity dimension may offer a useful lens through which to explore it. We hope the revised framing clarifies our position and addresses the reviewer's concern.

<u>Reviewer's comment 3.32:</u>

565: Is LAVI based on a fixed interval independent of frequency, or a fixed phase lag for a given frequency?

Response: We thank the reviewer for raising this important point. We have clarified the method in the revised manuscript with the following sentence (line 752-754):

> "We measured rhythmicity as the consistency of the relations of phase (angle) between data points separated by a fixed number of cycles ("lag", with the corresponding duration in ms determined by each frequency, and see Fig. 1 for considerations for choosing lag duration)."

This clarification makes explicit that LAVI is based on a lag with fixed number of cycles (that is, fixed phase lag), and we hope it resolves any ambiguity.

<u>Reviewer's comment 3.33:</u>

577-578: "The time frequency representation was calculated by multiplying the Fourier transforms of the data and the wavelet of each frequency, and taking the inverse transform of

the product." Clearly this cannot mean multiplying a time-domain signal by a frequency-domain signal, so presumably the wavelet was also Fourier transformed. It is true that multiplying in the freq domain maps onto convolution in the time domain, which is how the wavelet transform is computed in the time domain, but a Fourier transform implies a fixed time window in the time domain and a time-independent spectrum in the frequency domain. The mechanics of how these operations were adapted using moving time windows of various widths is completely unspecified.

Response: We thank the reviewer for pointing out the ambiguity in our original description of the time-frequency analysis. To avoid conceptual conflation and improve clarity, we have revised the sentence as follows (line 764-768):

> “The time-frequency representation was computed by convolving the signal with the Morlet wavelets at each frequency. For computational efficiency, this was implemented in the frequency domain by multiplying the Fourier transform of the signal with the Fourier-transformed wavelets, and applying an inverse Fourier transform to obtain the time-varying complex spectra at each frequency.”

This revised description more accurately reflects the mechanics of the wavelet transform and clarifies that the convolution was implemented in the frequency domain using standard FFT-based methods.

Reviewer’s comment 3.34:

598: the value of n must be specified.

Response: We thank the reviewer for pointing out the need to specify the value of *n* in our surrogate analysis. In response to this comment, as well as Comment 3.43, we have clarified the procedure in the revised manuscript (line 789-791) as follows:

> “In our analysis, we generated n = 200 surrogate noise samples and identified frequencies as sustained or transient if their rhythmicity levels fell within the top or bottom 2.5% (k = 5) of the bootstrap distribution (α = 0.05).”

Reviewer’s comment 3.35:

606-607: "shuffling the data" Why? How?

Response: We thank the reviewer for requesting clarification. We have now elaborated on this point in the revised manuscript (line 798-799) as follows:

> “...initializing with a randomly shuffled version of the original time series to destroy temporal correlations while preserving the amplitude distribution;”

This step ensures that the surrogate data retain the same marginal distribution as the original signal while eliminating any temporal structure, allowing for a meaningful comparison of rhythmicity against a null distribution.

Reviewer’s comment 3.36:

609-611: "assigning the values of the original data to the adjusted time-series based on ranking (the highest value of the surrogate gets the highest value of the original data and so forth)" The confusion intensifies. Point by point? Window by window? Frequency by frequency??

Response: We thank the reviewer for highlighting the need to clarify this step of the surrogate generation process. In the revised manuscript, we now describe the procedure as follows (line 803-805):

> “matching the amplitude distribution: this is done pointwise by ranking the new time series and replacing its values with those of the original series sorted by rank (e.g., the highest value in the new series receives the highest value of the original series, the second-highest receives the second-highest, etc.).”

This operation is performed on the entire time series, not windowed or frequency-specific, and is intended to preserve the amplitude distribution of the original signal while removing its temporal structure.

Additionally, in response to this and related comments, we found it helpful to **rephrase and elaborate the entire IAFFT algorithm** in the revised manuscript to improve clarity and accessibility for readers (highlighted in the revised manuscript).

Reviewer’s comment 3.37:

635: "Spectral parameterization (specparam, formerly fooof" What was the actual frequency band over which specparam was applied? This is important, because the 1/f model is only approximately correct and does not work very well when it is applied over a frequency band that spans more than a factor of 10 or so.

Response: we thank the reviewer for this important observation. Specparam was applied over the 2–40 Hz frequency range. We have now added this information to the manuscript (line 829).

Reviewer’s comment 3.38:

641-643: "we decomposed the signal using an array of Butterworth bandpass filters (order = 3), centred at frequencies 3 to 100 Hz in steps of 1 Hz and bandwidth of 1 Hz," Really??? At 100 Hz, a 1 Hz bandwidth is extremely high-Q and resonant, and a 1 Hz passband implies that temporal resolution is limited to about 0.5 s at all frequencies. Aren't the bursts being simulated of shorter duration than 0.5 s?

Response: We appreciate the reviewer’s concern regarding the filter characteristics and their implications for temporal resolution. Two clarifications are important: (a) the filters were used solely to generate the common signal prior to any manipulation, so any temporal distortions were consistent across all iterations; and (b) the bursts were introduced after filtering, and rhythmicity was assessed using the same method applied throughout the manuscript (wavelet transform with a width of 5 cycles per frequency). We have revised the text to clarify these points (line 832-839):

> “To manipulate the activity in a specific frequency band, we first decomposed the original signal using an array of third-order Butterworth bandpass filters (centred at 3–100 Hz in 1-Hz steps, each with a 1-Hz bandwidth). This decomposition was performed once and served as a common basis for all subsequent manipulations. In each manipulation iteration, we selectively modified the signal in the 15-Hz band and then reconstructed the full signal by summing all band-limited components. Finally, we computed and stored the rhythmicity profile (LAVI) of the reconstructed signal using the same parameters applied throughout the manuscript (wavelet width of 5 cycles and a lag of 1.5 cycles).”

For the reviewer’s reference (not included in the manuscript), we also repeated the simulations using the IAFFT method in place of the filter array and observed comparable results.

Reviewer’s comment 3.39:

646-5647: "we first “notch filtered” the activity in 14-16 Hz by multiplying the filtered signal at this frequency by 0.5." Hard to understand. Are the authors saying that they made FFTs of 0.5 sec long windows so that a single frequency point occupies a 2 Hz band, and multiplied that single amplitude by 0.5? Then the authors would have to transform back to time domain to measure burst frequency. Needs step-by-step explanation.

Response: We thank the reviewer for highlighting the need for a clearer explanation. We have now revised the text to clarify the procedure (lines 840-843):

> “To simulate “bursts” of varying durations in an otherwise quiet band (Fig. 3H-I), we first attenuated the signal filtered at 14–16 Hz by multiplying it by 0.5. We then introduced bursts by multiplying segments of the signal by 2, with a smooth rise and

> fall. Burst duration was the independent variable, ranging logarithmically from 2 to 20 cycles, while the inter-burst interval was jittered between 5 and 15 cycles."

For the reviewer's reference, this manipulation was performed entirely in the time domain, without any FFT-based processing.

Reviewer's comment 3.40:

668: "can be explained as an (artefactual) effect of the peak in alpha" This must have something to do with the comment that creating a simulated peak results in desynchronized sidebands, but it's unclear what.

Response: We thank the reviewer for pointing this out. To clarify the intended meaning, we have added the following sentence at the beginning of the paragraph (lines 863-864):

> "As can be observed in Fig. 3I, fig. S1B, and fig. S5B, increasing (decreasing) the rhythmicity in one band decreases (increases) rhythmicity in neighbouring bands."

Reviewer's comment 3.41:

764-765: "we computed the difference between the phase in the time-point of interest (ϕ 0), one cycle beforehand (ϕ -1) and one cycle after (ϕ 1)." Better description needed! By definition, there is zero phase difference between a time point of interest and the time point one cycle beforehand.

Response: we thank the reviewer for this comment and have further clarified the calculation of within-trial phase locking in the manuscript (lines 973-976) as follows:

> "Like LAVI, WTPL assumes that sustained oscillations are repetitive, and as such, the phase at any time point is expected to predict future and past phases. Specifically, the phase one cycle before or after a time point should be near zero, whereas phase slips cause deviations of this phase relation away from zero."

Reviewer's comment 3.42:

797-798: "To estimate the Low-Frequency (LF) activity, we computed the time-frequency representation of the raw data using complex Morlet wavelets." Given that HF starts at 70 Hz, I presume LF means everything below 70 Hz? Wait, top Morlet freq is 45 Hz... Why use different methods for HF and LF? And what bands are meant by LF and HF?

Response: We thank the reviewer for this thoughtful and perceptive comment, which highlights an important point regarding the definitions and methodological distinctions between LF and HF activity. As we mention in lines 397–398, High-Frequency amplitude (HF, 70–150 Hz) is a well-known proxy for neuronal firing. Some researchers refer to this band as high gamma, but we do not assign it to a specific frequency band in this context; rather, we use it as a proxy for multi-unit activity. The low frequencies (LF, 3–45 Hz) represent the range of the spectrum used throughout the manuscript and include several conventional frequency bands.

We used different methods to define HF and LF in order to closely match the definition of HF from the original publication (Vishne et al., 2023), and to maintain consistency with the time–frequency representation (TFR) methods used for detecting spectral activity in the LF range throughout the manuscript. We clarify this in the text (line 1010-1012):

> “This method was chosen to maintain consistency with the LF analysis used throughout the manuscript, including for LAVI computation. In contrast, the HF estimation followed the approach used in the original publication [29].”

<u>Reviewer’s comment 3.43:</u>

815-817: "Thus, frequencies in the original data with LAVI values higher than the maximal value of the surrogate distribution were defined as having significantly (p=1/20=0.05) high rhythmicity." Are the authors sure this is equivalent to the more usual method of bootstrapping, in which a large number of surrogates are analyzed and the top and bottom 2.5% of values are thrown out to find the p=0.05 levels? I suspect they are probably not equivalent. Do other investigators use the authors’ method?

Response: We appreciate the reviewer’s insightful comment and agree that the original approach—using the maximum value from a relatively small number of surrogate repetitions—does not fully align with standard bootstrapping or Monte Carlo procedures. While this method provided a heuristic estimate of the noise ribbon, it does not yield a statistically robust threshold for significance.

In response, we revised our procedure to follow a more conventional Monte Carlo approach. Specifically, we generated 200 random-phase surrogate datasets to construct a reference table of null distributions, matched to a range of 1/f power profiles and acquisition parameters. For each participant and electrode, we then selected the appropriate thresholds from this table based on their individual power spectra, discarding the top and bottom α/2 values (i.e., 2.5% for α = 0.05 and 0.5% for α = 0.01). This revised method ensures that significance is assessed using a statistically valid framework.

We have updated the main text accordingly (line 295-302):

> "To assess the statistical significance of these bands efficiently, and based on characterization of the parameters affecting the noise ribbon (fig. S6), we first generated 200 random-phase surrogate datasets to construct a reference table of null distributions, matched to a range of 1/f power profiles and acquisition parameters. For each participant and electrode, we then selected the appropriate thresholds from this table based on their individual power spectra, session duration and sampling-frequency. These thresholds were applied within a Monte Carlo framework to determine which observed bands exceeded chance levels. Across the 3–45 Hz range and at $\alpha = 0.05$, the vast majority of detected bands were found to be statistically significant."

And in the Methods section (line 786-791):

> "This relationship can be leveraged to determine spectral bands with statistical confidence using a bootstrap approach: frequencies whose rhythmicity levels exceed (or fall below) the top or bottom k observations from n bootstrap samples of noise can be considered sustained (or transient), corresponding to a two-tailed Type I error rate of $\alpha = k/n$. In our analysis, we generated $n = 200$ surrogate noise samples and identified frequencies as sustained or transient if their rhythmicity levels fell within the top or bottom 2.5% ($k = 5$) of the bootstrap distribution ($\alpha = 0.05$)."

Reviewer's comment 3.44:

828: "Bayes Factor (BF) t-tests" What's the advantage of this lesser-known method over a traditional t-test?

Response: We thank the reviewer for this question and the opportunity to clarify our statistical choices. The goal of the Bayes Factor (BF) analysis was "to test whether or not a difference in band-peak distribution exists." Unlike traditional t-tests, which only allow for rejecting or failing to reject the null hypothesis, BF analysis quantifies the relative evidence for both the null and the alternative hypotheses. This makes it particularly suitable in contexts where the absence of a difference is as informative as its presence. We clarify this in the text (line 1031-1033):

> "To assess whether two or more distributions differed, we computed the likelihood of the data under both the null and alternative hypotheses using Bayes Factors, which quantify the relative evidence for or against a difference."

Reviewer’s comment 3.45:

Supp 47-49: "The wider the Wavelet, the higher the assessed rhythmicity (fig. S5D)." A wide wavelet forces the phase to be strongly correlated over a wide time lag, so a distinction must be made between that analysis artifact and the data-driven correlation determined by the physiology.

Response: we thank the reviewer for this important observation. Following the suggestion, we revised the paragraph to distinguish more clearly between the analytical properties of the wavelet and the relative nature of the rhythmicity measure we employed (Supplementary, lines 44-48):

> “As expected, a wider Wavelet leads to higher assessed rhythmicity (Fig. S5D), since a longer window enforces greater phase consistency by design. This effect reflects an analytic property of the wavelet transform rather than a physiological signal. However, our approach does not rely on absolute LAVI values, but rather on within-subject, within-analysis relative comparisons, such as deviations from the median at each frequency.”

Reviewer’s comment 3.46:

Supp 48: "allows statistical significance inference of each band (see ABBA above). " ABBA is not described "above", it is in the main text.

Response: the reviewer is absolutely right. We have amended the text to read (supplementary, line 75): “see ABBA in the main text, Methods”

Reviewer’s comment 3.47:

Reviewer #3 (Remarks on code availability):

There are some remarks in passing about certain code functions that are available on Github, but there does not appear to be any upload of code or data used to produce the figures.

Response: we thank the reviewer for pointing this out. A source file containing all the data used to produce the figures has now been added to the submission.

REVIEWER COMMENTS

Reviewer #1 (Remarks to the Author):

We appreciate the effort in restructuring the paper for this second submission. Concepts, hypothesis and predictions are clearer; each section responds to one individual question and individual figures focus on testing the specific predictions of the study. We values the single subject data, as well as the population summaries, which also make the claims more interpretable. Separating the phase-coding/rate-coding concepts (moved to discussion) from the sustained/transient nature of oscillations allows for a more direct test of hypothesis, leaving the possible interpretations for the discussion session, which was also considerably extended.

We believe the main claim of the paper, i.e. the idea of including 'rhythmicity' as a relevant feature in spectral analyses provides a new dimension in which we could understand neural activity, and the coexistence of low-rhythmicity with high-rhythmicity bands is a highly systematic result across populations, datasets and recording methods.

The ABBA algorithm helps connecting sustained/transient frequency bands to the canonical frequency bands defined on the frequency dimension. It is useful to detect the significance of high-vs-low rhythmicity by comparing them to the noise ribbon. However, in my opinion, inter-subject frequency variability complicates the generalization of specific frequency band as sustained or transient (e.g. gamma is transient, beta2 sustained, beta1 transient). The data clearly supports the existence of high-and-low rhythmicity bands (both in individual subjects and across populations/recordings), but the dynamics of each frequency band seem to be more subject and dataset dependent.

Finally, the number of bands detected, as well as the similar profile across sexes, the age differences and pathological differences seem clearer as presented now.

Response:

We thank Reviewer #1 for the thoughtful and constructive evaluation of the revised manuscript and for the positive assessment of the restructuring, clarity of the hypotheses, and emphasis on single-subject analyses alongside population-level summaries.

We also appreciate the reviewer's comments regarding inter-subject and inter-dataset variability in frequency expression. This is an important point, and we address it explicitly in our response to comment 1.13.

We thank the reviewer again for the constructive feedback, which has helped us clarify both the scope and the implications of our findings.

Specific comments:

Reviewer's comment 1.01:

From Low- and high-rhythmicity bands show distinct burst dynamics, it is clear that the sustained and transient bands are significantly different. However, it is not very clear why in lines 211-214 there is the idea that sustained corresponds to more than 3 cycles and transient around 1 cycle, when in later analysis results seem to differ (e.g. 3.6 vs. 2.9 cycles) – and it changes based on the method used for duration detection.

Response: we thank the reviewer for pointing out the ambiguity arising from the discussion of burst duration. In lines 211–214, we refer to the number of cycles that are visually observable in the burst-related potential (Fig. 3A), which reflects an average waveform across all bursts within one subject. The later analyses adopt a more quantitative approach by estimating the duration of individual bursts using two complementary metrics: a commonly used power-based measure (Fig. 3B) and a rhythmicity-based measure using the recently introduced WTPL (Fig. 3C). These burst durations are then averaged per subject and compared at the population level.

We agree with the reviewer that referring to "3 cycles" versus "1 cycle" in the former context can be confusing, and we have therefore revised the text accordingly (lines 209–213):

> *Visual inspection at the single-subject level revealed that bursts occurring in high-rhythmicity bands (alpha, beta2) typically showed sustained oscillations sustaining several cycles, with close correspondence between raw and filtered signals. In contrast, bursts within low-rhythmicity bands (theta/alpha, beta1) dampened quickly and displayed phase deviations between raw and filtered traces (Fig. 3A)…*

Also, in simulated data it seems that bursts below 4 cycles correspond to significantly low rhythmicity, which would correspond to sustained bands as well, and longer than 6 to sustained, which does not occur on real data. It is possible that results from simulated data and real data can't be directly comparable, and we agree that the 1/f phase-randomization test is important, but not sure what would be the message we extract from the simulations with generated bursts -if we assume that the cycle numbers can't be directly linked to real data.

Response: We thank the reviewer for raising this important point. The message we extract is that the difference in burst average, computed by the WTPL, can be approximated by simulations. Following the reviewer's comment, we 'zoomed-in' to increase the resolution of the border between noise, low-rhythmicity, and high rhythmicity- instead of looking at burst duration between 2 and 20, we now focus on the range 2-12, and simulated double the number of bursts length (26 compared to 13). This finer analysis revealed that bursts below 3.81 correspond to significantly low rhythmicity, and bursts above 5.45 cycles correspond to sustained activity. These results are similar to what we obtained from the data (3.6 and 5.3 for low- and

high-rhythmicity bands, fig. 3C). We amended the main text accordingly (line 228-231):

> *Bursts shorter than 3.81 cycles yielded rhythmicity below chance levels, while bursts longer than 5.45 cycles exceeded it (Fig. 3H–I and fig. S4), resembling WTPL-based burst durations and supporting a distinction between transient and sustained rhythmicity.*

<u>Reviewer's comment 1.02:</u>
From the LAVI calculation (used in Figure 4A), in the preprocessing EEG methods it is discussed that only a few channels are rejected from the analysis, but then that electrode Cz is used for rhythmicity and burst analysis (line 713). Then, are the values obtained from that unique electrode, or are they the average across the different scalp electrodes? If only calculated based on Cz, did you see any gradient/differences across the different contacts? Should we expect the same results from different locations?

Response: the analysis presented in Fig. 4A is based on one electrode per subject and, as noted in Table 1, was performed using electrode Cz. The use of the same electrode across subjects was chosen to support comparability across different studies involving different tasks and conducted in different laboratories. As stated in line 644-645:

> *Importantly, channel Cz, which was used for rhythmicity and oscillatory bursts analyses, was good for all 178 EEG subjects.*

With respect to the question of whether gradients or differences across scalp locations are observed, this is indeed an important and interesting question that we have been asked repeatedly when presenting this work. Exploring spatial gradients in rhythmicity is beyond the scope of the current paper. Nevertheless, based on previous work (Mahjoory et al., 2020; Capilla et al., 2022; Myrov et al., 2024), we expect that rhythmic architecture may vary systematically across anatomical locations. We have therefore explicitly raised this point as a question for future studies in the Discussion (lines 603-605):

> *Would the characteristics of the rhythmic architecture (e.g. peak frequencies and band ranges) change systematically with anatomical location [19,66,67]?*

<u>Reviewer's comment 1.03:</u>
In Figure 5, we understand the need for the different scales to show the two results, but especially in C, it is very misleading that values of yellow are way higher when in the full graph they are smaller, It could be useful to maybe set the 0.4 (which belongs to both graphs at the same hight (similar to A).

Response: we thank the reviewer for pointing this out and have updated Fig. 5 in accordance with the reviewer's suggestion.

Reviewer's comment 1.04:

Furthermore, in Fig. 5, is the EEG source localized to the same site as the LFP? If not, would one at all expect them to contain the same signals so that a direct comparison is possible?

Response: the reviewer points to an important issue, and we thank them for the opportunity to clarify it here and in the manuscript. In the analysis presented in Fig. 5A, we used sensor-level data, as described in the Methods section:

> *for each area, we included the nearest concurrently recorded EEG scalp electrode.*

We interpret this as strengthening our conclusion of no difference between modalities. That is, even without converting the data to source space, deep (LFP) and scalp (EEG) recordings showed a similar rhythmic architecture. To make this point clearer, we added the following sentence to the main text (lines 321-322):

> *…by comparing invasive signals to those from the nearest concurrently recorded scalp EEG electrode.*

Reviewer's comment 1.05:

From Dual rhythmic modes and input processing, the assumption is that positively responding channels are engaged in the process of the current stimuli and negatively responding channels are engaged in something else, but negatively responding could as well be a response to the stimuli, not necessarily the interruption of something else.

Response (to comments 1.05 through 1.10): we thank the reviewer for the careful consideration of this section. We agree that the interpretation of the rhythmicity changes in Fig. 7 is not sufficiently constrained in the context of the present manuscript, particularly given variability across contacts and time windows.

While we believe that the observations in Fig. 7 are potentially interesting, we agree that addressing the issues raised here would require a more extensive and dedicated analysis. This goes beyond the scope of the present manuscript, whose primary aim is to establish the rhythmicity-resolved spectral architecture across datasets and modalities.

We have therefore decided to remove Fig. 7 and the associated analyses from the manuscript. We believe that this improves clarity and focus, and avoids over-interpreting results that would be better treated in a separate, more detailed study.

Reviewer's comment 1.06:

Figure 7B, when comparing left to right shows another example of the differently defined frequency bands across contacts (comparing the blue/green bands), e.g. beta2-R aligns better with beta1-L and gamma1-R is in between of gamma1-L and beta2-L. The result of high rhythmicity bands showing a negative change in rhythmicity (and lower rhythmicity reverse) is interesting, yet not very clean in the left plot. The beta and gamma bands are not very clearly dissociated between them and it is not very clear to which band is the increased

WTPL associated with, or whether the increase we see corresponds to the gamma band defined by the ABBA.

Response: see response to comment 1.05.

Reviewer's comment 1.07:
Similarly, how different contacts yield different frequency bands, could it be possible that when selecting the whole section vs. the 0-0.2s the bands would also change? It is not clear that it is possible to run the ABBA algorithm on a small subset of data (like the 0-0.2s) but could some differences in Fig7C be associated with that?

Response: see response to comment 1.05.

Reviewer's comment 1.08:
In Figure 7C (left), it seems clear that in the increasing population for the visual input time, there is a clear peak in the transient band, and for both lines there is a decrease in the alpha band. However, it is not very clear if in the whole session the peak is really at theta or just at an in-between of the bands, pointing more towards a reduce in the power than a real change from sustained (gray) to transient (purple).

Response: see response to comment 1.05.

Reviewer's comment 1.09:
Furthermore, 7C (right) is not very convincing as supporting your concept. The increase in ACOV just after visual stimulation is covering both the sustained alpha and the transient beta1, and idem for the sustained theta and the transient gamma1. Thus both transient and sustained bands are modified by stimulus onset, and not in a logic way that would assign roles to transient vs. sustained, but rather as theta/gamma going hand in hand (as often seen in literature) and alpha/beta going hand in hand, as also often seen in literature. In short, given all these issues with this section, we would suggest removing it completely, and instead making a separate, more detailed study of this for another paper.

Response: see response to comment 1.05.

Reviewer's comment 1.10:
Furthermore L459: here you say HF-LF covariance, in the figure legend you say LF-HF covariance or ACOV... please be consistent with naming, the paper is very dense and many abbreviations and concepts that are not used consistently.

Response: see response to comment 1.05.

Reviewer's comment 1.11:
Then for the TMS section, figure 8C is nice as an extension to highlight the importance of the finding in 8D, which seems the most convincing, clear and interesting part of the TMS. 8F is still complex, in particular for the arrhythmic effects. Again, we propose to remove the

rhythmic/arrhythmic part of the TMS, and leave this to a separate study, and potentially only keep the single-pulse TMS result (Fig. 8 B-D)?

Response: we thank the reviewer for this constructive comment and agree that the effects of single-pulse TMS (Fig. 8B–D) are the most immediately intuitive component of the TMS experiment. However, we respectfully believe that the rhythmic versus arrhythmic repetitive stimulation is an essential part of the argument.

First, in the context of oscillatory dynamics, repetitive stimulation is a standard and theoretically motivated tool, as it allows direct interaction with the brain's rhythmic activity. Second, this manipulation provides a critical test of the functional distinction proposed in this manuscript: that sustained (high-rhythmicity) bands support maintenance of ongoing activity, whereas transient (low-rhythmicity) bands reflect responses to discrete inputs.

The differential effects of rhythmic and arrhythmic TMS (Fig. 8F) provide evidence for this dissociation. Rhythmic stimulation enhances phase consistency across bands, whereas arrhythmic stimulation selectively disrupts sustained bands while enhancing transient-band responses. This double dissociation is difficult to explain without invoking distinct functional roles for the two rhythmicity modes.

We agree that this section requires careful explanation, and we have therefore revised the text to clarify the conceptual intuition underlying these effects (lines 441-460) :

> *For repetitive inputs, we predicted that rhythmic stimulation, delivered at a fixed inter-pulse interval (IPI), would enhance EEG-measured rhythmicity. In contrast, arrhythmic stimulation, with temporally irregular IPIs, was expected to violate phase consistency and thereby disrupt sustained oscillatory activity. To test this, we first determined each participant's individual peak frequencies in the alpha, beta1, and beta2 bands. For each band, we delivered 60 rhythmic trains of six pulses, with IPIs matched to the cycle length of the band's peak frequency. The arrhythmic condition consisted of temporally irregular pulse trains matched in overall duration. As with single pulses, each TMS pulse evoked a pronounced transient response ("ripple"; Fig. 8E). During rhythmic stimulation, these responses were aligned with the phase-locked stimulation train, resulting in a significant increase in within-trial phase locking (WTPL) across all bands (Fig. 8F).*
>
> *In contrast, arrhythmic stimulation produced a striking dissociation between rhythmicity modes. In sustained bands (alpha and beta2), arrhythmic stimulation reduced rhythmicity relative to baseline (blue clusters around the peak frequency (0); Fig. 8F), consistent with interference with ongoing oscillatory activity by irregular inputs. Conversely, in the transient band beta1, arrhythmic stimulation increased rhythmicity for up to 200 ms after the final pulse, likely reflecting the accumulation of transient responses, each causing a ripple containing a prominent beta1 component (see Fig. 8D). This double dissociation provides support for our hypothesis: that low-*

> *rhythmicity bands comprise transient yet spectrally defined input-driven activity, whereas high-rhythmicity bands support the maintenance of ongoing oscillations. Thus, the rhythmicity-resolved spectral architecture captures an important distinction in how the brain maintains versus responds to activity.*

In addition, following the reviewer's comments 1.05-1.10, we have removed Figure 7, which we believe reduces cognitive load and allows the TMS results to be more easily appreciated. For these reasons, we believe that retaining the rhythmic and arrhythmic TMS results strengthens the manuscript by providing support for the proposed rhythmicity-resolved architecture.

<u>Reviewer's comment 1.12:</u>
I am not at all an expert on TMS, but I can understand the hypothesis of sustained oscillations enhanced by rhythmic pulses and disrupted by arrhythmic ones. I was just surprised that transient oscillations would also be enhanced (increased rhythmicity) by rhythmic stimulation (Figure 8F-middle). Should this be expected? So, in other words, transient bursts would become more rhythmic by any external stimulation, no matter if it is rhythmic or arrhythmic?

Response: we thank the reviewer for this thoughtful question. Within our framework, low-rhythmicity (transient) bands are not defined by an inability to phase-lock, but by the fact that their activity typically manifests as brief, input-driven responses rather than sustained oscillations. Rhythmic stimulation delivers a sequence of temporally regular inputs, each evoking a transient response ("ripple"), and the repetition of such aligned responses can transiently increase phase consistency, resulting in elevated rhythmicity estimates. This is reflected in our amended text (lines 448-449):

> *During rhythmic stimulation, these responses were aligned with the phase-locked stimulation train, resulting in a significant increase in within-trial phase locking (WTPL).*

Importantly, this mechanism differs from that in sustained bands, where rhythmic stimulation reinforces the network's tendency toward ongoing oscillations, whereas arrhythmic stimulation interferes with this activity. Thus, increased rhythmicity in transient bands reflects the accumulation of stimulus-locked responses rather than the emergence of sustained oscillations.

<u>Reviewer's comment 1.13:</u>
On the discussion, similar comment that before (lines 616-618), I find it difficult to generalize the sustained/transient to specific frequency bands, since we have seen than recording modality, species and different subjects within populations have different frequency bands.

Response: we thank the reviewer for raising this important point and would like to clarify that we fully agree with the premise. Indeed, inter-subject variability, as well as differences across recording modalities and species, complicate any attempt to rigidly assign fixed frequency boundaries or functional roles to specific bands.

Importantly, our framework is not intended to claim that specific absolute frequencies are intrinsically sustained or transient across individuals or datasets. Rather, the central claim is that electrophysiological activity is organized along a rhythmicity dimension, separating sustained and transient modes of activity, while the exact frequency ranges expressing these modes can vary substantially across subjects. This variability is explicitly captured by our individual-level band detection approach (ABBA), as illustrated in Fig. 2D–E and throughout the multi-dataset analyses.

The discussion passage mentioned by the reviewer refers to the subdivision of broad, canonical frequency ranges (e.g. the beta band) into rhythmically distinct sub-bands, which we believe helps reconcile previously conflicting findings regarding their functional roles. This subdivision does not imply fixed frequency assignments, but rather highlights that different rhythmicity modes can coexist within traditionally defined bands.

We therefore view the observed variability not as a limitation, but as a motivation for moving beyond fixed, power-based band definitions toward a rhythmicity-resolved framework that preserves generalizable principles while accommodating individual differences. In line with this perspective, we provide open-source implementations of LAVI and ABBA, with the explicit aim of enabling researchers to identify rhythmicity-resolved bands at the individual-subject level, rather than relying on fixed, group-level frequency definitions

Reviewer's comment 1.14:
In the Figure 3 caption end you say: "Dotted lines mark troughs; arrows indicate phase shifts, likely due to neighbouring high-rhythmicity bands." Would this mean that the high rhythmicity band makes the low rhythmicity band next to it look seemingly low? i.e. is beta1 'obliged' to be low in your analysis, as it is affected by the neighbouring alpha band which has higher power and thus distorts the beta1 signal? This is again taken up in the discussion, where you say that L633-636 : "We observed that high-rhythmicity bands can suppress the emergence of oscillations in adjacent bands via phase interference (Fig. 3A). Specifically, ongoing oscillatory activity in a dominant high-rhythmicity band may curtail the development of nearby rhythms through phase-based interactions (i.e., interference)." Might this only be a problem of your measurement, but not for the neurons engaged in the two neighbouring rhythms? For that one would possibly need intracellular recordings from neurons engaged in the different rhythms, which is of course out of scope for this paper… so maybe best to not speculate too much on this? But it indeed makes one question whether beta1 is somehow dictated a transient label in your analysis, just from happening to be next to alpha?

Response: we thank the reviewer for this careful and important point. We agree that phase interference between neighbouring frequency bands is, at present, a speculative interpretation at the neural level, and we did not intend to imply a direct physiological suppression mechanism between neuronal populations. As the reviewer notes, resolving the cellular or circuit-level mechanisms underlying such

interactions would indeed require invasive or intracellular recordings, which are beyond the scope of the present study.

At the same time, we share the reviewer's concern that the observed low rhythmicity in bands such as beta1 could arise trivially as a measurement artefact due to interference "shadows" from adjacent high-rhythmicity bands (e.g. alpha). We address this explicitly in Supplementary Figure S5, where we show that beta1 troughs are significantly more arrhythmic than expected based on interference effects alone, using both analytically constructed pink-noise surrogates and subject-specific simulations. These analyses demonstrate that the transient classification of beta1 cannot be explained solely by its proximity to a neighbouring high-rhythmicity band.

To avoid any ambiguity, we now explicitly refer to Supplementary Figure S5 in the main text (Results, Fig. 3 section), where interference effects are first discussed, and clarify that the interpretation is restricted to the level of the measured rhythmicity profile rather than implying direct neuronal suppression.

Results section (legend of Figure 3):

> *(see Fig. S5 for control analyses)*

Discussion section (lines 559-562):

> *We observed that high-rhythmicity bands can attenuate rhythmicity in adjacent frequency ranges via phase interference at the level of the measured signal (Fig. 3A; see Fig. S5). Whether such interactions reflect cellular-level mechanisms will require future intracellular recordings and is beyond the scope of the present study. At the level of the measured signal, ongoing oscillatory activity in a dominant high-rhythmicity band may curtail the development of nearby rhythms through phase-based interactions (i.e., interference).*

Reviewer's comment 1.15:

In line 350 you say sustained bands characterized by long-lasting oscillations and transient bands dominated by brief bursts, but even if the difference between 3.6 and 2.9 might be significant, it remains a tiny effect… also with the other measures you test, there will be a great overlap in duration of transient band vs. sustained band bursts, and none of them seem to be 'long-lasting'? Also on line 553 you say sustained and bursty, please be consistent with naming.

Response: we thank the reviewer for raising this point and would like to clarify the quantitative basis of the statement. In Fig. 3B, which classifies bursts based on power-defined bands, the difference in burst duration between conditions is indeed modest (≈3.6 vs. 2.9 cycles). However, when burst-duration is defined based on the rhythmicity measure WTPL (Fig. 3C), the difference becomes more pronounced, with sustained bands exhibiting longer durations than transient bands (mean ≈ 5.3 vs. 3.6

cycles). Together, these analyses indicate that sustained and transient bands differ in burst duration, with the distinction being more clearly expressed when conditioning on rhythmicity rather than on power.

Importantly, our claim is not that sustained and transient bursts form non-overlapping categories in an absolute sense, but that they differ systematically at the level of individual subjects. To directly address the reviewer’s concern regarding overlap, we performed a paired, subject-wise comparison of burst duration between sustained and transient bands (see below). This analysis reveals that, for 36 out of 37 subjects, burst duration in sustained bands is longer than in transient bands.

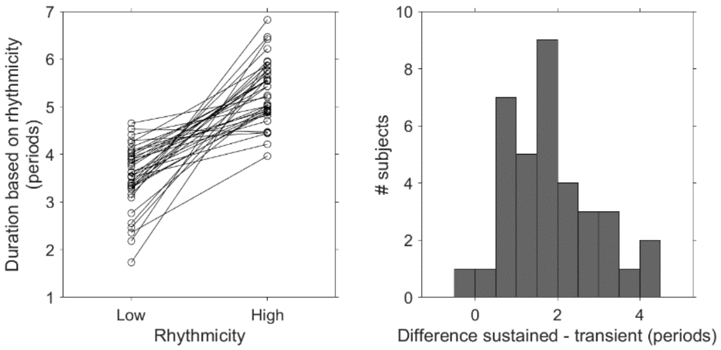

Thus, while individual bursts in both modes are finite in duration, sustained bands are characterized by consistently longer-lived oscillatory events relative to transient bands, supporting the distinction made in the text. We have also corrected the terminology for consistency (line 479).

Sincerely, Laura Lopez-Galdo and Bjørg Kilavik

Reviewer #2 (Remarks to the Author):

I co-reviewed this manuscript with one of the reviewers who provided the listed reports. This is part of the Nature Communications initiative to facilitate training in peer review and to provide appropriate recognition for Early Career Researchers who co-review manuscripts.

Reviewer #3 (Remarks to the Author):

The revised manuscript is head and shoulders above the first draft. The greatly expanded set of figures is an enormous improvement. All of this reviewer's comments have been addressed well, many truly excellently. The goal mentioned in the authors' response to reviewer comment 3.18 of making the paper "akin to a well-prepared gel, where distinct bands can be clearly resolved and interpreted" has truly been achieved. I feel it is ready for publication with minor corrections.

Reviewer's comment 2.01:
There is one recurring typographical issue that should be corrected before the paper goes to press. Typographically, the symbol for "approximately" is "≈" (Unicode 247 = 0xF7), not "±" (Unicode 177 = 0xB1) which means "plus or minus" and can be very misleading.

Response: we thank the reviewer for highlighting this potential source of confusion. The symbol "±" was used in two distinct contexts: first, when reporting error bars (e.g. ± SEM), and second, to denote temporal alignment relative to burst troughs (e.g. ±3 cycles). While the former usage is standard, we agree that the latter can be misleading. Following this comment, as well as comment 1.01 from Reviewer 1, we have removed this notation from lines 211 and 212 and from the legend of Fig. 3. We have also revised the Methods section accordingly (lines 817-818):

> *We then identified the nearest trough in the filtered signal and defined it as time zero (t = 0), extracting an epoch of 3 cycles before to 3 cycles after this point.*

There are also some issues that could optionally be corrected to make the paper more understandable, but they are no reason to hold it back from publication. They are described in the following.

Reviewer's comment 2.02:
Assuming that the phrase "the maintenance of ongoing activity" is meant to include the maintenance of lack of activity as well, a slight modification of the way it is initially spelled out might help. Some possible alternatives would include "the maintenance of current state", "the maintenance of ongoing activity (or lack thereof)", etc.

Response: the reviewer highlights an important nuance, namely that "the maintenance of ongoing activity" may also encompass the maintenance of a lack of activity. Accordingly, and following the reviewer's suggestion, we have amended the text in line 68 to read:

> *the maintenance of current state*

Reviewer's comment 2.03:
The many instances of the abbreviation "HF", defined as "High-Frequency (HF) activity ... as reported in 29", are slightly confusing, especially on a quick read-through. Ref. 29 states: "high-frequency activity (HFA, 70-150 Hz)". This is very clear, and it would help the clarity of the current paper simply to quote it, and then use the abbreviation "HFA" as it is used in ref. 29.

Response: we thank the reviewer for this useful suggestion regarding terminology and clarity. We note, however, that following comments 1.05 through 1.10 from Reviewer 1, the section in which this abbreviation was used has been removed from the manuscript.

Reviewer's comment 2.04:

Legend for Fig. 7A, "Panels B-C are also divided to increased (left) and decreased (right) units": if the recordings are ECoG, they are almost certainly massively multiunit, and should not be referred to as "units". Also, the tail end of the legend for Fig. 7A is not a good place to specify that all of the panels show "increased" channels on the left and "decreased" channels on the right; better to put this information before the panel-by-panel descriptions start.

Response: see response to comment 2.03.

Reviewer's comment 2.05:

Fig. 8B: beta1 filter

There does not seem to be a specification anywhere in the paper of what "filtered in the beta1 band" means. It appears to be filtered with a zero phase shift filter (e.g. Matlab's 'filtfilt' function), and the waveform looks quite reasonable, but filter type (e.g. "4th-order Butterworth") and passband should be specified somewhere.

Response: the data were filtered using FieldTrip's `ft_preprocessing` function with default settings, i.e. a 4th-order Butterworth filter applied bidirectionally using zero-phase filtering. We have clarified this in the Methods section (lines 638-640):

> *Unless stated otherwise, all filtering was performed using the FieldTrip function ft_preprocessing with default settings (4th-order Butterworth IIR filter, applied bidirectionally using zero-phase filtering).*

And in the legend of Fig. 8B:

> *Black: raw signal; blue: signal filtered in the beta1 band (11.2–15.8 Hz)*

Reviewer's comment 2.06:

Figs. 8C-D: This set of results is now easily understandable, and the question that comes immediately to mind is, "Are these changes statistically significant?" Legend states: "Trace plotted to the right of the panel depicts the mean power during the first 200 ms after TMS (C) or baseline rhythmicity during the [-1 -0.5] s baseline window (D)." However, no scales are given for the traces, and there is no statement as to what the black outlines in the pseudocolor plots mean. Are they outlines of statistically significant regions? At what p level? The CLs around the traces "to the right" would give some hints to answer these questions if there were a scale bar and an explanation as to what kind of CLs they are (95% Confidence? Plus/minus SEM? Plus/minus SD?).

Response: we thank the reviewer for this careful and helpful comment. We have amended the figure to include explicit scales for the side traces and to clarify the nature of the CLs, which are now labelled as mean ± SEM (see updated Fig. 8C–D). We now also clearly define the CLs in the legend (line 468):

> *Trace plotted to the right of the panel depicts the mean ±SEM*

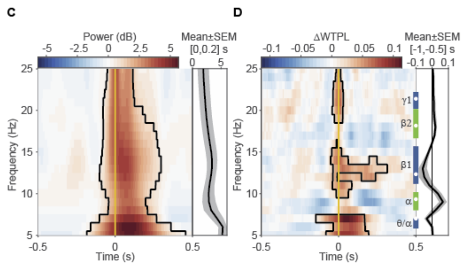

Regarding the black outlines in the pseudocolour plots, we have clarified the figure legend to state that they denote statistically significant clusters at the p < 0.05 level:

> *Black contour denotes clusters with p < 0.05 (cluster-based permutation test over t-values).*

To ensure this point is unambiguous, we have revised the legend (lines 470-471) to read:

> *Black contour denotes clusters with significant increased power or rhythmicity (p < 0.05, cluster-based permutation test over t-values).*

Overall, we agree that these amendments facilitate interpretation of the results.

<u>Reviewer's comment 2.07:</u>
New lines 632-646 present some interesting ideas, but it is hard to tease apart the biology and the analysis technology. On the one hand, the band-stopped signals are a technical manipulation that shows how strong oscillations in neighboring bands could potentially confuse the LAVI algorithm. Rather than disrupting oscillations in neighboring bands, it masks them by creating peaks and valleys that are similar to those that may occur in-band. On the other hand, biological mechanisms that are sensitive to rhythm could similarly get confused. It would be good to point this out so that readers don't dismiss the point as "just a technical artifact".

Response: we thank the reviewer for this thoughtful comment and agree that it is important to clearly distinguish between signal-level effects arising from the analysis and their possible biological relevance. As the reviewer notes, the band-stop manipulation is a technical operation that demonstrates how strong oscillatory activity in one band can influence rhythmicity estimates in neighbouring frequency ranges by shaping the measured signal. In response to related concerns raised by Reviewer 1 (comment 1.14), we explicitly tested whether the observed low rhythmicity in neighbouring bands could be explained trivially by such interference effects. As shown in Supplementary Fig. S5, beta1 troughs are significantly more arrhythmic than expected from interference alone, based on both analytically constructed surrogates and subject-specific simulations.

Importantly, we do not interpret this result as evidence for direct disruption or suppression of neuronal oscillations in adjacent bands. Rather, as now clarified in the Discussion (lines 559–564), our interpretation is explicitly restricted to the level of the measured rhythmicity profile. At the same time, we believe that these signal-level

interactions are not merely a technical artefact to be dismissed, as biological mechanisms sensitive to rhythmic structure could, in principle, be influenced by similar phase-based interactions. Determining whether and how such mechanisms operate at the cellular or circuit level will require future invasive or intracellular recordings, which are beyond the scope of the present study.

We have revised the text accordingly to make this distinction explicit, so that readers can appreciate both the analytical implications and the potential biological relevance without conflating the two. Discussion section (lines 559-564):

> *We observed that high-rhythmicity bands can attenuate rhythmicity in adjacent frequency ranges via phase interference at the level of the measured signal (Fig. 3A; see Fig. S5). Whether such interactions reflect cellular-level mechanisms will require future intracellular recordings and is beyond the scope of the present study. At the level of the measured signal, ongoing oscillatory activity in a dominant high-rhythmicity band may curtail the development of nearby rhythms through phase-based interactions (i.e., interference).*

Reviewer's comment 2.08:
Fig. 3A: the gray lines are very hard to see, especially in the averages at the bottom.

Response: we thank the reviewer for pointing this out. We have increased the thickness of the grey band-stop–filtered traces in the averaged burst-related potentials, to improve visibility. The figure legend has been updated accordingly:

> *Black: raw signal (one representative subject). Blue/green: band-pass–filtered. Grey: band-stop filtered (neighbouring bands removed). Bottom row: average across bursts ("burst-related potential", BRP). Grey traces are plotted thicker for visibility.*

Signed,

Daniel J. Gibson

Reviewer #3 (Remarks on code availability):

I have only very briefly glanced at the code, primarily to make sure that it is easily accessible as stated on Github. It appears to be well organized and clearly documented, but I have not analyzed it in any detail.
